# Open-label, randomised controlled, multicentre study evaluating higher valency pneumococcal vaccines (PCV15/PCV20) compared with PCV13 given in homologous schedules at 3 months and 12 months (‘1+1’ schedule) and 2 months, 4 months and 12 months (‘2+1’ schedule) in infants: protocol

**DOI:** 10.1136/bmjopen-2025-116062

**Published:** 2026-09-22

**Authors:** Riffat Aslam, Oluwasefunmi Akeju, Elizabeth Clutterbuck, Charlie Appleby, Denis Murphy, Alix De Calignon, Jilly Muller, Rachel White, Ankana Singha, Yama F Mujadidi, Marion Roderick, David P J Turner, Paul T Heath, Daniel Owens, David Goldblatt, Narges Ebrahimi, Xinxue Liu, Saul Faust, Maheshi Nirmala N Ramasamy, Dominic Kelly

**Affiliations:** 1 Department of Paediatrics, Oxford Vaccine Group, Centre for Clinical Vaccinology & Tropical Medicine (CCVTM), University of Oxford, Oxford, England, UK; 2 Bristol Vaccine Centre, L6 Education & Research Centre, Bristol, UK; 3 Cripps Health Centre, The University of Nottingham Health Service, Nottingham, UK; 4 Vaccine Institute & Centre for Neonatal and Paediatric Infection, City St. George’s, University, London, UK; 5 NIHR Southampton Clinical Research Facility, Southampton General Hospital, University Hospital Southampton NHS Foundation Trust, Southampton, UK; 6 University of Southampton Faculty of Medicine, Southampton, UK; 7 Great Ormond Street Institute of Child Health, University College London, London, UK; 8 Oxford BRC, OUH NHS Foundation Trust, National Institute for Health and Care Research, London, UK

**Keywords:** Child, Immunization Programs, Community child health, Paediatric infectious disease & immunisation, Vaccination

## Abstract

**Background:**

Most recent childhood invasive pneumococcal disease in the UK is caused by serotypes not included in the currently used pneumococcal conjugate vaccine (PCV13). There is potential for additional protection with newer PCVs that have wider serotype coverage but there are no data on these vaccines being given in the 1+1 schedule used in the UK. This study will determine whether the PCV13 serotype-specific antibody responses to PCV15 and PCV20 given at 3 and 12 months (1+1 schedule) are non-inferior to PCV13 serotype-specific antibody responses to PCV13 given in the same schedule. It will also determine whether PCV13 serotype-specific antibody responses to PCV20 given at 2, 4 and 12 months (2+1 schedule) are non-inferior to PCV13 serotype-specific antibody response to PCV13 given at 3 and 12 months (1+1 schedule).

**Methods:**

This will be an open-label, randomised controlled multicentre study in 600 infants. Healthy infants will be enrolled at 8 weeks and randomised 1:1:1:1 to receive 2 doses (1+1) of either PCV15, PCV20 or PCV13 at 3 and 12 months of age or 3 doses (2+1) of PCV20 at 2, 4 and 12 months. Blood and nasal samples will be collected at specified intervals to assess serum and nasal IgG levels and carriage. Postprimary and postbooster IgG levels in the PCV15 and PCV20 arms will be compared against PCV13.

**Ethics and dissemination:**

Ethical approval for this study has been obtained from the South Central—Hampshire A Research Ethics Committee (REC ref 24/SC/0222). The results will be disseminated via publication in a peer-reviewed journal and presentations at international conferences.

**Trial registration number:**

ISRCTN17673117.

**STRENGTHS AND LIMITATIONS OF THIS STUDY**

- The study specifically evaluates the 1+1 schedule for which no immunogenicity data currently exist for pneumococcal conjugate vaccine (PCV15) or PCV20 in infants, addressing an important evidence gap.
- The open-label design may introduce reporting bias for reactogenicity.
- The study sample size is not sufficient to give data on vaccine efficacy.

## Introduction

### Background and rationale

*Streptococcus pneumoniae* causes pneumonia and invasive pneumococcal disease (IPD), including sepsis and meningitis, especially in children and older adults.^1 2^ There were 378 cases of IPD among children aged 0–14 years in the UK from April 2022 to March 2023.^1^ *S. pneumoniae* commonly colonises the nasopharynx of healthy young children, and asymptomatic pneumococcal carriage has been observed in nearly half of children aged 1–2 years in a study within the Thames Valley region of the UK.^3^ However, nasopharyngeal carriage is also a precursor to disease and transmission.^4^ There are at least 100 serotypes of *S. pneumoniae*,^5^ which have varying capacities to cause disease.^6^ Immunity derived from pneumococcal conjugate vaccine (PCV) tends to be serotype specific with little or no crossover.^7^

Since the UK introduced the seven-valent PCV7, containing serotypes 4, 6B, 9V, 14, 18C, 19F and 23F, in 2006, childhood IPD caused by vaccine serotypes has declined.^1^ In 2010, the UK switched from PCV7 to PCV13, which includes six additional serotypes (4, 6B, 9V, 14, 18C, 19F and 23F). PCV13 was introduced in a 2+1 schedule (two priming doses and a booster) and was later changed to a 1+1 schedule (priming and booster doses at 3 and 12 months) in 2020. This decision was based on findings from a postlicensure trial that demonstrated non-inferior postbooster immunogenicity of 1+1 compared with the 2+1 schedule.^8^ Vaccine failure or breakthrough infections have not increased since switching to the reduced schedule; however, approximately 80% of childhood IPD in the UK is now caused by non-PCV13 serotypes.^1^ Similarly, the predominance of non-PCV13 serotypes was observed in healthy carriage among vaccinated children.^3^ This evidence supports the need to assess whether switching to any of the new higher-valency PCVs with wider serotype coverage could further reduce childhood IPD.

In addition to PCV13 serotypes, PCV15 and PCV20 include two (22F and 33F) and seven (8, 10A, 11A, 12F, 15B, 22F and 33F) other serotypes, which account for an additional 13% and 42% of childhood IPD, respectively.^1^

PCV15 and PCV20 were recently approved for use in infants in the USA^9^ and the EU,^10 11^ based on non-inferiority of immunogenicity to PCV13 for the PCV13-shared serotypes.^12 13^ The European Medicines Agency licensed PCV13 and PCV15 in a 2+1 schedule whereas PCV20 was licensed in a 3+1 schedule, as it failed to achieve non-inferiority of immunogenicity to PCV13, after a 2+1 schedule.^14^ PCV15 was licensed in the UK by the Medicines and Healthcare products Regulatory Agency (MHRA) in 2022 for use from 6 weeks of age in a 1+1 schedule^15^ while PCV20 (APEXXNAR/PREVENAR 20) has been licensed in the UK from 24 September 2024 as a 2+1 and 3+1 schedule from 6 weeks to 15 months of age. Although non-inferiority criteria were not met for some serotypes in PCV20, licensing decisions were based on the overall evidence, including safety, immunogenicity and expanded serotype coverage. Both of these vaccines are not yet part of the routine immunisation schedule. PCV13 continues to be used in the childhood immunisation programme.^16^ There is no real-world evidence on the efficacy of PCV15 and PCV20 against childhood IPD or carriage. Although PCV15 and PCV20 may reduce IPD caused by non-PCV13 serotypes, the reduction in individual serotype immunogenicity with increasing valency makes it uncertain whether either vaccine would have a greater impact on overall IPD than PCV13, especially if used in a reduced schedule.^17 18^ In fact, predictions from a modelling study suggest a possible increase in IPD following a switch from PCV13 to PCV15 because the anticipated gains from eliminating the two additional serotypes could be nullified by a predicted replacement with more invasive non-PCV15 serotypes.^18^

These uncertainties about the potential impact of new PCVs provide justification for comparing the immunogenicity of PCV15 and PCV20 against PCV13, at the reduced schedule currently used in the UK. Evidence generated from a head-to-head comparison of these PCVs would inform decisions on the UK PCV schedules.

### Study aims and objectives

This study aims to compare the immunogenicity and safety of PCV15 and PCV20 vaccines when administered in different dosing schedules with PCV13 vaccine as the control. The study comprises four arms. Participants in arms 1, 2 and 4 receive a two-dose schedule of PCV15, PCV20 and PCV13, respectively, at 3 and 12 months of age, whereas participants in arm 3 receive a three-dose schedule of PCV20 at 2, 4 and 12 months of age. Primary, secondary and exploratory objectives and outcomes are outlined in table 1.

**Table 1:** Primary, secondary and exploratory objectives and outcomes of PCV15/PCV20 study

Objectives	Outcome measures
Primary objective*:* To determine if the PCV13 serotype-specific serum IgG concentrations are non-inferior in homologous arms 1 and 2 in comparison to the control arm 4.	PCV13 serotype-specific serum IgG concentration at 13 months of age.
Secondary objectives: To determine if the PCV13 serotype-specific serum IgG concentrations are non-inferior in arm 3 in comparison to the control arm 4.	PCV13 serotype-specific serum IgG concentration at 13 months of age.
To characterise the seven additional PCV20 serotype-specific serum IgG concentrations following homologous PCV schedules.	PCV20 serotype-specific serum IgG concentrations (Serotypes 8, 10A, 11A, 12F, 15B, 22F and 33F) at 13 months of age.
To determine the safety and reactogenicity of different schedules with PCV15 (1+1) and PCV20 (1+1) and (2+1).	Solicited AEs within 7 days Unsolicited AEs within 28 days *SAEs during the whole study period.
Exploratory objectives: To determine if the vaccine induces IgG response at the mucosa (nasal) and if antibody levels in the nose correlate with levels in blood.	PCV13 serotype specific nasal IgG GMC against some of the PCV serotypes (sample volume depending) at 13 months of age.
To determine the PCV20 serotype-specific IgG concentration following one dose of PCV13, PCV15 or PCV20.	PCV20 serotype-specific serum IgG concentrations at 4 months.
To determine the PCV20 serotype-specific IgG concentration following two doses of PCV20 (2+1) schedule.	PCV20 serotype-specific serum IgG concentrations at 5 months.
To detect pneumococcal colonisation and serotype present.	Molecular microbiology (fluidic qPCR for detection of lytA, paiB and serotyping) at 13 months of age.
To determine immunogenicity against concomitant vaccines given as part of the infant routine immunisation schedule.	IgG antibody GMCs concentration against pertussis antigen (pertussis toxin, filamentous haemagglutinin and pertactin), diphtheria toxoid, tetanus toxin and Hib capsular polysaccharide; SBA GMT against MenB at 13 months of age.

AEs, adverse events; GMC, geometric mean concentrations; PCV, pneumococcal conjugate vaccine; SAEs, Severe Adverse Effects; SBA GMT, serum bactericidal antibody (SBA) geometric mean titres (GMTs).

## Methods

### Study design

This is an open-label, randomised controlled, multicentre study with a sample size of 600 participants recruited and assigned to four arms of 150 participants each.

### Study timeline

Healthy children are enrolled at approximately 2 months (+2 weeks) of age. Participants in arms 1, 2 and 4 will receive two doses of either PCV15, PCV20 or PCV13 at 3 and 12 months of age, while arm 3 will follow a 2+1 schedule and will receive two doses of PCV20 at 2 months and 4 months, followed by a booster dose at 12 months. Blood and nasal samples will be collected throughout the study according to the allocated study arm, and the time points for each have been specified in table 2.

**Table 2:** Study schedule for vaccines and sampling

Visit number	V1	V2	V3	V3 (A)	V4	V5
Participant age	2 months	3 months	4 months	5 months	12 months	13 months +
Blood sample for arms 1, 2, and 4	–	–	Yes		–	Yes
Blood sample for arm 3				Yes		Yes
Nasal sample (mucosal lining fluid) for arms 1, 2 and 4		Yes	Yes		–	Yes
Nasal sample (mucosal lining fluid) for arm 3	Yes	Yes		Yes		Yes
Routine vaccines arms 1–4	6:1 MenB Rotavirus	6:1 Rotavirus	6:1 MenB		MenB Hib/MenC MMR	–
Pneumococcal vaccines arm 1	–	PCV15	–		PCV15	–
Arm 2	–	PCV20	–		PCV20	–
Arm 3	PCV20		PCV20		PCV20	–
Arm 4	–	PCV13	–		PCV13	–

MMR, Measles Mumps Rubella; PCV, pneumococcal conjugate vaccine.

This study opened to recruitment from 3 December 2024, when the first participant was enrolled. Recruitment is expected to be completed by October 2026.

### Protocol amendment affecting PCV schedule

From 1 July 2025, during study enrolment, the national immunisation schedule changed with the second dose of MenB now given at 3 months (previously 4 months) and in consequence the first dose of PCV was to be given at 4 months (previously 3 months). In order to provide some data on the revised schedule for the broader valency PCV vaccines, a substantial amendment was submitted (substantial amendment 3.0: 16 May 2025, approved 24 June 2025) which included comparing the effect of giving the single PCV dose at 3 vs 4 months. This introduced a second randomisation for arms 1, 2 and 4 allocating participants within these arms to receive PCV at 3 or 4 months with the blood sample 1 month after either time point (see online supplemental document: major protocol amendment). The primary endpoint was unchanged by this amendment. At the point of implementation of this amendment 237 infants had already been enrolled into the study. A complete amendment history is provided as online supplemental material.

### Study setting

Potentially eligible children whose parents are interested in the study will be visited either at home or in a clinic, depending on site preference, where informed consent will be sought and infants enrolled/immunised as appropriate by the clinical team. A copy of participant informed consent form is provided as online supplemental material.

### Recruitment and eligibility

Potential participants will be identified and approached using the following recruitment strategies: email, posters, electronic newsletter, leaflets, websites, advertisement in newspapers, radio and on social media and/or mail-out using an approved invitation letter or other approved advertising material to invite them to participate in the study. Parents will be asked to complete a prescreening online questionnaire to assess participants’ eligibility, which will then be assessed by study staff. If interest comes through reply slip, parents will be contacted via phone or email to assess eligibility.

The inclusion, exclusion and temporary exclusion criteria that will be used to screen participants prior to enrolment and before each study visit are compiled in boxes 1–3.

Box 1 **Inclusion criteria:**

- Infants due to receive their primary immunisations, aged up to 2 months (+ 2 weeks) at first vaccinations.
- Infants born at ≥ 37 weeks of gestational age.
- Parent(s) or legal guardian(s) willing and able to follow the requirements of the protocol for the duration of the study.
- Written informed consent provided by parent or legal guardian aged ≥16 years.

Box 2 **Exclusion criteria:**

- Prior receipt of vaccines. (Except of Hepatitis B or BCG vaccine).
- Prior planned receipt of investigational vaccines.
- Current participation in another research study, except if the study is solely observational.
- Children of parents who are on the delegation log for this study.
- A confirmed anaphylactic reaction to neomycin, streptomycin or polymyxin B (which may be present in trace amounts in the tetanus vaccine) and/or kanamycin, histidine, sodium chloride or sucrose (which may be present in trace amounts in the MenB vaccine).
- Latex hypersensitivity (the syringe cap of Bexsero may contain natural rubber latex).
- Major congenital defects or serious chronic illness.
- Presence of an evolving or changing neurological disorder.
- Presence of central nervous system disease or convulsions in the infant.
- Bleeding disorder.
- Confirmed or suspected immunodeficiency.
- A family history of congenital or hereditary immunodeficiency.
- Receipt of more than 1 week of immune-suppressants or immune-modifying drugs (e.g., oral prednisolone >0.5 ml/kg/day or intravenous glucocorticoid steroid). Nasal, topical or inhaled steroids are allowed.
- Administration of immunoglobulin and/or any blood products since birth or planned administration during the study period.
- History of allergy to any component of the vaccines. Any other significant disease or disorder which, in the opinion of the Investigator, may either put the participants at risk through participation in the study, or may influence the result of the study or the participant’s ability to participate in the study.

Box 3 **Temporary exclusion criteria:**

Participants may be temporarily excluded from participating if they have any of the following:

- Have received BCG and Hepatitis B vaccine within 14 days prior to study vaccines.
- Have scheduled elective surgery, planned admission or other procedures requiring general anaesthesia within 7 days of receiving a vaccine.
- In the presence of an acute illness or the presence of fever ≥38ºC, until 24 hours after resolution.
- Blood sampling will also be postponed for seven days after completion of any antibiotic course to ensure no interference with laboratory tests.

### Trial interventions

#### Investigational medical products

PCV13: PREVENAR13 (Pfizer) a PCV that contains 13 different pneumococcal serotypes and is licensed in Europe. It is part of the UK routine schedule at 3 and 12 months of age. This vaccine is only going to be administered at 3 and 12 months of age. One dose (0.5 mL) will be injected at each vaccination.

PCV15: VAXNEUVANCE (MERCK) is a licensed vaccine to be used in the UK. It is indicated for active immunisation in individuals 6 weeks of age and older. One dose (0.5 mL) will be injected at each vaccination.

PCV20: APEXXNAR/PREVENAR-20 (Pfizer) is a pneumococcal vaccine licensed for use from 6 weeks of age onwards. One dose (0.5 mL) will be injected at each vaccination.

PCV13 will be obtained as part of the routine supply from the UK national immunisation programme.

While PCV15 and PCV20 will be obtained directly from the manufacturers.

Table 3 lists the other non-investigational medical products (IMPs) used in the trial to be given as part of routine infant immunisation schedule.

**Table 3:** Non-investigational medical products

Vaccine	Brand
DTaP-IPV-Hib- Hep B (6-in-1)	Infanrix hexa or vaxelis
MenB	Bexsero
Rotavirus	Rotarix
Hib/MenC	Menitorix
MMR	MMRvaxPro or Priorix

MMR, Measles Mumps Rubella.

### Participant timeline

Enrolment for all participants will begin at 2 months of age, with the final visit occurring at 13 months of age.

### Sample size

The primary analysis of this study will assess the non-inferiority of homologous PCV15/PCV20 schedules to PCV13. Based on prior data, assuming an SD of 0.5 for PCV13 serotypes-specific IgG concentrations on the log₁₀ scale and a non-inferiority margin of 0.67, as recommended by WHO guidelines on clinical evaluation of vaccines,^8^ an effective sample size of 131 participants per arm is needed. After adjusting for an attrition of 10%–15%, the study will recruit a total of 600 participants to achieve 80% power at a two-sided 0.05 significance level. No adjustment for multiple comparisons is made, and non-inferiority will be assessed for each of the 13 serotypes. The overall conclusion regarding non-inferiority will also take the epidemiological data into consideration, as non-inferiority may not be met for all serotypes due to variability.

### Trial procedures

Enrolment into the study will occur when parents have provided consent on behalf of their child and a face-to-face eligibility check has been completed.

### Randomisation

Participants will be randomised to a 1:1:1:1 ratio before their first vaccination visit into four arms to receive PCV vaccine as per the trial design mentioned in table 2. Computer-generated randomisation lists will be prepared by the study statistician at the Oxford Vaccine Group (OVG) using stratified block randomisation. Random block sizes of 4 or 8 will be used. The randomisation lists will be stratified by study site. To ensure allocation concealment, an online randomisation system (REDCap, version 16.0.17) was used, with randomisation lists preloaded into the system.

### Blinding

This is an open-label trial, and neither participants’ parents, investigators, nor laboratory personnel processing the samples are blinded to treatment allocation. However, the pneumococcal serum assays will be performed in an external laboratory, where samples will be analysed using anonymised identifiers with no access to treatment allocation. This ensures that personnel involved in the measurement of immunogenicity outcomes remain blinded, thereby minimising the risk of measurement bias.

### Study visits

A total of five study visits will be conducted for participants enrolled in arm 1, 2 and 4 and 6 visits for participants in arm 3. Table 2 provides an overview of visit details, vaccination schedules and sample requirements for each visit. After obtaining the parents’ written informed consent, all participants will be enrolled in the study at visit 1. A thorough check of the inclusion and exclusion criteria will be undertaken, and the findings, including relevant medical and vaccination history, will be recorded in the database. A physical examination will be performed by a study doctor if a 6–8-week health development review has not been undertaken previously or at the investigator’s discretion if indicated by the medical history. All participants, after receiving IMPs, will be issued a 7-day (days 0–6) eDiary to record temperature and local or systemic reactions.

### Sample collection

Within the study period, there are a few time points where blood and nasal samples are collected. Time points for sampling will depend on the arm the participant is randomised to. There are two time points for blood samples. A maximum of 4 mL, 5 mL and 6 mL blood will be obtained at visit 3, 3A and V5, respectively. A topical anaesthetic cream will be offered prior to each venepuncture.

Nasal samples will be collected at three time points for all participants. Participants randomised to arm 3 will have one additional nasal sample collected. Mucosal lining fluid will be collected using synthetic absorptive matrices, with the strip held in place for 30 to 60 s for better absorption.

### Laboratory testing

Depending on the arm allocation for each participant, two blood samples and three or four nasal samples will be taken throughout the study. Samples will be shipped to a laboratory at a separate site for assessment of serotype specific IgG concentration for PCV13, PCV15 and PCV20. Further details for sampling and laboratory analysis are provided in online supplemental material.

### Data collection

All study documents will be stored safely in accordance with the Data Protection Act 2018 and UK General Data Protection Regulation (UK GDPR). Data collected at each visit will be recorded directly into an electronic data capture (EDC) system (REDCap) hosted on a secure, password-protected server with role-based access controls or onto paper source documents for later entry into the EDC, if direct entry is not available. Paper records will be stored securely and will only be accessible to authorised members of the central study team.

The study database will include audit trails to track data entry and modifications, and regular backups will be performed. Access to data will be restricted to authorised personnel only.

Parents or legal guardians will provide consent for their email addresses to be entered into an electronic diary system, allowing them to complete the diary online. The database incorporates predefined data types and logical constraints to facilitate real-time validation of data. The data management and monitoring teams will conduct continuous oversight of data collection and storage processes throughout the duration of the study.

### E-diary/paper diary

Parents or legal guardians will be provided with an online electronic diary following each PCV vaccination. The diary will be used to record daily temperature measurements and any local and systemic solicited adverse reactions from the day of vaccination through to 6 days postvaccination. Parents will also be provided with a paper diary as a backup in case electronic entry is not possible. Completed paper diaries will be collected at the next study visit, and the data will be entered into the study database by site staff. Paper records will be retained at the study site and stored in the Investigator Site File at study completion.

### Data access

Direct access will be granted to authorised representatives of the Sponsor, host institution and the regulatory authorities to permit trial-related monitoring, audits and inspections. Access to medical records by those outside the direct healthcare team will only occur if consent has been received. This would be for the purpose of clarifying medical history in relation to the inclusion/exclusion criteria and safety reporting.

### Early discontinuation or withdrawal of participants

Parents or legal guardians may withdraw their child from the study at any time and for any reason. The investigator may also discontinue a participant if necessary, including due to ineligibility, protocol deviation, non-compliance, adverse events (AEs), disease progression, withdrawal of consent or loss to follow-up. Data collected prior to withdrawal will remain included in the study analysis. Parents or legal guardians may request destruction of their child’s samples unless they have already been analysed. The reason for withdrawal will be recorded in the Case Report Form (CRF), where available.

### Safety reporting

Safety reporting for the study will commence once the participant has been enrolled following informed consent. It will end following the final study visit (V5). All solicited local and systemic AEs and participant’s axillary temperature, observed by parents or legal guardians will be reported in the eDiary daily for 7 days following vaccination with IMP vaccines. All reported Severe Adverse Events (SAEs) will be recorded over the duration of the study. All other details about safety reporting are provided in online supplemental material.

### Monitoring

The OVG will oversee study conduct and protocol compliance, as delegated by the sponsor. OVG’s Quality Assurance team will perform regular monitoring, as outlined in the study monitoring plan provided in online supplemental material, to ensure the trial is conducted and that the data are accurate and compliant with the protocol, Good Clinical Practice and regulatory requirements

### Interim analyses

There will be no interim analysis.

### Statistical analysis

The primary outcome is the PCV13 serotype-specific IgG 1 month after the booster dose. The geometric mean concentrations (GMC) of IgG will be compared between homologous PCV15 and PCV20 arms and homologous PCV13 arm (as the reference) under the hypothesis:

H0: GMCPCV15/PCV20/GMCPCV13 ≤0.67 or *log*10(GMCPCV15/PCV20)*—log*10(GMCPCV13) ≤−0.174;

H1: GMCPCV15/PCV20/GMCPCV13 >0.67 or *log*10(GMCPCV15/PCV20)*—log*10(GMCPCV13) >−0.174.

The PCV13 serotype-specific IgG concentration will be transformed using logarithmic transformations (base 10) to render a normal distribution. We will test the above hypothesis using the multiple regression on *log*10GMC adjusting for randomisation design variable (site), and the prespecified prognostic factors, including sex, gestational age at birth, birth weight and ethnicity. The adjusted geometric mean ratio will be presented with the two-sided 95% CI. We will claim homologous PCV15/PCV20 arms are non-inferior to homologous PCV13 arm if the lower CI lies above 0.67.

The primary analysis will be conducted on a per-protocol basis among participants whose primary endpoint at D28 postboost is available, as the intention-to-treat analysis is no longer producing a conservative estimation in non-inferiority trials. The modified intention-to-treat analysis will also be conducted as a sensitivity analysis. The primary analysis will be carried out when the primary endpoint of D28 PCV13 serotype-specific IgG data become available.

The extent and pattern of missing baseline and outcome data will be reported. Where possible, reasons for missing data will be explored and documented. If missing primary outcome data are ≤10%, a complete-case analysis will be used; if >10%, multiple imputation under a missing at random assumption will be applied.

### Confidentiality

The study staff will maintain participant confidentiality by identifying them only by ID numbers on study documents and databases, except for the CRF (which includes participant initials) and the signed consent form. Documents will be securely stored and accessible only to authorised personnel. Parent or legal guardian email addresses are needed for electronic diaries and will only be accessible by relevant site staff and sponsor data managers. The study will comply with GDPR, ensuring data are anonymised as soon as possible.

### Approvals

The protocol, informed consent form, study information booklet, advertising materials and any other supporting trial documents will be submitted for written approval to the appropriate Research Ethics Committee (REC), the HRA (if applicable), regulatory authorities (including the MHRA in the UK) and host institution(s). Approval will be sought from all relevant parties for any amendments to the study documents prior to implementation.

### Patient and public involvement

The protocol, study information booklet and recruitment materials were reviewed by an individual from the OVG PPI group who provided feedback and comments on the initial documents. Their comments led to changes in the participant-facing documents, ensuring they are clear and easy for parents to understand.

## Ethics and dissemination

Ethical approval for this study has been obtained from the South Central–Hampshire A Research Ethics Committee (REC reference 24/SC/0222). The trial protocol is registered on the ISRCTN registry (ISRCTN17673117). The trial is conducted in accordance with the Declaration of Helsinki, Good Clinical Practice (GCP) guidelines and applicable UK regulatory requirements. Written informed consent is obtained from a parent or legal guardian before enrolment. Participant confidentiality is maintained in compliance with the General Data Protection Regulation (GDPR), with data stored securely on password-protected systems and accessible only to authorised personnel.

Dissemination of the findings is planned through publication in peer-reviewed journals and presentations at international scientific conferences.

### Other ethical consideration

Any participant found to have an antibody response <0.35 µg/mL against all PCV serotypes in the postbooster blood, taken at 13 months of age, will be contacted to discuss the results and, with parental permission, will be referred for further investigation.

## Supplementary material

10.1136/bmjopen-2025-116062 online supplemental file 1

10.1136/bmjopen-2025-116062 online supplemental file 2

10.1136/bmjopen-2025-116062 online supplemental file 3

10.1136/bmjopen-2025-116062 online supplemental file 4

10.1136/bmjopen-2025-116062 online supplemental file 5

10.1136/bmjopen-2025-116062 online supplemental file 6
