# Supplementary material for: Open-label, randomised controlled, multicentre study evaluating higher valency pneumococcal vaccines (PCV15/PCV20) compared with PCV13 given in homologous schedules at 3 months and 12 months (‘1+1’ schedule) and 2 months, 4 months and 12 months (‘2+1’ schedule) in infants: protocol
Source: BMJ Open. 2026 Sep 22;16(9):e116062. doi: 10.1136/bmjopen-2025-116062 (PMC13599847; doi:10.1136/bmjopen-2025-116062)
Supplement: Supplementary file 1 [file bmjopen-16-9-s001.pdf]

All Amendments Versions and details

Amendment SA02 V2.0 18Sep24 – approved 23 Oct 2024 – PCV20 name changed from Apexxnar to Prevenar in all relevant study documents to reflect change in commercial name of the vaccine in the UK.

AmendmentSA03 V3.0 16May25 – approved 24 Jun 2025 – in view of planned changes to the UK immunisation schedule on 1 July 2025: a **second randomisation** was introduced for study arms 1, 2 and 4 (single dose PCV15, 20 and 13 respectively) to allow a comparison between single dose PCV administered at 3 versus 4 months given the change to giving PCV routinely at 4-months; and the **Hib/MenC vaccine given at 12-months of age** was removed from the protocol following its withdrawal from the UK infant immunisation schedule.

Amendment SA04 V4.0 25Jul25 – approved 15 Sep 2025 – additional updates were made to align with the **revised UK immunisation schedule effective 1 July 2025,** including the **MenB second dose** being given at **3 months of age** rather than the previous 4-months.

Amendment SA05 V5.0 03Oct25 – approved 19 Nov 2025 – Changes made to reflect upcoming updated UK immunisation schedule with the possible inclusion of the MMRV vaccine to be administered once the national immunisation schedule is updated. Current version of the protocol (V5.0, 02 Oct 2025) is provided as supplementary material.
