# Supplementary material for: Open-label, randomised controlled, multicentre study evaluating higher valency pneumococcal vaccines (PCV15/PCV20) compared with PCV13 given in homologous schedules at 3 months and 12 months (‘1+1’ schedule) and 2 months, 4 months and 12 months (‘2+1’ schedule) in infants: protocol
Source: BMJ Open. 2026 Sep 22;16(9):e116062. doi: 10.1136/bmjopen-2025-116062 (PMC13599847; doi:10.1136/bmjopen-2025-116062)
Supplement: Supplementary file 2 [file bmjopen-2025-116062.reviewer_comments.pdf]

PEER REVIEW HISTORY

BMJ Open publishes all reviews undertaken for accepted manuscripts. Reviewers are asked to complete a checklist review form and are provided with free text boxes to elaborate on their assessment. These free text comments are reproduced below.

ARTICLE DETAILS

Title (Provisional)

Protocol: Open-label, randomised controlled, multicentre study evaluating higher valency pneumococcal vaccines (PCV15/PCV20) compared to PCV13 given in homologous schedules at 3 months and 12 months ("1+1" schedule) and 2 months, 4 months and 12 months ("2+1" schedule) in infants

Authors

Aslam, Riffat; Akeju, Oluwasefunmi; Clutterbuck, Elizabeth; Appleby, Charlie; Murphy, Denis; De Calignon, Alix; Muller, Jilly; White, Rachel; Singha, Ankana; Mujadidi, Yama F; Roderick, Marion; Turner, David P.J.; Heath, Paul T; Owens, Daniel; Goldblatt, David; Ebrahimi, Narges; Liu , Xinxue; Faust, Saul; Ramasamy, Maheshi Nirmala N.; kelly, dominic

---

VERSION 1 - REVIEW

**Reviewer**	**1**
**Name**	**Temple, Beth**
**Affiliation**	**Menzies School of Health Research**
**Date**	**24-Feb-2026**
**COI**	**None**

This protocol paper details a trial of PCV15 and PCV20 compared with PCV13 in the UK. The main manuscript is lacking some critical information and requires some clarifications prior to publication.

Major points:

1. Amendments, page 14 – Amendment 3.0 represents a major amendment, involving changes to the randomisation procedure and the timing of the study intervention. This should be described earlier in the manuscript and the study details (design, schedule, etc) should reflect both the original and the revised design. Information should also be provided on how many participants were enrolled prior to this amendment. There also needs to be discussion on how the validity of combining the groups (vaccinated at 3 or 4 months) will be assessed.

2. Sample size, page 11 – please provide justification/rationale for the choice of 0.67 for the non-inferiority margin (this is also not included in the full protocol). Please also clarify how an overall conclusion of non-inferiority will be made – you say "non-inferiority conclusion will consider all 13 serotypes", but there is no mention of how many serotypes should meet the criteria to draw this conclusion. As this relates to the primary outcome, it is important to pre-specify this.

3. Randomisation, page 11 – you say "a concealment approach will be employed" – please provide details here. The full protocol includes some additional information (which should be added into the manuscript) but still lacks detail on what information will be visible when the randomisation lists are accessed.

4. There is no information provided on blinding. Participants and clinic staff are clearly not blinded as this is an open-label trial, but information should be provided on the blinding of outcome assessors such as laboratory staff.

Clarifications:

1. Background (p6, lines 36-58) – this paragraph is not clear. For example, line 41 refers to the licensure of PCV13 when this paragraph provides information on PCV15 and PCV20 lincensure, and it is not clear which schedules have been licensed by which authority for which product. Lines 46-48 report that PCV15 is approved based on equivalence with PCV13, "so PCV13 continues to be used" – this implies that PCV13 is still being used because of the equivalence of PCV15, which seems contradictory. Please also provide the rationale for the different licensure of PCV20 in the UK compared with the EU, given that it did not meet non-inferiority criteria. Some of these details are included in the full protocol, but should be added to the manuscript for clarity.

2. There is no justification for the use of nasal samples rather than nasopharyngeal swabs, which are more typically used in PCV trials. Please provide the rationale for this choice of sampling, including whether it has been shown to perform in a similar way to NP swabs for identification of pneumococcal carriage.

3. Trial interventions, page 10 - the details for PCV20 licensing do not match the Background section

4. Data collection, page 11 – you say that "all documents will be stored safely in confidential conditions" – please provide details here. The back-up paper diary cards noted in the full protocol should also be mentioned here.

5. Statistical analysis, page 13, notes "adjusting for randomisation design variables and the pre-specified prognostic factors, if any". Please provide the details of the design variables and prognostic factors here. Similarly, the statement that "missing data will be handled using appropriate methods" is very generic – please expand on this. Also, what is the rationale for presenting the adjusted mean difference on the log scale, rather than the geometric mean ratio in line with other studies?

6. Confidentiality, page 13 – please explain why any site staff and data managers need access to the parent/guardian email addresses

7. Other ethical consideration, page 13 – will participants with low antibody responses be offered another dose of vaccine? It is not clear what is meant by "referred for further investigation".

8. Given the trial is underway, please provide dates of enrolment and how many participants have completed follow-up, etc.

9. Information on sample collection methods is missing from the manuscript – a summary should be provided in addition to the full information included in the full protocol.

10. The Safety Reporting document appears to be largely copied straight from the protocol, so it is not clear what this document adds. There are also issues with the table numbering in this document, and it references the groups 1r4, 2r4, etc from the amended protocol, which are not mentioned anywhere in the main publication. In this document (and the full protocol), it is stated that "the investigator will assess the maximum intensity… based on the investigator's clinical judgement". How is the maximum intensity different from the maximum severity as recorded by the parent/caregiver? This final assessment seems quite subjective and open to bias.

11. The Laboratory Analysis Plan still has some formatting track changes visible.

VERSION 1 - AUTHOR RESPONSE

Major points:

1. Amendments, page 14 – Amendment 3.0 represents a major amendment, involving changes to the randomisation procedure and the timing of the study intervention. This should be described earlier in the manuscript and the study details (design, schedule, etc) should reflect both the original and the revised design. Information should also be provided on how many participants were enrolled prior to this amendment. There also needs to be discussion on how the validity of combining the groups (vaccinated at 3 or 4 months) will be assessed.

Thank you for your comment. To highlight the major amendment 3.0 a section with heading "major protocol amendment" has now been added in the methods section of manuscript (lines 202 – 211). This section highlights the need for 3.0 amendment. Later in the manuscript, an appendix section covers more details about changes to primary and secondary outcome, randomisation and study design with in table 3, 4 and 4.1.

Line 216 and 217 also explains the number of participants enrolled prior to this amendment.

Evidence from previous studies (LionMenB unpublished) suggests that small shifts from 3 months to 4 months PCV in the timing of early infant vaccinations are unlikely to meaningfully impact immunogenicity at 13 months. Consistent with this, the validity of combining participants vaccinated at 3 and 4 months will be formally assessed in the analysis.

2. Sample size, page 11 – please provide justification/rationale for the choice of 0.67 for the non-inferiority margin (this is also not included in the full protocol). - Thank you for your comment. The non-inferiority margin of 0.67 was selected in line with the WHO Guidelines on clinical evaluation of vaccines: regulatory expectations, which recommend a lower bound of 0.67 for geometric mean ratios when assessing non-inferiority.

Please also clarify how an overall conclusion of non-inferiority will be made – you say "non-inferiority conclusion will consider all 13 serotypes", but there is no mention of how many serotypes should meet the criteria to draw this conclusion. As this relates to the primary outcome, it is important to pre-specify this.

Regarding the non-inferiority conclusion, we will assess non-inferiority for each of the 13 serotypes for both PCV15 and PCV20 separately. The overall conclusion on non-inferiority between PCV15/PCV20 and PCV13 will be informed by epidemiological data. As the local burden varies by country, some serotypes may not circulate in the UK; however, policy makers may still recommend the new schedule over the existing schedule based on the overall evidence. The text has been amended under Sample size (page 10 line 279) for clarity.

Randomisation, page 11 – you say "a concealment approach will be employed" – please provide details here. The full protocol includes some additional information (which should be added into the manuscript) but still lacks detail on what information will be visible when the randomisation lists are accessed.

Thank you for your comment. The text has been revised to clarify the concealment approach. Randomisation page 11, line 291-292

There is no information provided on blinding. Participants and clinic staff are clearly not blinded as this is an open-label trial, but information should be provided on the blinding of outcome assessors such as laboratory staff. Thank you for your comment. A separate heading "blinding" has now been added in the manuscript. Page 11 line 297-302

Clarifications:

1. Background (p6, lines 36-58) – this paragraph is not clear. For example, line 41 refers to the licensure of PCV13 when this paragraph provides information on PCV15 and PCV20 licensure, and it is not clear which schedules have been licensed by which authority for which product. Lines 46-48 report that PCV15 is approved based on equivalence with PCV13, "so PCV13 continues to be used" – this implies that PCV13 is still being used because of the equivalence of PCV15, which seems contradictory. Please also provide the rationale for the different licensure of PCV20 in the UK compared with the EU, given that it did not meet non-inferiority criteria. Some of these details are included in the full protocol, but should be added to the manuscript for clarity.

Thank you for your comment. The background section has now been updated to clearly explain the dates for PCV15 and PCV20 approvals in the UK. It also explains the rational for different licensure for PCV20. Page 5, paragraph 4.

2. There is no justification for the use of nasal samples rather than nasopharyngeal swabs, which are

more typically used in PCV trials. Please provide the rationale for this choice of sampling, including whether it has been shown to perform in a similar way to NP swabs for identification of pneumococcal carriage.

Nasal sampling via "SAM-strips" is well tolerated by infants in comparison to nasopharyngeal swab samples. This form of sampling allowed us to incorporate multiple samples (3-4) across the study time-points without compromising enrolment or increasing withdrawals because of a more uncomfortable sampling method. Moreover, SNOT study (PIDJ 2019) showed that detection of pneumococcus by SAM was similar to NP swabs and is now powered to look for protection against colonisation.

3. Trial interventions, page 10 - the details for PCV20 licensing do not match the Background section
Corrected - page 10, line 253

4. Data collection, page 11 – you say that "all documents will be stored safely in confidential conditions" – please provide details here. The back-up paper diary cards noted in the full protocol should also be mentioned here. Done – page 12

Thank you for your comment. This section has now been updated to included details about data storage. Another section below it with eDiary/Paper diary sub-heading has also been added to explain the purpose of it.

5. Statistical analysis, page 13, notes "adjusting for randomisation design variables and the pre-specified prognostic factors, if any". Please provide the details of the design variables and prognostic factors here. Similarly, the statement that "missing data will be handled using appropriate methods" is very generic – please expand on this. Also, what is the rationale for presenting the adjusted mean difference on the log scale, rather than the geometric mean ratio in line with other studies?

Thank you for your comments. We have added the design variables and prognostic factors to the statistical analysis section and explained the approach to handling missing data. We have also amended the text to report the geometric mean ratio (GMR), in line with other studies. Page 13, line 400.

6. Confidentiality, page 13 – please explain why any site staff and data managers need access to the parent/guardian email addresses.

Parent/legal guardian email addresses are required to enable study-related communication and to provide access to the electronic diary (eDiary) system. Email contact details are used solely for the purpose of issuing eDiary login credentials, sending automated reminders for diary completion, and communicating essential study information (e.g., visit reminders or follow-up queries). Email addresses is restricted to authorised site staff and designated data management personnel who require this information to issue the eDiary system and maintain study communication. All personal data is handled in accordance with applicable data protection regulations and stored securely with restricted access.

7. Other ethical consideration, page 13 – will participants with low antibody responses be offered another dose of vaccine? It is not clear what is meant by "referred for further investigation".
Referred to local paediatric team

Antibody testing in this study is conducted for research purposes and is not intended to guide individual clinical management. Therefore, participants will not routinely be offered an additional

vaccine dose on the basis of study immunogenicity results. The term "referred for further investigation" refers to the unlikely situation in which results suggest a possible underlying immunological abnormality (for example, consistently absent or markedly low responses across multiple serotypes). In such rare cases, and in consultation with the study investigator, the participant may be referred to their general practitioner or an appropriate clinical service for further assessment, in line with standard clinical practice.

8. Given the trial is underway, please provide dates of enrolment and how many participants have completed follow-up, etc. This information has now been added in the methods section of manuscript. Page 8, line 216-217

9. Information on sample collection methods is missing from the manuscript – a summary should be provided in addition to the full information included in the full protocol. Thank you for the comment. The manuscript has been updated with section explaining sample collection including details about both blood and nasal sampling. Page 11, lines 315-323

10. The Safety Reporting document appears to be largely copied straight from the protocol, so it is not clear what this document adds. There are also issues with the table numbering in this document, and it references the groups 1r4, 2r4, etc from the amended protocol, which are not mentioned anywhere in the main publication. In this document (and the full protocol), it is stated that "the investigator will assess the maximum intensity… based on the investigator's clinical judgement". How is the maximum intensity different from the maximum severity as recorded by the parent/caregiver? This final assessment seems quite subjective and open to bias.

Thank you for your comment. A separate safety reporting document has been created, as the manuscript cannot cover the detailed explanation. This document is cited in the manuscript under the 'Safety Reporting' heading for further reference. Page 13

Caregiver reported maximum severity reflects the highest symptom grade using predefined criteria as defined in the safety reporting document for all solicited AEs, while investigator assessed maximum intensity is a clinical judgment integrating these reports with medical context and event progression which are mostly confined to unsolicited AE/SAEs. Both are recorded separately to ensure transparency and reduce bias.

11. The Laboratory Analysis Plan still has some formatting track changes visible. Corrected and attached.

VERSION 2 - REVIEW

**Reviewer**	**1**
**Name**	**Temple, Beth**
**Affiliation**	**Menzies School of Health Research**
**Date**	**26-Jun-2026**
**COI**	

Thank you for your response to my previous comments and for the amended manuscript. I have still have a few outstanding queries:

1. Protocol version

• You state that “this manuscript describes the original protocol” (line 200) – the manuscript should instead describe the current protocol, especially as the majority of participants will be enrolled after the major amendment.

• There is inconsistency in the reporting of the protocol versions, both within the manuscript and comparing the manuscript with the Amendment History in Appendix A of the latest Protocol

- Line 108 protocol version listed is not the latest – please correct.

- The date for version 2 listed in line 108 doesn’t match the date for version 2 noted in line 193 or the date in the Protocol Appendix A

- The date for version 2 in line 193 is after the date enrolment started, even though this is noted as the “initial approval”

2. Sample size

• Thank you for providing a reference for the 0.67 non-inferiority margin – please could you also include the brief rationale in the text (eg. “in line with the WHO guidelines on clinical evaluation of vaccines”)

• Thank you for your response in relation to my question about the overall conclusion of non-inferiority – from your response it sounds like the trial will not provide an overall conclusion but will report non-inferiority for each serotype separately. In this case, please remove the wording “non-inferiority conclusion” from line 260 and instead state that non-inferiority will be described for each serotype, otherwise the text is misleading.

Other minor points:

• Line 167 Study aims and objectives – please briefly describe the PCV schedules in the different arms here, as the arm numbers are used in Table 1 before any description of which arm is which.

• Needs proof-reading for minor language and grammatical errors

---

VERSION 2 - AUTHOR RESPONSE

Reviewer: 1

1. Protocol version

You state that “this manuscript describes the original protocol” (line 200) – the manuscript should instead describe the current protocol, especially as the majority of participants will be enrolled after the major amendment. – Thank you for this comment.

The manuscript describes the original protocol (V2.0, 18Sep2024) under which 237 participants had been enrolled (40% of the planned sample size) prior to the implementation of the first substantial amendment. The subsequent substantial amendments (3 – 5) were undertaken to accommodate changes to the UK infant immunisation schedule which occurred during study enrolment and are described in the sections 'Protocol amendment affecting PCV schedule' and 'Amendments'. The amendments were approved over a period extending from 24th June 2026 (substantial amendment 2) through to November 25th 2026 (substantial amendment 5). None of the amendments altered the primary endpoint of the study and under any specific version of the protocol a smaller fraction of infants had been enrolled. The protocol incorporating all of these amendments (V5.0, 02OCT2025) is included in Supplementary Document.

Considering the nature and timing of the amendments as detailed above we think that the study is most clearly described and understood (in terms of aims, design, end-points and also the evolution of the amendments) with the original protocol outlined, together with a detailed amendment history, and then reference to the current version of the protocol (V5.0, 02OCT2025) with the amended schedule of vaccines included as a supplementary document referred to clearly from the text.

• There is inconsistency in the reporting of the protocol versions, both within the manuscript and comparing the manuscript with the Amendment History in Appendix A of the latest Protocol
- Line 108 protocol version listed is not the latest – please correct. – This manuscript describes the original protocol (Version 2.0, 18Sep2024), which was in effect when the first participant was enrolled. Major amendments to the study design introduced subsequently are described in the supplementary document entitled "major protocol amendment". In addition, any inconsistencies in the protocol version numbers and dates have been corrected throughout the manuscript.

- The date for version 2 listed in line 108 doesn't match the date for version 2 noted in line 193 or the date in the Protocol Appendix A - Thanks for the comment. This has been corrected in the manuscript.

- The date for version 2 in line 193 is after the date enrolment started, even though this is noted as the "initial approval". Thank you for your comment. The date of approval for (V2.0, 18Sep2024) is 23rd Oct 2024 and enrolment for the trial began on 03rd Dec 2024. This has now been corrected as suggested. Also, in section major protocol amendments two dates are mentioned. The dates with version number are the submission dates while those mentioned later are approval dates for those versions.

2. Sample size

• Thank you for providing a reference for the 0.67 non-inferiority margin – please could you also include the brief rationale in the text (eg. "In line with the WHO guidelines on clinical evaluation of vaccines"). Thank you for your comment. The text has been revised to include this.

• Thank you for your response in relation to my question about the overall conclusion of non-inferiority – from your response it sounds like the trial will not provide an overall conclusion but will report non-inferiority for each serotype separately. In this case, please remove the wording "non-

inferiority conclusion" from line 260 and instead state that non-inferiority will be described for each serotype, otherwise the text is misleading. Thank you for your comment. As per the suggestion, changes have been made and following lines are added in the manuscript – "The overall conclusion regarding non-inferiority will also take the epidemiological data into consideration, as non-inferiority may not be met for all serotypes due to variability."

Other minor points:

• Line 167 Study aims and objectives – please briefly describe the PCV schedules in the different arms here, as the arm numbers are used in Table 1 before any description of which arm is which. - Thank you for the comment. As suggested, this has been added to study aims and objectives section.

• Needs proof-reading for minor language and grammatical errors – Thank you for the comment. A through proof reading has been done and minor language and grammatical mistakes have been corrected throughout the manuscript.
