# Supplementary material for: Open-label, randomised controlled, multicentre study evaluating higher valency pneumococcal vaccines (PCV15/PCV20) compared with PCV13 given in homologous schedules at 3 months and 12 months (‘1+1’ schedule) and 2 months, 4 months and 12 months (‘2+1’ schedule) in infants: protocol
Source: BMJ Open. 2026 Sep 22;16(9):e116062. doi: 10.1136/bmjopen-2025-116062 (PMC13599847; doi:10.1136/bmjopen-2025-116062)
Supplement: Supplementary file 3 [file bmjopen-16-9-s002.docx]

*a*

| Laboratory Analysis Plan | | |
| --- | --- | --- |
| Study title | Evaluation of higher valency pneumococcal vaccines (PCV15/PCV20) compared to PCV13 given in homologous schedules at 3 months and 12 months (“1+1” schedule) and 2 months, 4 months and 12 months (“2+1” schedule) in infants | |
| Study acronym | [PCV] The PCV15/PCV20 Study | For Clinic labels (PCV) |
| Internal Reference Number | OVG2023/10 | For labels (202310) |
| Ethics Reference | REC reference 24/SC/0222 | |
| IRAS project ID | P-1009039 | |

###### Table of Contents

[1	Modification History	4](#_Toc176267989)

[2	List of Abbreviations	4](#_Toc176267990)

[3	Scope of Lab Analysis Plan	4](#_Toc176267991)

[4	Overview of Study	4](#_Toc176267992)

[4.1	Study Overview	4](#_Toc176267993)

[4.2	Endpoints, related analysis methods and their principles	6](#_Toc176267994)

[4.2.1	Primary Objective and Laboratory Endpoints	6](#_Toc176267995)

[4.2.2	Secondary Objectives and Laboratory Endpoints	6](#_Toc176267996)

[4.2.3	Exploratory Objectives and Laboratory Endpoints	7](#_Toc176267997)

[5	Roles, Responsibilities and Time Points	8](#_Toc176267998)

[6	Analytical Process	10](#_Toc176267999)

[6.1	Sample Processing	10](#_Toc176268000)

[6.1.1	Sample Receipt	10](#_Toc176268001)

[6.1.2	Aliquots/volumes and testing lab	11](#_Toc176268002)

[6.1.3	Serum processing (see Figure 2)	12](#_Toc176268003)

[Sample Analysis	14](#_Toc176268004)

[6.2	Equipment and Reagents	15](#_Toc176268005)

[6.2.1	Major equipment	15](#_Toc176268006)

[6.2.2	Materials required for sample processing and storage	15](#_Toc176268007)

[6.2.3	Non-commercial reagent requirements	15](#_Toc176268008)

[6.3	Sample Shipment and Long Term Storage	16](#_Toc176268009)

[6.3.1	Shipping frozen samples to OVG (All study sites)	16](#_Toc176268010)

[6.3.2	Local Sample storage (will vary per site depending on recruitment)	17](#_Toc176268011)

[6.3.3	Sample Shipment and External Storage	17](#_Toc176268012)

[7	Data Management	18](#_Toc176268013)

[8	Quality Control and Quality Assurance	18](#_Toc176268014)

[9	References	18](#_Toc176268015)

[10	Appendix 1 – Document templates	19](#_Toc176268016)

10.1	Appendix 2 – Sample storage in Boxes.......................................................21

# 1 List of Abbreviations

| Abbreviation | full |
| --- | --- |
| SOP | Standard Operating Procedure |
| OVG | Oxford Vaccine Group |
| PCV | Pneumococcal Conjugate Vaccine |
| SBA | Serum Bactericidal Assay |
| MSD | Meso Scale Discovery platform |
| MLF | Mucosal Lining Fluid (SAM strip eluent) |
| MenB | Neisseria meningitidis Serogroup B |
| PT | Pertussis Toxin |
| FHA | Filamentous Haemagglutinin |
| PRN | Pertactin |
| DT | Diphtheria Toxoid |
| TT | Tetanus Toxoid |
| Hib | Haemophilus influenzae Type B |

# 2 Scope of Lab Analysis Plan

This laboratory analysis plan ensures that the PCV15_20 study samples can be processed and analysed safely and accurately according to protocol and local site needs. The schedule of events including visits, vaccinations, blood draws and group sizes are shown in Table 1. Participants will be enrolled for 11 months until 1 month after final vaccination.

# 3 Overview of Study

## 3.1 Study Overview

Pneumococcal disease includes a wide range of infections caused by different types of pneumococcus bacteria (*Streptococcus pneumoniae*). There are over 90 different types of these bacteria, and vaccines have been produced to protect against the types that cause the most disease.

Children who get pneumococcal conjugate vaccinations (PCVs) have direct immunity against vaccine-type (VT) invasive pneumococcal disease (IPD) and indirectly protect the general population by lowering VT carriage.

In 2022, PCV15, which protects against the PCV13 pneumococcal serotypes as well as serotypes 22F and 33F, was licensed for use from 6 weeks of age in a 1+1 schedule in the UK. While PCV20 protects against 20 pneumococcal serotypes and is currently licensed for use in adults. These vaccines are currently not included in the UK national immunisation programme. However, in June 2023, Advisory Committee on Immunization Practices (ACIP) approved the use of 20-valent pneumococcal conjugate vaccine (PCV20) in children in the USA.

Infants currently receive two doses of the PCV13 vaccine at ages 3 and 12 months, following the UK immunization schedule. Given the ongoing problem of serotype replacement this study will compare the immune response of PCV15 and PCV20 given at 3 and 12months as a 1:1 schedule and PCV20 as a 2:1 schedule and determine if these are non-inferior to the current PCV13 vaccine. The study is funded by NISEC and sponsored by University of Oxford. The study is being conducted by Oxford Vaccine Group.

The study will be an open label, randomised controlled, multicentre trial with a sample size of 600 participants being recruited and assigned to four arms (150 participants per arm). Healthy children will be enrolled at approximately 2 months of age (+2 weeks) and participants in arms 1, 2 and 4 will receive 2 doses of either PCV15,20 or 13 at 3 months and 12 months. Arm 3 will be a 2:1 schedule with 2 doses of PCV20 given at 2, 4 and 12 months of age.

Table 1 Schedule of visits, vaccine and sample collection

| **Visit** | **V1** | **V2** | **V3*** | **V3(A)*** | **V4** | **V5** |
| --- | --- | --- | --- | --- | --- | --- |
| **Age(months)** | **2m** | **3m** | **4m** | **5m** | **12m** | **13m** |
| **Blood samples for Arms 1, 2 and 4** | - | - | X  4ml max | - | - | X  6ml max |
| **Blood samples for Arm 3** |  |  |  | X  5ml max |  | X  6ml max |
| **Nasal sample (^§^MLF-SAM) for Arms 1,2+4** |  | X | X |  |  | X |
| **Nasal sample (^§^MLF-SAM) for Arm 3** | X | X |  | X |  | X |
| **Arms 1-4 (routine immunisations)** | 6:1, MenB, Rotavirus | 6:1, Rotavirus | 6:1, MenB |  | MenB, Hib/MenC  MMR |  |
| **Arm 1 (n=150)** | - | PCV15 | - | - | PCV15 | - |
| **Arm 2 (n=150)** | - | PCV20 | - | - | PCV20 | - |
| **Arm 3 (n=150)** | PCV20 | - | PCV20 | - | PCV20 | - |
| **Arm 4 (n=150)** | - | PCV13 | - | - | PCV13 | - |

^§^MLF-SAM = Mucosal lining fluid by SAM strip

*V3/V3A: Ensure that the correct sample visit labels are used when processing V3 (Arms 1,2+4) vs V3A (Arm 3)

## 3.2 Endpoints, related analysis methods and their principles

All Laboratory related Primary, Secondary and Exploratory objectives/endpoints are described below. The analysis plan considers only the laboratory aspects for sample receipt, processing, tracking and storage and shipment, with brief information on the external laboratories performing the assays as per the protocol.

### 3.2.1 Primary Objective and Laboratory Endpoints

| Primary objective | Outcome measure | Timepoint(s) evaluated of this outcome measure if applicable | Method of Assessment/Rational | Testing Laboratory |
| --- | --- | --- | --- | --- |
| To determine if the PCV13 serotype-specific serum IgG concentration is non-inferior in homologous arms 1 & 2 in comparison to the control arm 4 | PCV13 serotype specific serum IgG concentration | At 13months of age  (at least 4 weeks after administration of the last PCV vaccine) | Meso Scale Discovery (MSD) multivalent assay  Gold standard test for anti-pneumococcal IgG responses | Great Ormand Street Hospital (**GOSH**), Institutes for Child Health (Guildford Street, London, WCN 1EH). |

### 3.2.2 Secondary Objectives and Laboratory Endpoints

| Secondary objective | Outcome measure | Timepoint(s) evaluated of this outcome measure if applicable | Method of Assessment/ Rational | Testing Laboratory |
| --- | --- | --- | --- | --- |
| To determine if the PCV13 serotype-specific serum IgG concentrations are non-inferior in arm 3 at 13 months in comparison to the control arm 4 | PCV13 serotype-specific serum IgG concentration | At 13 months of age (at least 4 weeks after administration of the last dose of PCV vaccine) | Meso Scale Discovery (MSD) multivalent assay  Gold standard test for anti-pneumococcal IgG responses | Great Ormand Street Hospital (**GOSH**), Institutes for Child Health (Guildford Street, London, WCN 1EH). |
| To characterise the seven additional PCV20 serotype-specific serum IgG concentrations at 13 months following homologous PCV schedules | PCV20 serotype-specific serum IgG concentration | At 13 months of age (at least 4 weeks after administration of last dose of PCV vaccine). Serotypes 8, 10A, 11A, 12F, 15B, 22F, 33F |  |  |
| To determine safety and reactogenicity of different schedules of PCV15 and PCV20 | Solicited AEs  Unsolicited AEs  SAEs | within 7 days  within 28 days  Across whole study | No lab endpoints | No Lab endpoints |

### 3.2.3 Exploratory Objectives and Laboratory Endpoints

| Exploratory objective | Outcome measure | Timepoint(s) evaluated of this outcome measure if applicable | Method of Assessment/ Rational | Testing Laboratory |
| --- | --- | --- | --- | --- |
| To determine immunogenicity against concomitant vaccines given as part of the infant routine immunisation schedule | IgG antibody GMCs for **PT, FHA in and PRN, DT, TT and Hib. | At 13 months of age  (at least 4 weeks after administration of the last dose of PCV vaccine). | Multiplexed bead assay | **RIVM**,  Centre for Infectious Disease Control (Antonie van Leeuwenhoeklaan 9, 3721 MA Bilthoven, the Netherlands) |
|  | SBA GMT against MenB | At 4 months and 13 months | SBA | Gold standard test for MenB SBA  **UKHSA**  Vaccine Evaluation Unit, Clinical Sciences Building 2, Manchester Royal Infirmary, Oxford Road, Manchester M13 9WL |
| To characterise the PCV20 serotype-specific IgG concentration following one dose of PCV13, PCV15, or PCV20 | PCV20 serotype specific serum IgG concentrations | At 4 months | Meso Scale Discovery (MSD) multivalent assay | Gold standard test for anti-pneumococcal IgG responses:  Great Ormand Street Hospital (**GOSH**), Institutes for Child Health (Guildford Street, London, WCN 1EH). |
| To characterise the PCV20 serotype-specific IgG concentration following two doses of PCV20 (2+1) schedule | PCV20 serotype specific serum IgG concentrations | At 5 months |  |  |
| To determine if vaccine induce IgG response at the mucosa (nasal) and if antibody levels in the nose correlate with levels in blood. | PCV13 serotype specific nasal IgG GMC against some of the PCV serotypes (sample volume depending) | At 13 months of age  (at least 4 weeks after administration of the last dose of PCV vaccine). | TBC | TBC |
| To detect pneumococcal colonisation and serotype present. | Molecular microbiology (fluidic qPCR for detection of lytA, paiB and serotyping) | At 13 months of age  (at least 4 weeks after administration of the last dose of PCV vaccine). | qPCR on MLF | OVG |

**pertussis toxin (PT), filamentous haemagglutinin (FHA) and pertactin (PRN), diphtheria toxoid (DT), tetanus toxoid (TT) and Haemophilus influenzae type b (Hib) capsular polysaccharide, and serum bactericidal activity (SBA) against Neisseria meningitidis serogroup B (MenB)

| Time Point | Expected Start Date | Expected Completion Date |
| --- | --- | --- |
| Pre-enrolment Regulatory submission and approval, site initiation | SIM 19^th^ Sept 2024 | October 2024 |
| Enrolment and study visits | November 2024 | LPLV |
| Sample processing | January 2025 | LPLV |
| Data analysis | March 2026 | May 2026 |

# 4. Analytical Process

## 4.1 Sample Processing

Sample processing required to meet the study objectives are listed below and will be performed according to the listed SOP.

NB: If access to computers/barcode scanner is restricted – please use the paper sample processing log (Appendix 1, Figure 6). This should be a last resort and it is imperative that all information, including the correct aliquot barcode information, are entered into the correct tabs in the electronic inventory (Figure 4) as soon as possible. Samples cannot be shipped from study sites without the electronic inventory being complete.

| Process | Sample | | | SOP Ref. | Agreed deviation from SOP  or  Assay performed by other laboratory |
| --- | --- | --- | --- | --- | --- |
|  | Container | Volume | Frequency |  |  |
| Serum Separation | Gold/ Red Cap Vacutainer   | 4ml at V3  5ml at V3A  6ml at V5 | 2 visits | OVGL030 | No planned deviation from SOP. |
| Mucosal lining fluid (SAM) | SAM strip   | | Arms 1,2+4  = 3 visits  Arm 3  = 4 visits | OVGL030 | No planned deviation from SOP |

### 4.1.1Sample Receipt

1) Upon receipt in the lab, check that the blood form information matches the serum and (or) SAM strip ID number and visit number (especially check V3 vs V3A).
2) For all sites (except Oxford, site number 01) the clinical PID and lab participant ID will match (Figure 1B).
3) Also check sampling date and time are correct. If there are any queries, follow up with the clinical team immediately.
4) Scan in the sample barcode into the sample admin tracker tab in the excel inventory (use paper log if not immediately available, Figure 6).
5) Please leave a comment if sample integrity is affected or there is insufficient volume to process so that there is full sample traceability.

**Figure 1 Example labels and where to use them**

See Appendix 2 for more information on how to store samples in boxes correctly.

### 4.1.2 Aliquots/volumes and testing lab

Table 2 Sample Aliquot information

| Sample | Sample name | Volume (µl) | Aliquot storage and cryobox label | Destination lab |
| --- | --- | --- | --- | --- |
| Blood  -V3 - 4ml  -V3A - 5ml  -V5 - 6ml | Aliq-01 | (400µl) | -80°C – cryobox labelled as shown in Figure 1 | GOSH |
|  | Aliq-02 | (600µl) | -80°C – cryobox labelled as shown in Figure 1 | UKHSA |
|  | Aliq-03 | 50µl |  | RIVM |
|  | RET-01  RET-02  RET-03 | Split remaining volume depending on visit blood volume (max 1.5ml per vial) | -80°C – cryobox labelled as shown in Figure 1 | OVG |
| Mucosal (SAM)  -Arms 1,2+4 =V3, V4, V5  -Arm 3 = V1, V2, V3a, V5 | SAM | Transported to local lab on ice.  Re-label with correct aliquot label and freeze in collection tube | Freeze immediately at -80°C – cryobox labelled as shown in Figure 1 | OVG |

Figure 2: Serum Processing Overview Diagram (see section 6.1.3)

### 4.1.3 Serum processing (see Figure 2)

1) Serum will be allowed to clot for 30 minutes at room temperature and then stored at 2-8°C until processed. Serum must be processed within 24 hours of venepuncture.
2) Serum will be centrifuged at 4°C, 4000xg for 15 minutes
3) Label aliquot tubes as shown in Figure 1D to ensure barcode can be scanned
4) The serum will then be aliquoted into prelabelled cryovials (Table 2). Aliquot volumes accurately with an adjustable pipette (not a pastuer ).
5) Avoid getting red blood cells in the serum aliquot
6) Please leave a comment in the inventory if the sample is haemolysed (red serum).
7) If there is insufficient volume to complete all aliquots, then prioritise as follows.
   a. Aliquot 01 for GOSH
   b. Aliquot 02 for UKHSA
   c. Aliquot 03 for RIVM
   d. Retains – fill sequentially (maximum of 3 retains) – discard any excess serum.
8) Add a comment to the inventory tab for Aliq01 that states which aliquots are missing, e.g. “no retain aliquots”).
9) Scan the correct barcodes into the correct inventory tab. (*If using paper log at time of processing, it is important that all of this information is transferred to the electronic inventory as soon as possible – including accurate, individual barcode information for each aliquot in each tab*).
10) Store the aliquots in the correctly labelled (9x9) cryobox (Figure 1E) at -80°C.
11) Ensure that that the aliquots are stored in the correct locations (e.g. Aliquot 01, Box 1, A1). Boxes should be filled as shown in Figure 1F.
12) A separate cryobox should be created for each aliquot type. Therefore, you will have four separate boxes: Aliquot-01, Aliquot-02, Aliquot-03, Retains 01-03.
13) You will be provided with box labels (place one on top of the lid and one on the front of the box base Figure 1E).
14) All serum samples will be shipped to OVG on request (refer to section 6.3)

**SAM strips**

Figure 3 SAM strip lab processing/storage

1) The SAM strips will be received at a temperature of 2-8°C from the clinic and processed immediately.
2) Label the SAM strip collection tube with the appropriate lab cryovial barcode label, as shown in Figure 3, ensuring that the correct visit number is used.
3) Remove the clinic barcode label.
4) Scan the lab barcode into the excel inventory SAM strip tab
5) Store in cryobox at -80°C using the taller cryobox with 7x7 grid.
6) Ensure the box number and location match the inventory, e.g. box 1, A1, see Figure 1E and Figure 1F box labelling for fill direction)
7) All SAM strips will be shipped to OVG (refer to section 6.3) and extraction of mucosal lining fluid (MLF) from the SAM strips will occur at OVG.

Figure 4 Example of the electronic inventory template

The inventory templates will be placed onto your TEAMs channel. You can save locally as long as it is secure and backed-up. Otherwise, work from the TEAMs channel.

## Sample Analysis

Assays required to meet the study objectives are listed below and will be performed according to the listed SOP. Serum and mucosal assays are established assays at external laboratories.

| Objectiveendpoint | Sample |  | Assay |  | Agreed deviation from SOP  or  Assay performed by other laboratory |
| --- | --- | --- | --- | --- | --- |
|  | Type | Storage | Name | SOP Ref. |  |
| Primary and Secondary Objective:  Serotype specific Serum IgG antibody levels | Serum | -80°C | MSD  Pneumococcal serotypes | Assays conducted at GOSH | GOSH local SOPs |
| Secondary Objective:  SBA for MenB | Serum | -80°C | SBA for MenB | UKHSA | UKHSA local SOPs |
| Exploratory Objective: Routine immunisation IgG Antibody levels | Serum | -80°C | Multiplexed Assay  Hib, TT, DT, PT, FHA, Prn. | RIVM | RIVM local SOPs |
| SBA for MenB | Serum | -80°C | SBA for MenB | UKHSA | UKHSA local SOPs |
| Mucosal IgG responses | MLF | -80°C | TBC  IgG for pneumococcal serotypes | TBC | TBC |
| Detection of pneumococcal colonisation and serotype | MLF | -80°C | Molecular microbiology (fluidic qPCR for detection of lytA, paiB and serotyping) | OVG | Local OVG SOPs |

## 4.2 Equipment and Reagents

Equipment and Reagents required to meet the study objectives are listed below.

### 4.2.1 Major equipment

| Assay | Major equipment required | Material required |
| --- | --- | --- |
| Serum separation | Centrifuge  -80°C Freezer  Barcode scanner with Computer (Excel+word)  Pipettes (ajustable)  P1000, P100  Sterile pipette tips  Microbiological Safety Cabinet class I/II | Labels – pre-printed provided by OVG (for clinic, Lab and cryoboxes) |
| SAM Strips | -80°C Freezer  Barcode scanner with Computer (Excel+word) | Labels - pre-printed provided by OVG (for clinic, Lab and cryoboxes) |

### 4.2.2 Materials required for sample processing and storage

Each site will purchase their own materials subject to requirement. No deviation from the sizes/volumes should be made. It is important that the correct cryovials and cryoboxes are used to ensure the samples will fit into the long-term storage system at OVG at the end of the trial.

| Material | Company | Material required |
| --- | --- | --- |
| Serum cryovials | Sarstedt Ltd | #72.694.006, 2ml, Pack of 1000 |
| *Cryoboxes -serum | Fisher Scientific | 11872473 (W:133mmxL:133mmxH:50mm), pack of 10 |
| Cryobox 9x9 inserts -serum | Fisher Scientific | #11802513 9x9 box inserts (H:25mm), pack of 10 |
| *Cryoboxes (deep for SAM strips) | Fisher Scientific | #11832493 (W:133mmxL:133mmxH:130mm), Pack of 10 |
| Cryobox 7x7 inserts for SAM strips | Fisher Scientific | #12681565 7x7 box inserts (H:65mm), pack of 10 |

### 4.2.3 Non-commercial reagent requirements

Not applicable.

## 4.3 Sample Shipment and Long Term Storage

Shipping & storage of study Serum (Aliq01, Aliq02, Aliq03, Ret 1-3) and SAM strips will be conducted according to OVGL003 Laboratory Shipment of samples and OVGL018 Cold Storage in the OVG Laboratory.

Table 3 Study sites and site numbers

| External sites | External site code |
| --- | --- |
| Bristol Vaccine Centre | 25 |
| University of Nottingham Health Service | 28 |
| St. George's University Hospitals NHS Foundation Trust | 26 |
| University Hospital Southampton NHS Foundation Trust | 02 |
| Alder Hey Children’s Hospital NHS Foundation Trust | 36 |
| Oxford, Centre for Clinical Vaccinology & Tropical Medicine (CCVTM | 01 |

### 4.3.1 Shipping frozen samples to OVG (All study sites)

All samples (serum aliquots and SAM strips) will be shipped to the Oxford Vaccine Group, up on request, and then re-distributed to the external testing labs after consolidation.

F.A.O

Charlie Appleby / Dr Elizabeth Clutterbuck

Oxford Vaccine Group

Centre for Clinical Vaccinology and Tropical Medicine

University of Oxford, Churchill Hospital, Oxford, OX3 7LE
Tel: +44 01865 611321

OVG will arrange shipments in advance, using Davies International and

emails communication will be sent to all sites when shipments to Oxford Vaccine Group are required

Once a shipment request is received each site should do the following checks

1. All aliquots/SAM strips present on site at time of request can be shipped to OVG.
2. Ensure that the electronic inventory matches the box contents exactly
3. Ensure that all samples were processed with the correct lab ID and visit number
4. A shipment receipt (Appendix 1, Figure 5) will be included in the request email, this should be completed with the cryobox numbers and number of aliquots being sent.
5. Return this by email along with an electronic copy of the box inventories
6. All document templates will also be on the site TEAMs channel.
7. Confirmation of courier pick should be emailed (along with any issues that occur)

OVG will confirm receipt of samples and contact the site if there are any issues upon consolidation.

### 4.3.2 Local Sample storage (will vary per site depending on recruitment)

| Assay/ Sample | Study Storage | No. subjects | No. visits | Aliquots/ Sample | Total | Samples/ Box | No. Boxes |
| --- | --- | --- | --- | --- | --- | --- | --- |
| Serum | -80°C Freezer | 600 | All Arms = 2 | 6 | 600*2*6  7200 | 81 | 89 |
| SAM | -80°C Freezer | Arms 1,2+4 = 450  Arm 3 = 150 | Arms 1,2+4 =3  Arm 3 = 4 | 1 | 450x3=1,350  150x4=600 | 49 | 40 |

Freezer and Cryostore space requirements at OVG:

|  | Total No. Boxes | Boxes/ Drawer* | Drawers* |
| --- | --- | --- | --- |
| Cryoboxes in -80 Storage: serum | 89 | 4 | 23 |
| Cryoboxes in -80 Storage: SAM | 40 | 6 | 7 |

*Tower in LN_2_

### 4.3.3 Sample Shipment and External Storage

| Sample | External Laboratory/ Post Study Storage | Shipment Time Point | Expected Shipment/ Storage Date |
| --- | --- | --- | --- |
| Serum Aliquot-01 | GOSH Lab – discarded after use | TBC | TBC |
| Serum Aliquot-02 | UKHSA – discarded after use | TBC | TBC |
| Serum  Aliquot-03 | RIVM – discarded after use | TBC | TBC |
| Serum  RET-01-03 | OVG Archive if Biobank consent | TBC | TBC |
| SAM | OVG Archive | TBC | TBC |

# 5. Data Management

All laboratory information relating to the study will be recorded as listed below to ensure all analyses are complete and performed according to the clinical protocol. Data will be recorded, analysed and stored/archived according to the following SOPs:

OVC 037 Confidentiality and Information Security

OVC 020 Archiving

OVGL 027 Release of Results

Data for the individual assays is captured electronically using equipment as described in Table 4.

Table 4: Data capture

| Assay | Data capture | Data analysis | Data storage |
| --- | --- | --- | --- |
| Serum IgG- GOSH | GOSH - Local systems | Study Stats Team | OVG Secure drives |
| SBA – UKHSA | UKHSA - Local systems | Study Stats Team | OVG Secure drives |
| Hib, DT, TT, PT, FHA, PRN -IgG | RIVM – local systems | Study Stats Team | OVG Secure drives |

# 6. Quality Control and Quality Assurance

The laboratory results will provide a large proportion of the data collected and analysed in this study. Measures taken to ensure the quality of the data are listed below.

Data will be released by external testing laboratories to the Study Stats Team and QA Team.

Sample receipt, storage and shipment will be reviewed according to the study monitoring plan. If additional quality assurance is required, this will be included in the monitoring plan or as part of an internal audit, conducted by the QA manager.

# 7. References

Good Clinical Laboratory Practice (GCLP) A Quality System for Laboratories that undertake the Analysis of Samples from Clinical Trials. BARQA. (**ISBN** 978-1-904610-00-7)

Guidance on the maintenance of regulatory compliance in laboratories that perform the analysis or evaluation of clinical trial samples. MHRA. July 2009.

# 8. Appendix 1 – Document templates

Figure 5 Shipping receipt template

Document templates will be stored on the TEAMs channel. For shipments from OVG to testing labs, remember to update the destination address

Figure 6 Paper sample processing log

Paper logs should only be used of access to barcode scanner/computer is restricted

All information should be transferred to the electronic inventory

# 10.1 Appendix 2 – Sample storage in boxes

Store the matching aliquots in the correctly labelled box that matches the freezer inventory. On the far left of the inventory, the box number and position should match the position in the box that the sample belongs in. On the right-hand side, the” freezer location” column states where in the freezer the sample gets stored. The box names will also match the appropriate name on the inventory.
