# Supplementary material for: Open-label, randomised controlled, multicentre study evaluating higher valency pneumococcal vaccines (PCV15/PCV20) compared with PCV13 given in homologous schedules at 3 months and 12 months (‘1+1’ schedule) and 2 months, 4 months and 12 months (‘2+1’ schedule) in infants: protocol
Source: BMJ Open. 2026 Sep 22;16(9):e116062. doi: 10.1136/bmjopen-2025-116062 (PMC13599847; doi:10.1136/bmjopen-2025-116062)
Supplement: Supplementary file 4 [file bmjopen-16-9-s003.docx]

# 1. SAFETY REPORTING

Safety reporting will be carried out in accordance with the Oxford Vaccine Centre SOP “Safety reporting for CTIMPs” (OVC005).

All adverse events (AEs), including those that result in a participant’s withdrawal from the study, will be followed up until a satisfactory resolution occurs, or until a non-study related causality is assigned (if the participant’s parent/ legal guardian consents to the child being followed up after withdrawal).

## 1.1. Adverse Events Definitions

| Adverse Event (AE) | Any untoward medical occurrence in a participant to whom a medicinal product has been administered, including occurrences which are not necessarily caused by or related to that product. |
| --- | --- |
| Adverse Reaction (AR) | An untoward and unintended response in a participant to an investigational medicinal product which is related to any dose administered to that participant.  The phrase "response to an investigational medicinal product" means that a causal relationship between a trial medication and an AE is at least a reasonable possibility, i.e., the relationship cannot be ruled out.  All cases judged by either the reporting medically qualified professional or the Sponsor as having a reasonable suspected causal relationship to the trial medication qualify as adverse reactions. |
| Serious Adverse Event (SAE) | A serious adverse event is any untoward medical occurrence that:   - results in death - is life-threatening - requires inpatient hospitalisation or prolongation of existing hospitalisation - results in persistent or significant disability/incapacity - consists of a congenital anomaly or birth defect.   Other ‘important medical events’ may also be considered serious if they jeopardise the participant or require an intervention to prevent one of the above consequences.  NOTE: The term "life-threatening" in the definition of "serious" refers to an event in which the participant was at risk of death at the time of the event; it does not refer to an event which hypothetically might have caused death if it were more severe. |
| Serious Adverse Reaction (SAR) | An adverse event that is both serious and, in the opinion of the reporting Investigator, believed with reasonable probability to be due to one of the trial treatments, based on the information provided. |

- NB: to avoid confusion or misunderstanding of the difference between the terms “serious” and “severe”, the following note of clarification is provided: “Severe” is often used to describe intensity of a specific event, which may be of relatively minor medical significance. “Seriousness” is the regulatory definition supplied above.

## 1.2. Safety reporting window

Safety reporting for the study will commence once the participant is consented. It will end following the final study visit (V5).

SAEs resulting in participant withdrawal will be recorded from point of consent (Visit 1).

## 1.3. Assessment of results outside normal parameters as AEs and SAEs

Abnormal clinical findings from medical history or examination will be assessed regarding their clinical significance throughout the study. If an abnormal finding is deemed to be clinically significant, the parent/legal guardian will be informed, and they will be referred to their appropriate GP practice with their permission.

## 1.4. Assessment of Severity

Solicited AEs occurring during the safety window for the trial that are observed by the Investigator or reported by the participant, will be reported on the trial CRF and graded for severity.

## 1.4.1. Solicited events

## 1.4.1.1. Local reactions:

Local solicited reactions will be recorded in the e-Diary / back-up paper diary from day of vaccination until day 6 post vaccination by the parent/legal guardian and will include erythema, induration and swelling (Table 1). Tenderness (pain) at injection site will also be recorded and graded for severity, as shown in Table 3. Parents/legal guardians will be issued with a ruler to measure erythema, induration or swelling which will be categorised according to the table below:

**Table 1: location reactions**

| **Symptoms** | **Grade** | **Definition** |
| --- | --- | --- |
| **Injection site reaction: erythema, induration or swelling** | 0 (none) | No reactions |
|  | 1 (mild) | 1 to ≤25mm |
|  | 2 (moderate) | 26 to ≤50mm |
|  | 3 (severe) | >50mm |

## 1.4.1.2. Systemic reactions

Solicited systemic reactions will be recorded in the e-Diary/ back-up diary card for the day of vaccination and 6 days following vaccination with any PCV vaccine (V1 for arm 3, V2 for arms 1, 2 and 4, V3 for arms1r4, 2r4, 3 and 4r4 and V4 for all arms). These include change in temperature, feeding, lethargy, vomiting, diarrhoea and irritability/fussiness. Parents/legal guardians will grade severity according to Tables 3. Temperature will be recorded daily over this period.

## 1.4.1.3. Temperature

Parents/legal guardians will be asked to record the participant’s axillary temperature at around six hours post vaccine and daily for the 6 days following vaccination with any PCV vaccine (V1 for arm 3, V2 for arms 1, 2 and 4, V3 for arms 1r4, 2r4, 3 and 4r4, and V4 for all arms). The axillary temperature will also be measured if at any time, the parent/legal guardian feels that the participant may have developed a fever. Axillary temperature will be graded as follows:

**Table 2: Temperature Grading**

| **Temperature Grade** | **Temperature definition (degree Celsius)** |
| --- | --- |
| 0 (none) | < 37.6 |
| 1 (mild) | 37.6 – 38.0 |
| 2 (moderate) | 38.1 – 39.0 |
| 3 (severe) | 39.1 – 40.0 |
| 4 (potentially life-threatening) | >40.1 |

**Table 3: Grading of systemic reactions**

|  | **Grading of severity** | | |  |
| --- | --- | --- | --- | --- |
| **Solicited Reactions** | **Mild** | **Moderate** | **Severe** | |
| Tenderness (pain) at the injection site | Minor reaction to touch | Cries / protests on touch | Cries when limb is moved or spontaneously  painful | |
| Change in feeding/eating habit | Feeding / eating less than usual / no effect on normal activity | Feeding / eating less than usual with  an effect on normal activity | Not feeding / eating at all | |
| Drowsiness | Drowsiness easily tolerated | Drowsiness that interferes with normal activity | Drowsiness that prevents normal  activity | |
| Vomiting | 1-2 episodes without interfering with routine | Several episodes & cannot keep any food down | Frequent episodes & not feeding / eating at all | |
| Diarrhoea | Stools are more loose than normal | Frequent runny stools without much solid material | Multiple liquid stools without much solid material | |
| Irritability/fussiness | Crying more than usual / no effect on normal activity | Crying more than usual / interferes with normal activity | Crying that cannot be comforted / prevents normal activity | |

The investigator will assess the maximum intensity that occurred over the duration of the event for all solicited and unsolicited AEs and SAEs. The assessment will be based on the investigator’s clinical judgement. SAEs leading to participant withdrawal will be recorded over the duration of the study.

**Table 4 for assessing the relationship of vaccine administration to an AE**

| 0 | No relationship | No temporal relationship to study product and alternate aetiology (clinical state, environmental or other interventions) and  does not follow known pattern of response to study product |
| --- | --- | --- |
| 1 | Unlikely | Unlikely temporal relationship to study product and alternate aetiology likely (clinical state, environmental or other interventions) and does not follow known typical or plausible pattern of response to study product |
| 2 | Possible | Reasonable temporal relationship to study product or event not readily produced by clinical state, environmental or other interventions or similar pattern of response to that seen with other vaccines |
| 3 | Probable | Reasonable temporal relationship to study product and  event not readily produced by clinical state, environment, or other interventions or known pattern of response seen with other vaccines |
| 4 | Definite | Reasonable temporal relationship to study product ***and***  event not readily produced by clinical state, environment, or other interventions ***and***  known pattern of response seen with other vaccines |

AEs graded in categories 2 to 4 in the above scheme are regarded as having a reasonable suspected causal relationship to the administered vaccine.

## 1.5. Procedures for recording adverse events

## 1.5.1. Reporting of solicited AEs

Participants will be asked to record local and systemic AEs (solicited AEs) for the day of vaccination and 6 days following vaccination with any PCV vaccine (V1 for arm 3, V2 for arms 1, 2 and 4, V3 for arms 1r4, 2r4, 3 and 4r4, and V4 for all arms) (and longer if symptoms persist at day 7, until resolution or stabilisation) in the electronic/paper diary. Temperature will be recorded on the day of vaccination and for 6 days following receiving a dose of any PCV vaccine (V1 for arm 3, V2 for arms 1, 2 and 4, V3 for arms 1r4, 2r4, 3 and 4r4, and V4 for all arms).

## 1.5.2. Reporting of unsolicited AEs

All local and systemic unsolicited AEs occurring in the 28 days following receiving any PCV vaccine (V1 for arm 3, V2 for arms 1, 2 and 4, V3 for arms 1r4, 2r4, 3 and 4r4, and V4 for all arms) observed by the Investigator or reported by the participant (regardless of whether attributed to study medication) will be recorded in the CRF. Serious adverse events occurring at any time over the duration of the study (*i.e.,* from enrolment, whilst the individual participant remains in the study) will also be recorded. The record should include the following: description; date of onset and end date; severity; assessment of relatedness to trial medication.

The severity of events will be assessed on the following scale: 0=none, 1=mild, 2=moderate, 3=severe. Guidelines for scoring of solicited local and systemic reactions are detailed above in Tables 1, 2, and 3.

AEs considered related to the trial medication, as judged by a medically qualified investigator or the Sponsor, will be followed either until resolution or the event is considered stable.

It will be left to the Investigator’s clinical judgment to decide whether an AE is of sufficient severity to require the participant’s removal from treatment. The parents/legal guardians of participants may also voluntarily withdraw from treatment due to what they perceive as an intolerable AE. If either of these occurs, the participant must undergo an end of trial assessment and be given appropriate care under medical supervision until symptoms cease, or the condition becomes stable.

## 1.6. Reporting procedures for Serious adverse events:

All SAEs will be reported to the Investigators by study team members, who will complete the electronic SAE reporting form. Alternatively, a backup paper reporting form will be available; if this is used, it should be sent by email to the Chief Investigator (as the Sponsor’s representative). This will be done immediately and at the latest within 24 hours of the site study team becoming aware of the event. The SAE reporter should ensure, and document, that receipt of the form is acknowledged.

The CI, or a delegate within OVG, will perform an initial check of the report and request any additional information. Additional and further requested information (follow-up or corrections to the original case) will be detailed on a new electronic SAE reporting form; alternatively, the backup paper reporting form may be completed and emailed to the CI (or delegate within OVG).

## 1.7. Expectedness

Expectedness will be determined according to the approved Reference Safety Information (RSI) listed in Section 4.8 (undesirable effects) of the Summary of Product Characteristics. The local PI will assess relatedness, but the CI or delegate will assess expectedness (as delegated by the Sponsor to the CI). The SmPCs relevant to this study are listed in Table 5.

**Table 5: Summary of Product Characteristics**

Prevenar 13 suspension for injection^1^

Prevenar 20 suspension for injection in pre-filled syringe^2^

Vaxneuvance suspension for injection in pre-filled syringe^3^

Vaxelis suspension for injection in pre-filled syringe^8^

Infanrix hexa, Powder and suspension for suspension for injection^4^

Bexsero suspension for injection in pre-filled syringe^5^

Rotarix oral suspension in pre-filled oral applicator^6^

M-M-RvaxPro® powder and solvent for suspension for injection^9^

Priorix - Powder and solvent for solution for injection in a pre-filled syringe^7^

ProQuad powder and solvent for suspension for injection in a pre-filled syringe^10^

Priorix-Tetra powder and solvent for solution for injection in pre-filled syringe^11^

## 1.8. SUSAR reporting

All SUSARs will be reported by the sponsor delegate to the relevant Competent Authority and to the REC and other parties, as applicable. For fatal and life-threatening SUSARS, this will be done no later than 7 calendar days after the Sponsor or delegate is first aware of the reaction. Any additional relevant information will be reported within 8 calendar days of the initial report. All other SUSARs will be reported within 15 calendar days.

## 1.9. Development Safety Update Reports

The Oxford Vaccine Group will submit DSURs for following vaccines: PCV13, PCV15 and PCV20 once a year throughout the clinical trial, or on request to the Competent Authority (MHRA in the UK), Ethics Committee, HRA (where required), Host NHS Trust and Sponsor.

# 2. REFERENCES

1. emc. Prevenar 13 - Summary of product characteristics. 12 Oct 2021.<https://www.medicines.org.uk/emc/product/453>
2. EMA. Prevenar 20 (previously Apexxnar) - Summary of product characteristics. 24 Sept 2024 <https://www.medicines.org.uk/emc/product/13461>
3. emc. Vaxneuvance - Summary of product characteristics. 04 July 2023.

<https://www.medicines.org.uk/emc/product/13754>

4. emc. Infanrix hexa - Summary of product characteristics. 04 Sep 2023.

<https://www.medicines.org.uk/emc/product/2586>

5. emc. Bexsero Meningococcal- Summary of product characteristics. 01 Aug 2023.

<https://www.medicines.org.uk/emc/product/5168>

6. emc. Rotarix - Summary of product characteristics. 31 Oct 2022.

<https://www.medicines.org.uk/emc/product/2739>

7. emc. Priorix - Summary of product characteristics. 20 May 2022.

<https://www.medicines.org.uk/emc/product/1159>

8. emc. Vaxelis - Summary of product characteristics. 25 January 2023. <https://www.medicines.org.uk/emc/product/12264/smpc#gref>.
9. emc. M-M-RvaxPro - Summary of product characteristics. 26 July 2022. <https://www.medicines.org.uk/emc/product/6307/smpc#gref>.
10. emc. ProQuad – Summary of product characteristics. 01 October 2025. https://www.medicines.org.uk/emc/product/101444/smpc
11. 20. emc. Priorix Tetra – Summary of product characteristics. 07 April 2025. https://www.medicines.org.uk/emc/product/2586/smpc
