# Supplementary material for: Open-label, randomised controlled, multicentre study evaluating higher valency pneumococcal vaccines (PCV15/PCV20) compared with PCV13 given in homologous schedules at 3 months and 12 months (‘1+1’ schedule) and 2 months, 4 months and 12 months (‘2+1’ schedule) in infants: protocol
Source: BMJ Open. 2026 Sep 22;16(9):e116062. doi: 10.1136/bmjopen-2025-116062 (PMC13599847; doi:10.1136/bmjopen-2025-116062)
Supplement: Supplementary file 5 [file bmjopen-16-9-s004.docx]

Oxford Vaccine Group

University of Oxford

Centre for Clinical Vaccinology and Tropical Medicine,

Churchill Hospital, Headington, Oxford OX3 7LE

Tel: 01865 611400 [info@ovg.ox.ac.uk](mailto:info@ovg.ox.ac.uk) www.ovg.ox.ac.uk

Participant Number: PCV15-20 -_ _ - _ _ _ _

Parent/Legal Guardian Informed Consent Form

The PCV15/PCV20 Study

Child’s first name and surname: _____________________________ Child’s Initials: ___ ___

*If you agree, please initial box*

| 1 | I confirm that I have read the Study information booklet ‘The PCV 15/ 20 Vaccine Study`, version __ dated _ _ - _ _ _ - _ _ _ _. I have had the opportunity to consider the information, ask questions and have had these answered satisfactorily. |  | |
| --- | --- | --- | --- |
| 2 | I understand that my child’s participation is voluntary and that I am free to withdraw my child at any time without giving any reason, without their medical care or legal rights being affected. |  | |
| 3 | I understand that relevant sections of my child’s medical notes and data collected during the study may be looked at by individuals from the Oxford Vaccine Group, the University of Oxford, from regulatory authorities where it is relevant to my child taking part in this research. I give permission for these individuals to have access to my child’s records during the duration of the study. |  | |
| 4 | I agree for the study team to give my child the vaccines as described in the Study information booklet. |  | |
| 5 | I agree that my child will donate blood and nasal samples. I consider these samples a gift to the University of Oxford, and I understand that my child will not gain any direct personal or financial benefit from them. |  | |
| 6 | I agree to my child’s General Practitioner and Health Visitor team being informed of my child’s participation in the study. |  | |
| 7 | I agree to my child’s General Practitioner and/or other treating doctors and/or any other healthcare provider service being approached for additional information regarding medical and vaccination history and study staff to access my child’s NHS medical records if required. |  | |
| 8 | I understand and agree that my child’s data and samples will be stored and used as outlined in the participant information sheet and this consent form. |  | |
| 9 | I understand and agree that the Sponsor Data Management and IT Team will be able to view my email address. I understand it will be held securely on a server at the University of Oxford. |  | |
| 10 | **I agree for my child to take part in this study.** |  | |
| **Optional:** | | *Initial:* | |
| 11 | *I agree to be contacted about ethically approved research studies for which my child may be suitable. I understand that agreeing to be contacted does not oblige my child to participate in any further studies.* | Yes | No |
|  |  |  |  |
| 12 | *I agree to being contacted about studies in the future that are related to this study, and I understand that I would be under no obligation to take part in the future.* |  |  |

Participant Number: PCV15-20 -_ _ - _ _ _ _

Name (PRINT NAME): __________________________________________________________________

Relationship to child (parent or legal guardian only): _________________________________________

Signature: _______________________________________________ Date: __ __ /__ __ __/__ __ __ __

(DD/MMM/YYYY)

Study Nurse/Doctor’s name (PRINT NAME): _____________________________________________________

Signature: _______________________________________________ Date: __ __ /__ __ __/__ __ __ __

(DD/MMM/YYYY)

*Original to be retained at site in the case report form (CRF); second copy to be given to the parent/guardian.*
