# Supplementary material for: Open-label, randomised controlled, multicentre study evaluating higher valency pneumococcal vaccines (PCV15/PCV20) compared with PCV13 given in homologous schedules at 3 months and 12 months (‘1+1’ schedule) and 2 months, 4 months and 12 months (‘2+1’ schedule) in infants: protocol
Source: BMJ Open. 2026 Sep 22;16(9):e116062. doi: 10.1136/bmjopen-2025-116062 (PMC13599847; doi:10.1136/bmjopen-2025-116062)
Supplement: Supplementary file 6 [file bmjopen-16-9-s005.docx]

**Study Title: Evaluation of higher valency pneumococcal vaccines (PCV15/PCV20) compared to PCV13 given in homologous schedules at 3/4 months and 12 months (“1+1” schedule) and 2 months, 4 months and 12 months (“2+1” schedule) in infants**

**Short Title:** The PCV15/PCV20 Study

**Ethics Reference:**	**24/SC/0222**

**OVG reference:**	**OVG2023/10**

**IRAS Project ID:	P-1009039**

**Date and Version No**:	02 Oct 2025 Version 5.0

**Chief Investigator:**	**Dr. Dominic Kelly**

**Chief Investigator Signature:**	**……………………………………………..**

**Sponsor:	University of Oxford**

**Funder:	National Immunization Schedule Evaluation Consortium (NISEC)**

**Trial Statistician:**	**Prof. Xinxue Liu**

**Statistician Signature:**	**………………………………………………**

**Confidentiality Statement**

This document contains confidential information that must not be disclosed to anyone other than the Sponsor, the Investigator Team, HRA, host organisation, and members of the Research Ethics Committee and Regulatory Authorities unless authorised to do so.

**Trial Title: Evaluation of higher valency pneumococcal vaccines (PCV15/PCV20) compared to PCV13 given in homologous schedules at 3/4 months and 12 months (“1+1” schedule) and 2 months, 4 months and 12 months (“2+1” schedule) in infants**

**Protocol Date and Version No:**	02 Oct 2025 Version 5.0

**Protocol signature page**

The undersigned has read and understood the trial protocol detailed above and agrees to conduct the trial in compliance with the protocol.

| **Principal Investigator Dr. Dominic Kelly** |  | **Signature** |  | **Site name or ID number** |  | **Date** |
| --- | --- | --- | --- | --- | --- | --- |

Table of Contents

[1.	KEY TRIAL CONTACTS	9](#_Toc209618312)

[2.	SYNOPSIS	11](#_Toc209618313)

[3.	ABBREVIATION	14](#_Toc209618314)

[4.	BACKGROUND AND RATIONALE	16](#_Toc209618315)

[5.	OBJECTIVE AND OUTCOMES MEASURES	19](#_Toc209618316)

[6.	TRIAL DESIGN	21](#_Toc209618317)

[7.	PARTICIPANTS IDENTIFICATION	24](#_Toc209618318)

[7.1.	Trial Participants	25](#_Toc209618319)

[7.2.	Inclusion Criteria	25](#_Toc209618320)

[7.3.	Exclusion Criteria	25](#_Toc209618321)

[7.4.	Temporary Exclusion Criteria	26](#_Toc209618322)

[8. TRIAL PROCEDURE	26](#_Toc209618323)

[8.1	Recruitment	26](#_Toc209618324)

[8.2	Screening and Eligibility Assessment	27](#_Toc209618325)

[8.3	Informed Consent	28](#_Toc209618326)

[8.4	Randomisation procedure	29](#_Toc209618327)

[9	STUDY VISITS	29](#_Toc209618328)

[9.1	Visit 1 – Enrolment and vaccination at 8 – 10 Weeks of age	29](#_Toc209618329)

[9.2	Visit 2 – Vaccination and Nasal sampling visit at 28-42 days post V1:	31](#_Toc209618330)

[9.3	Visit 3 – Vaccination and sample collection at 28-42 days post V2.	32](#_Toc209618331)

[9.4	Visit 3(A) – Sample collection at 28-42 days following V3 for arms 1r4, 2r4, 3 and 4r4 only.	33](#_Toc209618332)

[9.5	Visit 4 – Vaccination visits at 12 months (+ 14 days) of age	34](#_Toc209618333)

[9.6	Visit 5 - Blood and nasal sample collection visit 28-42 days following V4.	35](#_Toc209618334)

[9.7	Additional Visit – MenB vaccination only, between Visit 2 and Visit 3	36](#_Toc209618335)

[9.8	Blood sampling	36](#_Toc209618336)

[9.9	Mucosal Lining fluid (MLF) sampling	37](#_Toc209618337)

[9.10	Recording symptoms: e-Diary	37](#_Toc209618338)

[9.11	Clinical samples for Biobank storage	38](#_Toc209618339)

[9.12	Definition of enrolment	38](#_Toc209618340)

[9.13	Early Discontinuation or withdrawal of participants	38](#_Toc209618341)

[9.14	Definition of end of trial	39](#_Toc209618342)

[10	TRIAL INTERVENTIONS	39](#_Toc209618343)

[10.1	Investigational Medical Products (IMPs) Description:	39](#_Toc209618344)

[10.1.1	PCV13 (PREVENAR13®):	39](#_Toc209618345)

[10.1.2	PCV15 (VAXNEUVANCE^TM^)	39](#_Toc209618346)

[10.1.3	PCV20 (APEXXNAR/PREVENAR20®))	40](#_Toc209618347)

[10.2	Storage of IMP	40](#_Toc209618348)

[10.3	Compliance with trial treatment	40](#_Toc209618349)

[10.4	Accountability for IMPs	40](#_Toc209618350)

[10.5	Concomitant medication	40](#_Toc209618351)

[10.6	Other Treatments (non-IMPs):	41](#_Toc209618352)

[10.7	Storage of non-IMP	41](#_Toc209618353)

[10.8	Accountability for non-IMPs	42](#_Toc209618354)

[10.9	Post-trial treatment	42](#_Toc209618355)

[10.10	Other Interventions	42](#_Toc209618356)

[11	SAFETY REPORTING	42](#_Toc209618357)

[11.1	Adverse Events Definitions	42](#_Toc209618358)

[11.2	Safety reporting window	44](#_Toc209618359)

[11.3	Assessment of results outside normal parameters as AEs and SAEs	44](#_Toc209618360)

[11.4	Clinical	44](#_Toc209618361)

[11.5	Assessment of Severity	44](#_Toc209618362)

[11.5.1	Solicited events	44](#_Toc209618363)

[11.5.2	Local reactions:	44](#_Toc209618364)

[11.5.3	Systemic reactions	45](#_Toc209618365)

[11.5.4	Temperature	45](#_Toc209618366)

[11.6	Procedures for recording adverse events	48](#_Toc209618367)

[11.6.1	Reporting of solicited AEs	48](#_Toc209618368)

[11.6.2	Reporting of unsolicited AEs	48](#_Toc209618369)

[11.7	Reporting procedures for Serious adverse events:	48](#_Toc209618370)

[11.8	Expectedness	49](#_Toc209618371)

[Prevenar 13 suspension for injection^1^	49](#_Toc209618372)

[Prevenar 20 suspension for injection in pre-filled syringe^2^	49](#_Toc209618373)

[Infanrix hexa, Powder and suspension for suspension for injection^4^	49](#_Toc209618374)

[11.9	SUSAR reporting	50](#_Toc209618375)

[11.10	Development Safety Update Reports	50](#_Toc209618376)

[12	STATISTICS	50](#_Toc209618377)

[12.1	Sample size	50](#_Toc209618378)

[12.2	Description of statistical methods	51](#_Toc209618379)

[12.3	Missing data	51](#_Toc209618380)

[12.4	Interim analyses	51](#_Toc209618381)

[13	DATA MANAGEMENT	51](#_Toc209618382)

[13.1	Source data	51](#_Toc209618383)

[13.2	Access to data	52](#_Toc209618384)

[13.3	Data Recording and Record Keeping	52](#_Toc209618385)

[14	QUALITY ASSURANCE PROCEDURES	53](#_Toc209618386)

[14.1	Monitoring	53](#_Toc209618387)

[15	PROTOCOL DEVIATION	54](#_Toc209618388)

[16	SERIOUS BREACHES	54](#_Toc209618389)

[17	ETHICAL AND REGULATORY CONSIDERATIONS	54](#_Toc209618390)

[17.1	Declaration of Helsinki	54](#_Toc209618391)

[17.2	Guidelines of Good Clinical Practice	54](#_Toc209618392)

[17.3	Approvals	54](#_Toc209618393)

[17.4	Other Ethical Consideration	55](#_Toc209618394)

[17.5	Reporting	55](#_Toc209618395)

[17.6	Participant confidentiality	55](#_Toc209618396)

[17.7	Expense and benefits	55](#_Toc209618397)

[18	FINANCE AND INSURANCE	56](#_Toc209618398)

[18.1	Funding	56](#_Toc209618399)

[18.2	Insurance	56](#_Toc209618400)

[19	PUBLICATION POLICY	56](#_Toc209618401)

[20	ARCHIVING	56](#_Toc209618402)

[21	SCHEDULE OF EVENTS	57](#_Toc209618403)

[22	REFERENCES	59](#_Toc209618404)

[APPENDIX A: AMENDMENT HISTORY	62](#_Toc209618405)

# 1. KEY TRIAL CONTACTS

| **Chief Investigator** | Dr Dominic F Kelly.  Centre for Clinical Vaccinology & Tropical Medicine (CCVTM), Churchill Hospital, Oxford OX3 7LE, United Kingdom  0044 (0)1865 611400  Email : dominic.kelly@paediatrics.ox.ac.uk |
| --- | --- |
| **Sponsor** | University of Oxford  Research Governance, Ethics & Assurance (RGEA)  1st Floor, Boundary Brook House, Churchill Drive, Headington, Oxford OX3 7GB, United Kingdom. |
| **Funder(s)** | National Immunization Schedule Evaluation Consortium (NISEC). |
| **Statistician** | Professor Xinxue Liu  Lead Trial Statistician  Oxford Vaccine Group  University of Oxford  Centre for Clinical Vaccinology & Tropical Medicine (CCVTM)  Churchill Hospital, Oxford OX3 7LE, United Kingdom  xinxue.liu@paediatrics.ox.ac.uk |
| **Investigators** | Professor Maheshi Ramasamy  Centre for Clinical Vaccinology & Tropical Medicine (CCVTM),  Churchill Hospital,  Oxford OX3 7LE, United Kingdom  Dr Marion Roderick  Bristol Vaccine Centre,  L6 Education & Research Centre,  Upper Maudlin Street,  Bristol, BS2 8AE  Dr David Turner  The University of Nottingham Health Service  Cripps Health Centre,  University Park  Nottingham, NG7 2QW  Prof. Paul Heath  St. George's University Hospitals NHS Foundation Trust and  St. George’s Vaccine Institute (SGVI)  Centre for Neonatal and Paediatric Infection Diseases,  St. George’s University of London  Cranmer Terrace,  London, SW17 0RE    Dr Daniel Owens  University Hospital Southampton NHS Foundation Trust  Southampton General Hospital  Tremona Road  Southampton, SO16 6YD  Dr Dan Hawcutt  Alder Hey Children’s Hospital NHS Foundation Trust  Eaton Road,  Liverpool, L12 2AP  Prof. David Goldblatt  Great Ormond Street Institute of Child Health  University College London  30 Guilford Street London, WC1N 1EH |

# 2. SYNOPSIS

| **Trial Title** | **Evaluation of higher valency pneumococcal vaccines (PCV15/PCV20) compared to PCV13 given in homologous schedules at 3/4 months and 12 months (“1+1” schedule) and 2 months, 4 months and 12 months (“2+1” schedule) in infants** | |
| --- | --- | --- |
| **Clinical Phase** | Phase IV: Post licensure | |
| **Trial Design** | Open label, randomised, non-inferiority, comparative study | |
| **Trial Participants** | Healthy infant population enrolled at 2 months of age | |
| **Planned Sample Size** | N=600  Arm 1 and 1r4: PCV15 at 3/4, 12 months (homologous schedule); N=150  Arm 2 and 2r4: PCV20 at 3/4, 12 months (homologous schedule); N=150  Arm 3: PCV20 at 2, 4 and 12 months; N=150  Arm 4 and 4r4: PCV13 at 3/4, 12 months (control schedule); N=150 | |
| **Treatment Duration** | 11 months | |
| **Follow Up Duration** | 1 month following final vaccination | |
| **Planned Trial Period** | 30 months | |
|  | **Objectives** | **Outcome Measures** |
| **Primary** | To determine if the PCV13 serotype-specific serum IgG concentrations is non-inferior in homologous arms 1/1r4 & 2/2r4 at 13 months in comparison to the control arm 4/4r4 | PCV13 serotype-specific serum IgG concentration at 13 months |
| **Secondary** | To determine if the PCV13 serotype-specific serum IgG concentrations is non-inferior in arm 3 at 13 months in comparison to the control arm 4/4r4 | PCV13 serotype-specific serum IgG concentration at 13 months |
|  | To characterise the seven additional PCV20 serotype-specific serum IgG concentrations at 13 months following homologous PCV schedules in arms 1/1r4, 2/2r4 and 3 in comparison to the control arm 4/4r4 | PCV20 serotype-specific serum IgG concentrations (Serotypes 8, 10A, 11A, 12F, 15B, 22F and 33F) at 13 months |
|  | To determine the safety and reactogenicity of different schedules with PCV15 and PCV20 | Solicited AEs within 7 days;  Unsolicited AEs within 28 days;  SAE across the whole study period. |
| **Exploratory** | To determine immunogenicity against concomitant vaccines given as part of the infant routine immunisation schedule | IgG antibody GMCs at 13 months concentrations against pertussis antigens (pertussis toxin, filamentous hemagglutinin, and pertactin), diphtheria toxoid, tetanus toxin and Hib capsular polysaccharide;  SBA GMTs against MenB at 4/5 months (at least 4 weeks after second dose of MenB vaccine) and 13 months. |
|  | To characterise the PCV20 serotype-specific serum IgG concentrations at 4/5 months following one dose of PCV 13, PCV15 or PCV20 | PCV20 serotype-specific serum IgG concentration at 4/5 months |
|  | To characterise the PCV20 serotype-specific serum IgG concentrations at 5 months following two doses of PCV20 (2+1) schedule. | PCV20 serotype-specific serum IgG concentration at 5 months |
|  | To determine if vaccines induce IgG  responses at the mucosa (nasal) and if  antibody levels in the nose correlate with levels in blood | PCV13 serotype-specific nasal IgG  GMC at 13 months against some of the PCV serotypes (sample volume depending) |
|  | To detect pneumococcal colonisation and serotypes present | Molecular biology methods to detect Spn and serotype. |
|  | To compare immunological responses to PCV for infants given PCV vaccines at 4 and 12 months of age to infants given PCV vaccines at 3 and 12 months of age. | PCV serotype-specific serum IgG concentrations at 1 month post first dose and 1 month post second dose |
| **Investigational**  **Medicinal Products** | **PREVENAR13**® (PCV13; Pfizer Limited): a pneumococcal conjugate vaccine that contains 13 different pneumococcal serotypes (1, 3, 4, 5, 6A, 6B, 7F, 9V, 14, 18C, 19A, 19F and 23F)  **VAXNEUVANCE™** (PCV15; Merck): containing purified capsular polysaccharides from Streptococcus pneumoniae serotypes 1, 3, 4, 5, 6A, 6B, 7F, 9V, 14, 18C, 19A, 19F, 22F, 23F, and 33F individually conjugated to CRM197 toxoid with alum adjuvant  **APEXXNAR/PREVENAR 20®** (PCV20 Pfizer) containing purified capsular polysaccharides from Streptococcus pneumoniae serotypes 1, 3, 4, 5, 6A, 6B, 7F, 8, 9V, 10A, 11A, 12F, 14, 15B, 18C, 19A, 19F, 22F, 23F, and 33F individually conjugated to CRM197 toxoid with alum adjuvant (Prevenar 20 (formerly Apexxnar) and Apexxnar are the same vaccines with different names for different regions). | |
| **Non-Investigational Medicinal Products** | **Infanrix Hexa**® (DTaP-IPV-Hib-Hep B; GSK): diphtheria (D), tetanus (T), pertussis (acellular component) (Pa), hepatitis B (rDNA) (HBV), poliomyelitis (inactivated (IPV) and Haemophilus influenza type b (Hib) conjugated vaccine.  **Vaxelis**® (DTaP-IPV-Hib-Hep B; Sanofi): Diphtheria, tetanus, pertussis (acellular, component), hepatitis B (rDNA), poliomyelitis (inactivated), and Haemophilus type b conjugate vaccine (adsorbed).  **Bexsero®** (MenB; Novartis): a meningococcal serogroup B vaccine adsorbed rDNA vaccine.  **Rotarix®** (Rotavirus; GSK): Human rotavirus RIX4414 strain (live, attenuated).  **ProQuad®** (MMRV; MSD): Measles, mumps, rubella and varicella vaccine (live, attenuated)  **Priorix Tetra®** (MMRV; GSK): Measles, mumps, rubella and varicella vaccine (live, attenuated)  **MMRVAXPRO®** (MMR; MSD): contains live attenuated (weakened) measles, mumps and rubella viruses.  **Priorix®** (MMR; GSK): live attenuated vaccine that protects against measles, mumps and rubella. | |

# 3. ABBREVIATION

| AE | Adverse Event |
| --- | --- |
| CHIS | Child Health Information Service |
| CI | Chief Investigator |
| CRF | Case Report Form |
| CRM_197_ | Cross-reacting material 197 |
| CTIMP | Clinical Trial of an Investigational Product |
| CTM | Clinical Trial Manager |
| DSUR | Development Safety update Report |
| DTaP | Diphtheria, tetanus, acellular pertussis vaccine |
| GCP | Good Clinical Practice |
| GDPR | General Data Protection Regulation |
| GMTs | Geometric Mean Titre |
| GMC | Geometric mean concentrations |
| HepB | Hepatitis B |
| Hib | Haemophilus Influenza type B |
| HRA | Health Research Authority |
| IgG | Immunoglobulin G |
| IPD | Invasive pneumococcal disease |
| IPV | Inactivated polio vaccine |
| JCVI | Joint committee on Vaccination and Immunization |
| MenB | Group B Meningococcus |
| MenC | Group C Meningococcus |
| MHRA | Medicine and Healthcare products Regulatory Agency |
| MMR | Measles, Mumps and Rubella vaccine |
| MMRV | Measles, Mumps, Rubella and Varicella vaccine |
| OVG | Oxford Vaccine Group |
| PCV | Pneumococcal conjugate vaccines |
| PIS | Patient Information Sheet |
| qPCR | Quantitative polymerase chain reaction |
| REC | Research Ethics Committee |
| SAE | Serious Adverse Event |
| SBA | Serum Bactericidal Antibody (hSBA = using human complement; rSBA using rabbit complement) |
| SUSAR | Suspected Unexpected Serious Adverse Reaction |
| VT | Vaccine Type |

# 4. BACKGROUND AND RATIONALE

Pneumococcal disease includes a wide range of infections caused by different types of pneumococcus bacteria (*Streptococcus pneumoniae*). There are over 90 different types of these bacteria, and vaccines have been produced to protect against the types that cause the most disease.

Children who get pneumococcal conjugate vaccinations (PCVs) have direct immunity against vaccine-type (VT) invasive pneumococcal disease (IPD) and indirectly protect the general population by lowering VT carriage.

Additionally, with the adoption of childhood PCV programs, pneumococcal conjugate vaccination (PCV) impact studies have noted a significant decrease in the incidence of invasive pneumococcal disease (IPD). For example, the incidence of invasive pneumococcal disease in children under 2 years of age due to one of the 7 serotypes covered by PCV7 was only 0.38 per 100,000 in the 2012/13 epidemiological year compared with a pre-PCV7 incidence of 39·05 per 100 000, a 99% reduction (Goldblatt et al., 2018)^14^. Following the introduction of PCV13 in the UK, invasive pneumococcal disease (IPD) due to the additional 6 serotypes covered by PCV13 already shows a dramatic decline in incidence in children under 5 years of age from 54% (2008 – 2010) to 15% (2013 – 2014) (Tin Tin Htar et al., 2015)^15^.

Over 38,000 cases of invasive pneumococcal disease (IPD), including sepsis and meningitis, are thought to have been prevented in the UK because of the 7-valent pneumococcal protein-polysaccharide conjugate vaccine's introduction into the regular infant immunization schedule in 2006 and the 13-valent vaccine's introduction in 2010. (Ladhani et al., 2018)^13^ However, while disease caused by many pneumococcal serotypes included in PCV13 has decreased, disease caused by pneumococcal serotypes (22F, 12F, 33F, 24F, 15C, 15B, 23B, 10A, and 38) **(**Méroc et al., 2023)^9^ not included in this vaccine has grown, decreasing the overall impact of the immunization program.

Furthermore, a study conducted in 2022 in Belgium included that due to serotype replacement following the introduction of PCV7 and PCV13, a considerable proportion of pneumococcal disease is currently caused by PCV20-serotypes. PCV20 vaccine has the potential of preventing more pneumococcal disease in children and the adult population at risk than the existing conjugate vaccines (Janssens et al., 2022).**^10^**A phase 2 study, which was reported in a September 2021 article, assessed the immunogenicity, safety, and tolerability of PCV20 in healthy US infants who received a 4-dose series of PCV20 or PCV13 at 2, 4, 6, and 12 months of age (3+1 schedule). The findings showed that PCV20 administration was well tolerated in infants, had a safety profile comparable to PCV13, and produced strong serotype-specific immune responses.^18^

In 2022, PCV15, which protects against the PCV13 pneumococcal serotypes as well as serotypes 22F and 33F, was licensed for use from 6 weeks of age. The JCVI considered the use of PCV15 in a 1+1 schedule and agreed that the current evidence showed it could be used in a 1+1 schedule.^11^ While PCV20 protects against 20 pneumococcal serotypes and is currently licensed for use in adults. These vaccines are currently not included in the UK national immunisation programme.

In response to above, JCVI approved the use of PCV15 in infant immunization schedule in the UK. However, in June 2023, Advisory Committee on Immunization Practices (ACIP) approved the use of 20-valent pneumococcal conjugate vaccine (PCV20) in children in the USA. ^12^

The use of PCV20 (**APEXXNAR/PREVENAR 20®**) has been licensed in the UK from 24^th^ September 2024 as a 2+1 and 3+1 schedule from 6 weeks to 15 months of age, but it is not yet part of the routine immunisation schedule. Given the potential benefits of a reduced dosing schedule, additional studies are warranted to comprehensively evaluate the suitability a 1+1 dosing schedule or the current 2+1 schedule.

Infants currently receive two doses of the PCV13 vaccine at ages 3 and 12 months, following the UK immunization schedule. Given the ongoing problem of serotype replacement this study will compare the immune response of PCV15 and PCV20 given at 3 or 4 months and 12 months as 1+1 schedule and PCV20 as 2+1 schedule and determine whether these are non-inferior to the current PCV13 vaccine.

Recent, unpublished, findings from the “LION MenB” study (REC Reference 21/NE/0093) conducted by Prof. Paul Heath indicated that vaccinating infants with 4CMenB vaccine at 2 and 3 months, rather than the current 2- and 4-months schedule, would allow earlier protection against invasive meningococcal disease (IMD) without compromising long-term immunity^19^. In The LION MenB Study the first dose of PCV13 was given at 4 months, instead of 3 months, to accommodate 4CMenB being administered at 3 months^19^.

The LION MenB data became available after the commencement of enrolment for this current protocol and it was only at this point that a change to the UK-infant immunisation schedule was raised. LION MenB’s data shifted the 4CMenB dose earlier to 3 months and the first PCV dose shifted later to 4 months.

With changes to the UK schedule, this protocol has been amended to include an exploratory objective comparing immunogenicity of 1+1 PCV schedules when the first dose of PCV13 or PCV15 or PCV20 is given at 3 months vs 4 months of age. For this, participants randomised to Arms 1, 2 and 4 will be further randomised 1:1 to receive their first dose of PCV13 or PCV15 or PCV20 at 3 months (same as original arm 1, 2 or 4) or at 4 months (arm 1r4, 2r4 or 4r4)* and then measuring serotype specific-specific pneumococcal antibodies at least 4 weeks post-prime and at least 4 weeks post-booster dose. Participants enrolled in the trial prior to SA03 being enacted will experience no change.

*Note on the naming of study arms: 1r4, 2r4, 4r4 – participants randomised to receive the PCV vaccine from that arm at 4-months rather than 3-months

In May 2025, the JCVI recommended the cessation of routine Hib/MenC (Menitorix) to infants turning 1 year old as the manufacturer has ceased production of Menitorix vaccine. As a result, this protocol is amended so that the Hib/MenC (Menitorix) vaccine is not offered to infants participating in this study.

In August 2025, the UK government announced the introduction of a chickenpox (varicella) vaccination programme in England, commencing in January 2026. To align with the revised national immunisation schedule, this protocol has been amended such that participants will be offered either the MMR or MMRV vaccine in accordance with the current national immunisation schedule at the time.

The study is funded by NISEC and sponsored by University of Oxford. The study is being conducted by Oxford Vaccine Group.

# 5. OBJECTIVE AND OUTCOMES MEASURES

| **Objectives** | **Outcome Measures** | **Timepoint(s) of evaluation of this outcome measure (if applicable)** | | |
| --- | --- | --- | --- | --- |
| **Primary Objective:**  To determine if the PCV13 serotype-specific serum IgG concentrations is non-inferior in homologous arms 1/1r4 & 2/2r4 at 13 months in comparison to the control arm 4/4r4. | PCV13 serotype-specific serum IgG concentration. | At 13 months of age (at least 4 weeks after administration of the last dose of PCV vaccine). | | |
| **Secondary Objectives:**  To determine if the PCV13 serotype-specific serum IgG concentrations is non-inferior in arm 3 in comparison to the control arm 4/4r4. | PCV13 serotype-specific serum IgG concentration. | At 13 months of age (at least 4 weeks after administration of the last dose of PCV vaccine). | | |
| To characterise the seven additional PCV20 serotype-specific serum IgG concentrations following homologous PCV schedules in arm 1/1r4, 2/2r4 and 3 in comparison to the control arm 4/4r4 | PCV20 serotype-specific serum IgG concentrations (Serotypes 8, 10A, 11A, 12F, 15B, 22F and 33F) | | At 13 months of age (at least 4 weeks after administration of the last dose of PCV vaccine) |  |
| To determine the safety and reactogenicity of different schedules with PCV15 (1+1) and PCV20 (1+1) and (2+1) | Solicited AEs  Unsolicited AEs  SAEs | | within 7 days  within 28 days  During the whole study period |  |
| **Exploratory Objectives:**  To determine if vaccine induce IgG responses at the mucosa (nasal) and if antibody levels in the nose correlate with levels in blood. | PCV13 serotype specific nasal IgG GMC against some of the PCV serotypes (sample volume depending) | | At 13 months of age (at least 4 weeks after administration of the last dose of PCV vaccine). |  |
| To determine the PCV20 serotype-specific IgG concentration following one dose of PCV13, PCV15 or PCV20. | PCV20 serotype-specific serum IgG concentrations | | At 4/5 months. |  |
| To determine the PCV20 serotype-specific IgG concentration following two doses of PCV20 (2+1) schedule. | PCV20 serotype-specific serum IgG concentrations | | At 5 months. |  |
| To detect pneumococcal colonisation and serotypes present. | Molecular microbiology (fluidic qPCR for detection of lytA, paiB and serotyping) | | At 13 months of age (at least 4 weeks after administration of the last dose of PCV vaccine). |  |
| To determine immunogenicity against concomitant vaccines given as part of the infant routine immunisation schedule | IgG Antibody GMCs against pertussis antigen (pertussis toxin, filamentous hemagglutinin and pertactin), diphtheria toxoid, tetanus toxin and Hib capsular polysaccharide;  SBA GMT against MenB | | At 13 months of age (at least 4 weeks after administration of the last dose of PCV vaccine).  SBA GMTs against MenB at 4/5 months (at least 4 weeks after second dose of MenB vaccine) and 13 months. |  |
| To compare immunological responses to PCV post priming and post boosting dose for infants given PCV vaccines at 4 and 12 months of age to infants given PCV vaccines at 3 and 12 months of age. | PCV serotype-specific serum IgG concentrations | | 4 or 5 months of age (at least 4 weeks post first dose of PCV vaccine) and at 13 months of age (at least 4 weeks post second dose of PCV vaccine) |  |

# 6. TRIAL DESIGN

The study will be open label, randomised, controlled and multicentre with sample size of 600 participants recruited and assigned into four arms with 150 participants in each arm. Participants randomised to Arms 1, 2 and 4 will be further randomized 1:1 to receive their first dose of PCV13 or PCV15 or PCV20 at three months of age (same as the original arm 1, 2 or 4) or at four months of age (arm 1r4, 2r4 or 4r4).

Figure 1: Randomisation schematic

Healthy children will be enrolled at approximately 2 months (+2 weeks) of age. Participants in Arms 1, 2 and 4 will receive 2 doses of either PCV15, 20 or 13 at 3 and 12 months of age and participants in Arms 1r4, 2r4 and 4r4 will receive 2 doses of either PCV15, 20 or 13 at 4 and 12 months of age. While arm 3 will follow 2+1 schedule and will receive 2 doses of PCV20 at 2 months and 4 months and a booster dose at 12 months.

All participants will receive the second dose of the MenB vaccine at 3 months of age, in accordance with the revised infant immunization schedule effective from 1st July 2025.

Participants in arms 1, 2, 3 and 4 already enrolled in the protocol prior to the implementation of SA04 will also receive the second dose of the MenB vaccine at 3 months of age, provided they have not already received their 3-months vaccination.

Participant who had their 3-months vaccination before implementation of SA04 will receive second dose of MenB at 4-months vaccination or earlier at an additional visit if required.

Table 1. Study schedule for vaccines and sampling for Arm 1, 2, 3 and 4

| **Visit number** | **V1** | **V2** | **V3** | **V3 (A)** | **V4** | **V5** | **Additional †** |
| --- | --- | --- | --- | --- | --- | --- | --- |
| **Participant age** | 2 months* | 3 months* | 4 months* | 5 months* | 12 months | 13 months +* | 3 – 4 months* |
| **Blood sample for Arms 1, 2 and 4** | - | - | Yes |  | - | Yes | - |
| **Blood sample for Arm 3** |  |  |  | Yes |  | Yes | - |
| **Nasal sample (mucosal lining fluid) for Arms 1, 2 and 4** |  | Yes | Yes |  | - | Yes | - |
| **Nasal sample (mucosal lining fluid) for Arm3** | Yes | Yes |  | Yes |  | Yes | - |
| **Arms 1, 2,3, 4** | 6:1  MenB  Rotavirus | 6:1  MenB  Rotavirus | 6:1  MenB*** |  | MenB  MMR or MMRV** | - | MenB*** |
| **Arm 1** | - | PCV15 | - |  | PCV15 | - |  |
| **Arm 2** | - | PCV20 | - |  | PCV20 | - |  |
| **Arm 3** | PCV20 |  | PCV20 |  | PCV20 | - |  |
| **Arm 4** | - | PCV13 | - |  | PCV13 | - |  |

* Month will be defined as 28 days.

** depending on the standard national immunisation schedule at the time of the visit

*** 2nd dose of MenB to be given at visit 3 or Additional visit for participant who already had their visit 2 prior to the implementation of SA04

† Optional, as required

Table 1.1. Study schedule for vaccines and sampling for Arm 1r4, 2r4 and 4r4.

| **Visit number** | **V1** | **V2** | **V3** | **V3 (A)** | **V4** | **V5** |
| --- | --- | --- | --- | --- | --- | --- |
| **Participant age** | 2 months* | 3 months* | 4 months* | 5 months* | 12 months | 13 months +* |
| **Blood sample** |  |  |  | Yes |  | Yes |
| **Nasal sample (mucosal lining fluid)** |  |  | Yes | Yes |  | Yes |
| **Arms 1r4, 2r4 and 4r4** | 6:1  MenB  Rotavirus | 6:1  MenB  Rotavirus | 6:1 |  | MenB  MMR or MMRV** |  |
| **Arm 1r4** |  |  | PCV15 |  | PCV15 |  |
| **Arm 2r4** |  |  | PCV20 |  | PCV20 |  |
| **Arm 4r4** |  |  | PCV13 |  | PCV13 |  |

Along with the PCV vaccine, nasal samples and blood samples will be taken as per Table 1 and Table 1.1.

* Month will be defined as 28 days.

** depending on the standard national immunisation schedule at the time of the visit

All enrolled participants will also receive other scheduled childhood immunizations that are needed over the course of treatment as specified in Table 1 and Table 1.1.

**6.1	Vaccine abbreviations:**

**6:1 is a combination vaccine containing:** (DTaP-IPV-Hib-HepB) diphtheria (D), tetanus (T), pertussis (acellular component) (Pa), hepatitis B (rDNA) (HBV), poliomyelitis (inactivated (IPV) and Haemophilus influenza type b (Hib) conjugated vaccine (adsorbed); Infanrix hexa® or Vaxelis®
**MenB**: meningococcal serogroup B vaccine adsorbed rDNA vaccine Bexsero®**Rotavirus:** live attenuated vaccine rotavirus vaccine- Rotarix®**MMR**: live attenuated vaccine against measles, mumps and rubella - Priorix ® or MMRvaxPro®
MMRV: live attenuated vaccine against measles, mumps, rubella and varicella - ProQuad® or Priorix Tetra®

# 7. PARTICIPANTS IDENTIFICATION

## 7.1. Trial Participants

Participants must fulfil all four inclusion criteria and none of the exclusion criteria to be eligible to participate.

## 7.2. Inclusion Criteria

- Infants due to receive their primary immunisations, aged up to 2 months (+ 2weeks) at first vaccinations.
- Infants born at ≥ 37 weeks of gestational age.
- Parent(s) or legal guardian(s) willing and able to follow the requirements of the protocol for the duration of the study.
- Written informed consent given by parent(s) or legal guardian(s) who is aged ≥16 years.

## 7.3. Exclusion Criteria

- Prior receipt of vaccines. (Except of Hepatitis B or BCG vaccine)
- Prior planned receipt of Investigation vaccines.
- Current participation in other research study, except if the study is solely observational.
- Children of parents who are on the delegation log for this study
- A confirmed anaphylactic reaction to neomycin, streptomycin or polymyxin B (which may be present in trace amounts in the tetanus vaccine) and/or kanamycin, histidine, sodium chloride or sucrose (which may be present in trace amounts in the MenB vaccine).
- Latex hypersensitivity (the syringe cap of Bexsero may contain natural rubber latex)
- Major congenital defects or serious chronic illness
- Presence of an evolving or changing neurological disorder
- Presence of central nervous system disease or convulsions in the infant.
- Bleeding disorder
- Confirmed or suspected immunodeficiency
- A family history of congenital or hereditary immunodeficiency
- Receipt of more than 1 week of immune-suppressants or immune modifying drugs (e.g., oral prednisolone >0.5ml/kg/day or intravenous glucocorticoid steroid). Nasal, topical or inhaled steroids are allowed
- Administration of immunoglobulin and/or any blood products since birth or planned administration during the study period
- History of allergy to any component of the vaccines.
- Any other significant disease or disorder which, in the opinion of the Investigator, may either put the participants at risk through participation in the study, or may influence the result of the study or the participant’s ability to participate in the study.

## 7.4. Temporary Exclusion Criteria

Children are temporarily excluded from participating if they:

- Have received BCG and Hepatitis B vaccine within 14 days prior to study vaccines
- Have scheduled elective surgery, planned admission or other procedures requiring general anaesthesia within 7 days of receiving a vaccine
- In the presence of an acute illness or the presence of fever ≥38ºC, until 24 hours after resolution
- Blood sampling will also be postponed for seven days after completion of any antibiotic course to ensure no interference with laboratory tests.

# Have received any live-attenuated vaccines (e.g., chickenpox) within 4 weeks prior to administration of the study vaccine at Visit 4. 8. TRIAL PROCEDURE

## 8.1 Recruitment

Potential participants will be identified and approached using the following recruitment strategies:

- MAIL-OUT: age-appropriate children may be identified via the Child Health Information Service (CHIS) or other National Health Service equivalent databases. Initial contact to potential participants will not be made by the study team. Instead, study invitation material will be sent out on behalf of the study team by an external company, CFH Docmail Ltd or equivalent, to preserve the confidentiality of potential participants. CFH Docmail Ltd is accredited as having exceeded standards under the NHS Digital Data Security and Protection Toolkit (ODS ID – 8HN70). Potential participants will be invited to either register their interest directly online using a QR code to complete REC-approved screening questionnaires on the trial websites, to return a reply slip in the sealed freepost envelope (if included with the invitation letter), or to contact the study team per email or telephone. Replies will be processed and stored in accordance with local procedures surrounding the handling of identifiable data.
- Direct mail-out by the local site to potential participants identified using the local site’s birth registry information. This data would not be transferred between sites.
- VOLUNTEER DATABASES: the study may be advertised on the electronic newsletter sent out to parents signed up to the Oxford Vaccine Group’s Children and Young Peoples Database. Members of the public who have registered on this secure database have given their consent to be contacted when studies open for recruitment and understand that there is not a commitment to participate.
- ROYAL MAIL LEAFLET: Royal Mail door-to-door service with delivery of invitation letters in site envelopes to every household within certain postcode areas.
- WEBSITE ADVERTISING: Description of the study and a copy of the study information booklet on site websites or other appropriate websites
- SOCIAL MEDIA: Advertisements placed on trial site media accounts or targeted social media platform advertisements including, but not restricted to, Twitter, Facebook and Instagram
- Advertisement of the study in public places, including buses and trains with the agreement of the owner/ proprietor, in newspapers or other literature for circulation and on radio and/or television via announcements.
- Using local GP practices or hospital trusts as Participant Identification Centres (PICs)
- Using direct SMS/text message, or emails to potential participants identified by GPs from their databases.

Those parents/legal guardians that indicate that they do not want to take part in the study and/or receive further communication about the study will not be included in any subsequent contact lists.

## 8.2 Screening and Eligibility Assessment

Parents who are interested in taking part will be able to contact the study team by telephone, email, by completing the online screening questionnaire or by returning the attached reply-slips. The study team will review responses to the online questionnaire and will then contact the family via either phone or email to book the first visit. Where an online screening questionnaire has not been completed, a broad discussion on eligibility criteria will take place by phone or email before arranging the first visit.

Potentially eligible children whose parents are interested in the study will be visited at their home or in a clinic depending on site preference, where informed consent will be sought, and infants enrolled/immunised as appropriate. The participant’s GP and the Child Health Information Service will be notified of all immunisations administered in the study.

## 8.3 Informed Consent

The parent/legal guardian of the participant will personally sign and date the latest approved version of the Informed Consent Form before any study procedures are performed.

The parent/legal guardian will have the opportunity to discuss the study with a medically qualified investigator/researcher. Written informed consent will be obtained by means of a dated signature of the parent/legal guardian and a signature of the appropriately trained and delegated clinician/researcher. A copy of the signed informed consent will be given to the participant, and the original signed form will be retained at the study site.

A written version and verbal explanation of the study information booklet will be provided to the parent/legal guardian, detailing the exact nature of the trial, including the study schedule, what is involved, the implications and constraints of the protocol and any study procedures at least 24 hours prior to the first visit.

It will also provide information about known side effects, including those of the study vaccinations, and risks involved in taking part. It will be clearly stated that the parent/legal guardian is free to withdraw their child from the trial at any time for any reason without prejudice to future care, without affecting their legal rights and with no obligation to give the reason for withdrawal. The parent/legal guardian will be informed that anonymised samples taken during the study may be shared with study collaborators. Any left-over samples will be transferred to Oxford Vaccine Centre (OVC) Biobank (REC 21/SC/0161) for storage once the sample is no longer required for the study endpoints, if consent to do so has been obtained. The OVC Biobank study is covered by a separate study protocol and consent process. If parents/guardians do not consent to biobank storage, then all samples will be destroyed at the end of the study.

The study team will request permission to contact the child’s GP and/or other treating doctors for medical and vaccine history, if required. All sites will make possible attempts to contact child’s health visitor in accordance with their local SOPs, before the visit. The study team will also request permission to inform the child’s GP that the child is taking part in the study and that we will be administering their routine vaccinations between 2 and 12 months of age. The participant’s parents/legal guardians will be allowed as much time as they wish to consider the information, and given the opportunity to question the Investigator, their GP, or other independent parties to decide whether to participate in the study, as long as the participant is still in the enrolment age window and the study is still open for recruitment. Written Informed Consent will then be obtained by means of a dated signature, together with the dated signature of the person who presented and obtained the Informed Consent. The person obtaining the consent will be suitably qualified and experienced, have been authorised to do so by the Chief/Principal Investigator and be listed on the delegation log. A copy of the signed Informed Consent will be given to the participant’s parents/ legal guardians. A copy of the separate Biobank consent form will also be given to the participant’s parents/legal guardians (if applicable). The original signed forms will be retained at the study sites.

## 8.4 Randomisation procedure

Participants will be randomized to a 1:1:1:1: ratio before their first vaccination visit into four arms to receive PCV vaccine as per the trial design mentioned in table 1. For participants randomised to arm 1, 2 and 4, we will further randomise their timing of first dose of PCV vaccine at 3 or 4 months with a ratio of 1: 1.
Computer generated randomisation lists will be prepared by the study statistician at the Oxford Vaccine Group using stratified block randomisation. Random block sizes of 4 or 8 will be used for the primary randomisation. For the randomisation of prime dose timing, random block sizes of 2 and 4 will be used. The randomisation lists will be stratified by study site. The randomisation list will be loaded to a central randomisation system and each site user will have a unique log-in to access their corresponding randomisation list. A detailed description of the randomisation process will be described in the study training materials.

# 9 STUDY VISITS

Study will consist of total of up to six visits which will be conducted by study staff at the participant home, at convenient and suitable site venues, or in the community with appropriate permission in place (e.g., GP practices or local hospitals).

Before visit 1, each participant will be randomised into one of the four main study arms and arms 1, 2 and 4 will be further randomized 1:1 to receive their first dose at three months (same as the original arm 1, 2, 4) or at four months (arm 1r4, 2r4 or 4r4) (see Table 1) using procedures outlined in Section 8.4.

## 9.1 Visit 1 – Enrolment and vaccination at 8 – 10 Weeks of age

- Answer any questions the family has about the study having read the PIS.
- Obtain written informed consent from the parent/guardian
- Give a brief explanation of biobank and leave a study booklet for biobank for parent/guardian.
- Perform thorough check of inclusion and exclusion criteria and record findings, including medical and vaccination history, of relevance to the inclusion/ exclusion criteria
- Note any details and indications for any prescription medications for infant in the CRF.
- Record/confirm demographic data including date of birth and gender, for subsequent data analysis.
- Physical examination
  - Must be performed if the 6-8 week health development review has not been performed
  - At investigator discretion if indicated by medical history
- Measure and record axillary temperature

If all inclusion criteria are satisfied, and there are no exclusion criteria, the participant may be enrolled into the study.

- Collect Mucosal Lining Fluid (MLF) using synthetic absorptive matrices (SAM) for arm 3. Any leftover sample at the end of the study will be adopted into Biobank if consented.

Then administer the following vaccines:

(i) DTaP-IPV-Hib-HepB via intra-muscular injection into the right upper anterolateral thigh
(ii) 4CMenB (Bexsero) via intramuscular injection into the left anterolateral thigh.
(iii) PCV20 (**Apexxnar/Prevenar 20**) via intramuscular injection into the right lower anterolateral thigh (for Arm 3 only).
(iv) Rotavirus (Rotarix) drops via oral route.

- Observe for 15 minutes after vaccination for any significant acute reactions, with adrenaline readily available in case of anaphylactic reaction. Any adverse events (AEs) that occurred during the observation period should be recorded.
- Issue e-Diary for arm 3. A paper back-up diary will be provided in case the e-Diary cannot be accessed. Give login details to parent/legal guardian for the e-Diary using their personal email address for the set-up. Demonstrate e-Diary/ paper back-up diary to parents/legal guardians for recording AEs and check their understanding
- Issue a digital thermometer and ruler. Explain how to measure and record temperature, local and systemic reactions, and how to record adverse events and concomitant medications. Temperature and local and systemic reactions to vaccination should be recorded for 7 (D0-D6) days post vaccination
- Instruct the parents to give paracetamol as soon as possible after vaccination to reduce the risk of fever after MenB vaccine.
- Ask parent/ guardian to notify study team of any serious adverse events/reactions.
- Complete the “red book” (parent held child record) if available.
- Arrange visit 2

## 9.2 **Visit** 2 – Vaccination and Nasal sampling visit at 28-42 days post V1:

- Check inclusion/exclusion criteria are still valid and check temporary exclusion criteria for vaccination and blood sampling visit.
- Obtain written informed consent from the parent/guardian for storage of any leftover clinical samples into Biobank.
- Physical examination (if required)
- Review e-Diary/ for arm 3. Collect backup paper diary if issued.
- Record and report any AEs that have occurred since the last visit for arm 3
- Record and report any SAE that have occurred since last visit for all arms
- Review medical history and record any new concomitant medications administered for AEs/SAEs.
- Measure and record axillary temperature
- Collect Mucosal Lining Fluid (MLF) using synthetic absorptive matrices (SAM) for arm 1, 2, 3 and 4. Any leftover sample at the end of the study will be adopted into Biobank if consented.

Then administer the following vaccine:

(i) DTaP-IPV-Hib-HepB via intra-muscular route into the right upper antero-lateral thigh.
(ii) 4CMenB (Bexsero) via intramuscular injection into the left anterolateral thigh
(iii) Rotavirus (Rotarix) drops via oral route.
(iv) PCV vaccination via intramuscular route into the right lower anterolateral thigh, as allocated to each participant in each group.

**Arm 1** PCV15 (Vaxneuvance)

**Arm 2** PCV20 (Apexxnar/Prevenar 20)

**Arm 4** PCV13 (Prevenar13)

- Observe for 15 minutes after vaccination for any significant acute reactions, with adrenaline readily available in case of anaphylactic reaction. Any adverse events (AEs) that occurred during the observation period should be recorded.
- Issue e-Diary for arms 1, 2 and 4. A paper back-up diary will be provided in case the e-Diary cannot be accessed. Give login details to parent/legal guardian for the e-Diary using their personal email address for the set-up. Demonstrate e-Diary/ paper back-up diary to parents/legal guardians for recording AEs and check their understanding.
- Issue a digital thermometer and ruler. Explain how to measure and record temperature, local and systemic reactions, and how to record adverse events and concomitant medications. Temperature and local and systemic reactions to vaccination should be recorded for 7 (D0-D6) days post vaccination
- Instruct the parents to give paracetamol as soon as possible after vaccination to reduce the risk of fever after MenB vaccine.
- Ask parent/ guardian to notify study team of any serious adverse events/reactions
- Complete the “red book” (parent held child record) if available.
- Arrange visit 3.

## 9.3 Visit 3 – Vaccination and sample collection at 28-42 days post V2.

- Check inclusion/exclusion criteria are still valid and check temporary exclusion criteria for vaccination visit.
- Obtain written informed consent from the parent/guardian for storage of any leftover clinical samples into Biobank. (if not consented at previous visit).
- Review e-Diary/diary card for arms 1, 2 and 4. Collect any issued backup paper diary.
- Record and report any AEs/SAEs that have occurred since the last visit for arms 1, 2 and 4.
- Record and report any SAE that have occurred since last visit for all arms.
- Review medical history and record any new concomitant medications administered for AEs/SAEs.
- Physical examination (if required)
- Measure and record axillary temperature
- Collect Mucosal Lining Fluid (MLF) using synthetic absorptive matrices (SAM) for arms 1, 2 and 4, and arms 1r4, 2r4 and 4r4. Any leftover sample at the end of the study will be adopted into Biobank if consented.
- Ensure local anaesthetic cream has been applied (allowing sufficient time between application and venepuncture, in accordance with local SOPs).
- Perform blood sampling and collect a maximum of 4ml of venous blood, in accordance with local SOPs for arms 1, 2 and 4. Any leftover sample at the end of the study will be adopted into Biobank if consented.

Then administer the following vaccines:

(i) DTaP-IPV-Hib-HepB via intra-muscular route into the right upper anterolateral thigh (or into the left thigh, if 4CMenB is not being administered)
(ii) 4CMenB (Bexsero) via intramuscular injection into the left anterolateral thigh (**for Arms 1, 2, 3 and 4**). (2nd dose of MenB to be given at visit 3 for participant who already had their visit 2 before the implementation of SA04)
(iii) PCV vaccination via intramuscular route into the right lower anterolateral thigh, as allocated to each participant in each group.

**Arm 1r4** PCV15 (Vaxneuvance)

**Arm 2r4** PCV20 (Apexxnar/Prevenar 20)

Arm 3 PCV20 (Apexxnar/Prevenar 20)

**Arm 4r4** PCV13 (Prevenar 13)

- Observe for 15 minutes after vaccination for any significant acute reactions, with adrenaline readily available in case of anaphylactic reaction. Any adverse events (AEs) that occurred during the observation period should be recorded.
- Issue e-Diary for arms 1r4, 2r4, 3 and 4r4. A paper back-up diary will be provided in case the e-Diary cannot be accessed. Give login details to parent/legal guardian for the e-Diary using their personal email address for the set-up. Demonstrate e-Diary/ paper back-up diary to parents/legal guardians for recording AEs and check their understanding
- Issue a digital thermometer and ruler. Explain how to measure and record temperature, local and systemic reactions, and how to record adverse events and concomitant medications. Temperature and local and systemic reactions to vaccination should be recorded for 7 (D0-D6) days post vaccination.
- Instruct the parents to give paracetamol as soon as possible after vaccination to reduce the risk of fever after MenB vaccine. (* for those having 2nd dose of MenB at visit 3)
- Ask parent/ guardian to notify study team of any serious adverse events/reactions.
- Complete the “red book” (parent held child record) if available.
- Arrange visit 3(A) for arms 1r4, 2r4, 3 and 4r4 and visit 4 for arms 1, 2 and 4.

## 9.4 Visit 3(A) – Sample collection at 28-42 days following V3 for arms 1r4, 2r4, 3 and 4r4 only.

- Check inclusion/exclusion criteria are still valid and check temporary exclusion criteria applicable to blood sampling visit.
- Obtain written informed consent from the parent/guardian for storage of any leftover clinical samples into Biobank. (if not consented at previous visits).
- Review e-Diary/diary card.
- Record and report any AEs/SAEs that have occurred since the last visit.
- Review medical history and record any new concomitant medications administered for AEs/SAEs
- Physical examination (if required)
- Collect Mucosal Lining Fluid (MLF) using synthetic absorptive matrices (SAM). Any leftover sample at the end of the study will be adopted into Biobank if consented.
- Ensure local anaesthetic cream has been applied (allowing sufficient time between application and venepuncture, in accordance with SOP).
- Perform blood sampling and collect a maximum of 5 ml of venous blood, in accordance with local SOPs. Any leftover sample at the end of the study will be adopted into Biobank if consented.

## 9.5 Visit 4 – Vaccination visits at 12 months (+ 14 days) of age

- Check inclusion/exclusion criteria are still valid and check temporary exclusion criteria for vaccination visit.
- Obtain written informed consent from the parent/guardian for storage of any leftover clinical samples into Biobank. (if not consented at previous visits).
- Record and report any SAEs that have occurred since the last visit for all arms.
- Review medical history and record any new concomitant medications administered for SAEs.
- Measure and record axillary temperature
- Physical examination (if required)

Then administer following vaccines:

(i) 4CMenB (Bexsero) intramuscular injection into the left anterolateral thigh.
(ii) Depending on the current approved national immunisation schedule, either MMR (MMRvaxPro/Priorix) intramuscular injection into right deltoid or right upper anterolateral thigh. (If deltoid muscle bulk is deemed inadequate) or MMRV (ProQuad/Priorix Tetra) intramuscular injection into right deltoid or right upper anterolateral thigh. (If deltoid muscle bulk is deemed inadequate).
(iii) 2^nd^ dose (for arms 1, 2 and 4 and arms 1r4, 2r4 and 4r4) or 3^rd^ dose (for arm 3) of PCV vaccination via intramuscular route into the right lower anterolateral thigh, as allocated to each participant in each group.

Arm 1/1r4 PCV15 (Vaxneuvance)

Arm 2/2r4 PCV20 (Apexxnar/Prevenar 20)

Arm 3 PCV20 (Apexxnar/Prevenar 20)

Arm 4/4r4 PCV13 (Prevenar13)

- Observe for 15 minutes after vaccination for any significant acute reactions, with adrenaline readily available in case of anaphylactic reaction. Any adverse events (AEs) that occurred during the observation period should be recorded.
- Issue e-Diary. A paper back-up diary will be provided in case the e-Diary cannot be accessed. Give login details to parent/legal guardian for the e-Diary using their personal email address for the set-up. Demonstrate e-Diary/ paper back-up diary to parents/legal guardians for recording AEs and check their understanding.
- Issue a digital thermometer and ruler. Explain how to measure and record temperature, local and systemic reactions, and how to record adverse events and concomitant medications. Temperature and local and systemic reactions to vaccination should be recorded for 7 (D0 – D6) days post vaccination.
- Instruct the parents to give paracetamol as soon as possible after vaccination to reduce the risk of fever after MenB vaccine.
- Ask parent/ guardian to notify study team of any serious adverse events/reactions.
- Complete the “red book” (parent held child record) if available.
- Arrange visit 5

## 9.6 Visit 5 - Blood and nasal sample collection visit 28-42 days following V4.

- Check inclusion/exclusion criteria are still valid and check temporary exclusion criteria applicable to blood sampling visit.
- Obtain written informed consent from the parent/guardian for storage of any leftover clinical samples into Biobank. (if not consented at previous visits).
- Review e-Diary/diary card.
- Record and report any AEs/SAEs that have occurred since the last visit for all arms.
- Review medical history and record any new concomitant medications administered for AEs/SAEs.
- Physical examination (if required)
- Collect Mucosal Lining Fluid (MLF) using synthetic absorptive matrices (SAM) for all arms. Any leftover sample at the end of the study will be adopted into Biobank if consented.
- Ensure local anaesthetic cream has been applied (allowing sufficient time between application and venepuncture, in accordance with SOP).
- Perform blood sampling and collect a maximum of 6 ml of venous blood for all arms, in accordance with local SOPs. Any leftover sample at the end of the study will be adopted into Biobank if consented.

## 9.7 Additional Visit – MenB vaccination only, between Visit 2 and Visit 3

For use as an optional visit for those participants who completed their 3-month vaccination and did not yet receive 4-month vaccinations, before SA04 was implemented

- Obtain written informed consent from the parent/guardian
- Confirm participant is well and fit for vaccination
- Administer 4CMenB (Bexsero) via intramuscular injection into the left anterolateral thigh
- Observe for 15 minutes after vaccination for any significant acute reactions, with adrenaline readily available in case of anaphylactic reaction. Any adverse events (AEs) that occurred during the observation period should be recorded.
- Instruct the parents to give paracetamol as soon as possible after vaccination to reduce the risk of fever after MenB vaccine.
- Ask parent/ guardian to notify study team of any serious adverse events/reactions.
- Complete the “red book” (parent held child record) if available.

## 9.8 Blood sampling

Blood sampling will be carried out in accordance with local SOPs at each study visit. A topical anaesthetic cream will be offered prior to each venepuncture. A maximum of 4ml of blood will be obtained at visit 3, maximum of 5ml at visit 3(A)and maximum of 6ml of blood will be obtained at visit 5 for laboratory analysis. If the initial attempt at venepuncture draws less than the required amount, verbal consent will be sought for one further attempt at that visit. If two attempts at venous sampling have resulted in no blood being drawn, a heel/finger prick may be attempted (but only with parental approval and if site SOPs permit). If no blood sample is obtained, an additional visit (to attempt venepuncture again) may be rescheduled for another day within the visit timelines, with parental/legal guardian consent.

Blood samples collected at the visits will be transported to the laboratory in a cool bag at 2 - 8 degrees Celsius and will then be centrifuged, separated and frozen at -80 degrees within 24 hours of collection. Samples from other study sites will be shipped to Oxford study site (OVG) before sending them for testing in GOSH laboratory.

Samples for assessment of serotype-specific IgG concentration for PCV13, PCV15 and PCV 20 will be shipped to the following laboratory for testing:

- GOSH Laboratory (Guildford Street, London, WC1N 1EH): PCV13, PCV15 and PCV20 serotype-specific serum IgG

A small volume of some sera left over after testing may be used to assess pneumococcal IgG concentrations on more than one pneumococcal IgG assay in this laboratory as part of assay validation for infant studies.

All other non-PCV antigens will be shipped to the following below mentioned laboratories:

- UKHSA Vaccine Evaluation Unit, Clinical Sciences Building 2, Manchester Royal Infirmary, Oxford Road Manchester M13 9WL:
- SBA GMT against MenB
- RIVM, Centre for Infectious Disease Control (Antonie van Leeuwenhoeklaan 9, 3721 MA Bilthoven, the Netherlands):
- Hib capsular polysaccharide, diphtheria toxoid, tetanus toxin and pertussis antigens (pertussis toxin, filamentous hemagglutinin, and pertactin)

## 9.9 Mucosal Lining fluid (MLF) sampling

Mucosal samples will be collected at different visits depending on the study arm (see table 1 and 1.1) They will be transported to the laboratory in a cool bag at the temperature of 2-8 degree Celsius and will be stored at in a freezer at -80 degrees prior to transfer to OVG laboratory. Nasal absorption is performed by manoeuvring a strip of synthetic absorptive matrices (SAM) up the lumen of the nostril, avoiding rubbing against the nasal mucosa. The outside of the nose is then pressed with a finger to cause apposition of the SAM against the mucosa and holding the strip for 60 seconds for better absorption. The procedure may tickle slightly but is painless, and MLF can be obtained even from non-inflamed noses at frequent intervals, without the need for local anaesthetic. There is minimal protein binding to the SAM strip, and fluid can be eluted by spin filtration. High levels of mediators of inflammation can then be measured in the MLF: higher than detectable by nasal lavage.

## 9.10 Recording symptoms: e-Diary

The parents/legal guardians of the participant will be asked to maintain an electronic diary (or a back-up paper diary), detailing all solicited reactions from the day of vaccination and for 6 days following vaccination each time the participant receives a PCV vaccine (as outlined in 21. Schedule of Events). If a symptom persists after 6 days, an end-date should be entered, if available; otherwise, the symptom should be recorded as “ongoing”. A daily temperature measurement will be recorded as part of the diary. There will be space to record any other symptoms that occur in the 6 days post vaccination. The e-Diary will be on a secure website hosted by the University of Oxford and will be using the parent personal email address for the set-up and to receive the notification to complete

When a grade 3 symptom is entered by the parents of enrolled participants, the clinical staff will be alerted through an email. Telephone/email contact may be made with participant’s parent, if there are data that need to be clarified. Parents/legal guardians will be provided with a 24-hour phone number to access a member of the study team, should urgent advice be required following vaccination.

If a parent or guardian cannot enter data electronically, they will use a backup paper diary to record solicited reactions. Staff will collect it at the next study visit and will transfer data from this into the relevant system. The paper records will remain with the site and be stored in the ISF at the end of the study.

## 9.11 Clinical samples for Biobank storage

Parents/legal guardians will be asked for consent to store the remaining blood samples and nasal samples, taken at any time during the study, at the Oxford Vaccine Centre biobank (REC 21/SC/0161):

• Storage of serum remaining after study tests have been performed.

• Storage of clot remaining from serum tube, which would otherwise be discarded during processing.

- Storage of mucosal lining fluid after study tests have been performed.

There will be a separate information sheet and consent form for this. Parents/guardians will be free to decline this, without prejudicing their child’s participation in the main study. Consent for biobank would preferably be obtained during the first study visit but could be also obtained during the other study visits.

## 9.12 Definition of enrolment

The point of enrolment is defined as when the infant is consented and eligible for the study.

## 9.13 Early Discontinuation or withdrawal of participants

The parents/legal guardians of participants have the right to withdraw them from the study at any time, for any reason. In addition, the Investigator may discontinue a participant from the study at any time if the Investigator considers it necessary for any reason, including:

- Ineligibility: either arising during the trial, or retrospectively (having been overlooked at screening)
- Significant protocol deviation
- Significant non-compliance with treatment regimen or study requirements
- An adverse event which requires discontinuation of the study vaccines or results in inability to continue to comply with study procedures
- Disease progression which requires discontinuation of vaccination or results in inability to continue to comply with study procedures
- Withdrawal of Consent
- Loss to follow-up

Data obtained from participants before withdrawal will continue to be analysed for the study. Parents/ legal guardians may request that their child’s samples are destroyed, unless they have already been analysed.

The reason for withdrawal will be recorded in the CRF (if available).

If the participant is withdrawn due to an adverse event, the Investigator will arrange for follow-up visits or telephone calls until the adverse event has resolved or stabilised. At the study end, the final status of the adverse event should be recorded.

## 9.14 Definition of end of trial

The end of trial will be when laboratory analysis of the primary and secondary endpoints has been completed for all biological samples. All visits will have been completed by this point.

# 10 TRIAL INTERVENTIONS

## 10.1 Investigational Medical Products (IMPs) Description:

## 10.1.1 PCV13 (PREVENAR13®):

PREVENAR13 (Pfizer) a pneumococcal conjugate vaccine that contains 13 different pneumococcal serotypes (1, 3, 4, 5, 6A, 6B, 7F, 9V, 14, 18C, 19A, 19F and 23F) and is licensed in Europe. It is part of the UK routine schedule at 4 and 12 months of age.

One dose (0.5 ml) will be injected at each vaccination.

## 10.1.2 PCV15 (VAXNEUVANCE^TM^)

VAXNEUVANCE (MERCK) is a licensed vaccine to be used in the UK. It is indicated for active immunization for the prevention of invasive disease caused by Streptococcus pneumoniae serotypes 1, 3, 4, 5, 6A, 6B, 7F, 9V, 14, 18C, 19A, 19F, 22F, 23F, and 33F in individuals 6 weeks of age and older.

One dose (0.5 ml) will be injected at each vaccination.

## 10.1.3 PCV20 (APEXXNAR/PREVENAR20®)

PCV20 Prevenar 20 (Pfizer), previously known as APEXXNAR, is a pneumococcal vaccine licensed to be used for infants and children 6 weeks to 18 years of age as well as adults 18 years of age and older, to help prevent diseases caused by Streptococcus pneumoniae serotypes 1, 3, 4, 5, 6A, 6B, 7F, 8, 9V, 10A, 11A, 12F, 14, 15B, 18C, 19A, 19F, 22F, 23F, and 33F.

## 10.2 Storage of IMP

Vaccines will be stored in fridges at 2 to 8 degrees Celsius at each study site or in hospital pharmacies, according to local arrangements. All fridges will be temperature monitored.

Vaccines will be transported to participants homes or to a suitable and convenient location in ‘cool boxes’ that are able to maintain temperatures between 2 and 8 degrees Celsius with a temperature monitor.

## 10.3 Compliance with trial treatment

Parents/legal guardians will need to ensure availability and access for study visits, to comply with the study. All vaccines in the study will be administered by trained study staff.

## 10.4 Accountability for IMPs

Accountability will be documented according to local policy and final vaccine accountability will be recorded.

PCV13 will be obtained as part of the routine supply from the UK national immunisation programs.

While PCV15 and PCV20 will be obtained directly from the manufacturers.

## 10.5 Concomitant medication

There are no other specific contraindications or precautions for the use of concomitant medication.

Medications given following an AE will be recorded in the e-Diary.

## 10.6 Other Treatments (non-IMPs):

- **DTaP-IPV-Hib- Hep B**: Infanrix hexa® (GSK) / Vaxelis® (Sanofi): is diphtheria (D), tetanus (T), pertussis (acellular component) (Pa), hepatitis B (rDNA) (HBV), poliomyelitis (inactivated (IPV) and Haemophilus influenza type b (Hib) conjugated vaccine (adsorbed). The vaccine is indicated for primary and booster to prevent diseases described above and is licensed to be used in the European Union, in children between 6 weeks and 36 months of age. Infanrix-hexa will be administered intramuscular as a 3-dose primary (2, 3 and 4 months).
- **Men B:** Bexsero® (Novartis): a meningococcal serogroup B vaccine adsorbed rDNA vaccine. This vaccine is licensed in Europe and currently given as part of the UK routine schedule (2, 3 and 12 months).
- **Rotavirus:** Rotarix® (GSK): a live attenuated vaccine (RIX4414 strain) that is administered orally as a suspension in a pre-filled oral applicator. It protects against gastroenteritis caused by rotavirus. This vaccine is licensed and is part of UK routine schedule at 2 and 3 months.
- **MMR:** Priorix® (GSK) / MMRvaxPro (MSD): a live attenuated vaccine that protects against measles (Schwarz strain), mumps (RIT 4385 strain) and rubella (Wistar RA 27/3 strain). Is indicated for active immunisation in children more than 9 months of age, adolescents and adults, to prevent the diseases above described. This vaccine is licensed in Europe and is given in the UK as part of the routine schedule at 12 months and 18 months of age.
- **MMRV :** ProQuad® (MSD) / Priorix Tetra (GSK): a live attenuated vaccine indicated for simultaneous vaccination against measles, mumps, rubella and varicella in individuals from 12 months of age.

## 10.7 Storage of non-IMP

Vaccines will be stored in fridges at 2 to 8 degrees Celsius at each study site or in hospital pharmacies, according to local arrangements. All fridges will be temperature monitored.

Vaccines will be transported to participants homes or to a suitable and convenient location in ‘cool boxes’ that are able to maintain temperatures between 2 and 8 degrees Celsius with a temperature monitor.

## 10.8 Accountability for non-IMPs

Accountability will be documented according to local policy and final vaccine accountability will be recorded.

The non-IMPs will be obtained as part of the routine supply from the UK national immunisation programs or obtained from UK commercial stocks.

## 10.9 Post-trial treatment

There will be no provision of the IMP after the study has finished.

## 10.10 Other Interventions

There are no additional interventions other than those specified in this protocol.

# 11 SAFETY REPORTING

Safety reporting will be carried out in accordance with the Oxford Vaccine Centre SOP “Safety reporting for CTIMPs” (OVC005).

All adverse events (AEs), including those that result in a participant’s withdrawal from the study, will be followed up until a satisfactory resolution occurs, or until a non-study related causality is assigned (if the participant’s parent/ legal guardian consents to the child being followed up after withdrawal).

## 11.1 Adverse Events Definitions

| Adverse Event (AE) | Any untoward medical occurrence in a participant to whom a medicinal product has been administered, including occurrences which are not necessarily caused by or related to that product. |
| --- | --- |
| Adverse Reaction (AR) | An untoward and unintended response in a participant to an investigational medicinal product which is related to any dose administered to that participant.  The phrase "response to an investigational medicinal product" means that a causal relationship between a trial medication and an AE is at least a reasonable possibility, i.e., the relationship cannot be ruled out.  All cases judged by either the reporting medically qualified professional or the Sponsor as having a reasonable suspected causal relationship to the trial medication qualify as adverse reactions. |
| Serious Adverse Event (SAE) | A serious adverse event is any untoward medical occurrence that:   - results in death - is life-threatening - requires inpatient hospitalisation or prolongation of existing hospitalisation - results in persistent or significant disability/incapacity - consists of a congenital anomaly or birth defect.   Other ‘important medical events’ may also be considered serious if they jeopardise the participant or require an intervention to prevent one of the above consequences.  NOTE: The term "life-threatening" in the definition of "serious" refers to an event in which the participant was at risk of death at the time of the event; it does not refer to an event which hypothetically might have caused death if it were more severe. |
| Serious Adverse Reaction (SAR) | An adverse event that is both serious and, in the opinion of the reporting Investigator, believed with reasonable probability to be due to one of the trial treatments, based on the information provided. |

- NB: to avoid confusion or misunderstanding of the difference between the terms “serious” and “severe”, the following note of clarification is provided: “Severe” is often used to describe intensity of a specific event, which may be of relatively minor medical significance. “Seriousness” is the regulatory definition supplied above.

## 11.2 Safety reporting window

Safety reporting for the study will commence once the participant is consented. It will end following the final study visit (V5).

SAEs resulting in participant withdrawal will be recorded from point of consent (Visit 1).

## 11.3 Assessment of results outside normal parameters as AEs and SAEs

## 11.4 Clinical

Abnormal clinical findings from medical history or examination will be assessed regarding their clinical significance throughout the study. If an abnormal finding is deemed to be clinically significant, the parent/legal guardian will be informed, and they will be referred to their appropriate GP practice with their permission.

## 11.5 Assessment of Severity

Solicited AEs occurring during the safety window for the trial that are observed by the Investigator or reported by the participant, will be reported on the trial CRF and graded for severity.

## 11.5.1 Solicited events

## 11.5.2 Local reactions:

Local solicited reactions will be recorded in the e-Diary / back-up paper diary from day of vaccination until day 6 post vaccination by the parent/legal guardian and will include erythema, induration and swelling (Table 2). Tenderness (pain) at injection site will also be recorded and graded for severity, as shown in Table 4 Parents/legal guardians will be issued with a ruler to measure erythema, induration or swelling which will be categorised according to the table below:

**Table 2: Local reactions**

| **Symptoms** | **Grade** | **Definition** |
| --- | --- | --- |
| **Injection site reaction: erythema, induration or swelling** | 0 (none) | No reactions |
|  | 1 (mild) | 1 to ≤25mm |
|  | 2 (moderate) | 26 to ≤50mm |
|  | 3 (severe) | >50mm |

## 11.5.3 Systemic reactions

Solicited systemic reactions will be recorded in the e-Diary/ back-up diary card for the day of vaccination and 6 days following vaccination with any PCV vaccine (V1 for arm 3, V2 for arms 1, 2 and 4, V3 for arms 1r4, 2r4, 3 and 4r4 and V4 for all arms). These include change in temperature, feeding, lethargy, vomiting, diarrhoea and irritability/fussiness. Parents/legal guardians will grade severity according to Tables 3 and 4 below. Temperature will be recorded daily over this period.

## 11.5.4 Temperature

Parents/legal guardians will be asked to record the participant’s axillary temperature at around six hours post vaccine and daily for the 6 days following vaccination with any PCV vaccine (V1 for arm 3, V2 for arms 1, 2 and 4, V3 for arms 1r4, 2r4, 3 and 4r4, and V4 for all arms). The axillary temperature will also be measured if at any time, the parent/legal guardian feels that the participant may have developed a fever. Axillary temperature will be graded as follows:

**Table 3: Temperature Grading**

| **Temperature Grade** | **Temperature definition (degree Celsius)** |
| --- | --- |
| 0 (none) | < 37.6 |
| 1 (mild) | 37.6 – 38.0 |
| 2 (moderate) | 38.1 – 39.0 |
| 3 (severe) | 39.1 – 40.0 |
| 4 (potentially life-threatening) | >40.1 |

**Table 4: Grading of systemic reactions**

|  | **Grading of severity** | | |  |
| --- | --- | --- | --- | --- |
| **Solicited Reactions** | **Mild** | **Moderate** | **Severe** | |
| Tenderness (pain) at the injection site | Minor reaction to touch | Cries / protests on touch | Cries when limb is moved or spontaneously  painful | |
| Change in feeding/eating habit | Feeding / eating less than usual / no effect on normal activity | Feeding / eating less than usual with  an effect on normal activity | Not feeding / eating at all | |
| Drowsiness | Drowsiness easily tolerated | Drowsiness that interferes with normal activity | Drowsiness that prevents normal  activity | |
| Vomiting | 1-2 episodes without interfering with routine | Several episodes & cannot keep any food down | Frequent episodes & not feeding / eating at all | |
| Diarrhoea | Stools are more loose than normal | Frequent runny stools without much solid material | Multiple liquid stools without much solid material | |
| Irritability/fussiness | Crying more than usual / no effect on normal activity | Crying more than usual / interferes with normal activity | Crying that cannot be comforted / prevents normal activity | |

The investigator will assess the maximum intensity that occurred over the duration of the event for all solicited and unsolicited AEs and SAEs. The assessment will be based on the investigator’s clinical judgement. SAEs leading to participant withdrawal will be recorded over the duration of the study.

**Table 5 for assessing the relationship of vaccine administration to an AE**

| 0 | No relationship | No temporal relationship to study product and alternate aetiology (clinical state, environmental or other interventions) and  does not follow known pattern of response to study product |
| --- | --- | --- |
| 1 | Unlikely | Unlikely temporal relationship to study product and alternate aetiology likely (clinical state, environmental or other interventions) and does not follow known typical or plausible pattern of response to study product |
| 2 | Possible | Reasonable temporal relationship to study product or event not readily produced by clinical state, environmental or other interventions or similar pattern of response to that seen with other vaccines |
| 3 | Probable | Reasonable temporal relationship to study product and  event not readily produced by clinical state, environment, or other interventions or known pattern of response seen with other vaccines |
| 4 | Definite | Reasonable temporal relationship to study product ***and***  event not readily produced by clinical state, environment, or other interventions ***and***  known pattern of response seen with other vaccines |

AEs graded in categories 2 to 4 in the above scheme are regarded as having a reasonable suspected causal relationship to the administered vaccine.

## 11.6 Procedures for recording adverse events

## 11.6.1 Reporting of solicited AEs

Participants will be asked to record local and systemic AEs (solicited AEs) for the day of vaccination and 6 days following vaccination with any PCV vaccine (V1 for arm 3, V2 for arms 1, 2 and 4, V3 for arms 1r4, 2r4, 3 and 4r4, and V4 for all arms) (and longer if symptoms persist at day 7, until resolution or stabilisation) in the electronic/paper diary. Temperature will be recorded on the day of vaccination and for 6 days following receiving a dose of any PCV vaccine (V1 for arm 3, V2 for arms 1, 2 and 4, V3 for arms 1r4, 2r4, 3 and 4r4, and V4 for all arms).

## 11.6.2 Reporting of unsolicited AEs

All local and systemic unsolicited AEs occurring in the 28 days following receiving any PCV vaccine (V1 for arm 3, V2 for arms 1, 2 and 4, V3 for arms 1r4, 2r4, 3 and 4r4, and V4 for all arms) observed by the Investigator or reported by the participant (regardless of whether attributed to study medication) will be recorded in the CRF. Serious adverse events occurring at any time over the duration of the study (*i.e.,* from enrolment, whilst the individual participant remains in the study) will also be recorded. The record should include the following: description; date of onset and end date; severity; assessment of relatedness to trial medication.

The severity of events will be assessed on the following scale: 0=none, 1=mild, 2=moderate, 3=severe. Guidelines for scoring of solicited local and systemic reactions are detailed above in Tables 2, 3, 4 and 5.

AEs considered related to the trial medication, as judged by a medically qualified investigator or the Sponsor, will be followed either until resolution or the event is considered stable.

It will be left to the Investigator’s clinical judgment to decide whether an AE is of sufficient severity to require the participant’s removal from treatment. The parents/legal guardians of participants may also voluntarily withdraw from treatment due to what they perceive as an intolerable AE. If either of these occurs, the participant must undergo an end of trial assessment and be given appropriate care under medical supervision until symptoms cease, or the condition becomes stable.

## 11.7 Reporting procedures for Serious adverse events:

All SAEs will be reported to the Investigators by study team members, who will complete the electronic SAE reporting form. Alternatively, a backup paper reporting form will be available; if this is used, it should be sent by email to the Chief Investigator (as the Sponsor’s representative). This will be done immediately and at the latest within 24 hours of the site study team becoming aware of the event. The SAE reporter should ensure, and document, that receipt of the form is acknowledged.

The CI, or a delegate within OVG, will perform an initial check of the report and request any additional information. Additional and further requested information (follow-up or corrections to the original case) will be detailed on a new electronic SAE reporting form; alternatively, the backup paper reporting form may be completed and emailed to the CI (or delegate within OVG).

## 11.8 Expectedness

Expectedness will be determined according to the approved Reference Safety Information (RSI) listed in Section 4.8 (undesirable effects) of the Summary of Product Characteristics. The local PI will assess relatedness, but the CI or delegate will assess expectedness (as delegated by the Sponsor to the CI). The SmPCs relevant to this study are listed in Table 6.

**Table 6: Summary of Product Characteristics**

## Prevenar 13 suspension for injection^1^

## Prevenar 20 suspension for injection in pre-filled syringe^2^

Vaxneuvance suspension for injection in pre-filled syringe**^3^**

Vaxelis suspension for injection in pre-filled syringe^16^

## Infanrix hexa, Powder and suspension for suspension for injection^4^

Bexsero suspension for injection in pre-filled syringe**^5^**

Rotarix oral suspension in pre-filled oral applicator**^6^**

M-M-RvaxPro powder and solvent for suspension for injection^17^

Priorix Powder and solvent for solution for injection in a pre-filled syringe**^8^**

ProQuad powder and solvent for suspension for injection in a pre-filled syringe^19^

Priorix-Tetra powder and solvent for solution for injection in pre-filled syringe^20^

## 11.9 SUSAR reporting

All SUSARs will be reported by the sponsor delegate to the relevant Competent Authority and to the REC and other parties, as applicable. For fatal and life-threatening SUSARS, this will be done no later than 7 calendar days after the Sponsor or delegate is first aware of the reaction. Any additional relevant information will be reported within 8 calendar days of the initial report. All other SUSARs will be reported within 15 calendar days.

## 11.10 Development Safety Update Reports

The Oxford Vaccine Group will submit DSURs for following vaccines: PCV13, PCV15 and PCV20 once a year throughout the clinical trial, or on request to the Competent Authority (MHRA in the UK), Ethics Committee, HRA (where required), Host NHS Trust and Sponsor.

# 12 STATISTICS

## 12.1 Sample size

The primary analysis of this study will be a non-inferiority comparison between homologous PCV15/PCV20 schedules and the homologous PCV13 (as the reference).

Based on the published data, the standard deviations of IgG concentrations at log10 scale for majority of the PCV13 serotypes are below 0.5. Assuming the SD of 0.5, the attrition rate of 10-15%, and the non-inferiority margin of 0.67 (or -0.174 at log10 scale), the study will need to recruit an effective sample size of 131 per arm at the first randomisation. After accounting for the attrition rate, a total sample size of 600 to claim non-inferiority at the significant level of two-sided 0.05 with 80% power. Given there will be 13 serotypes and two comparisons for PCV15 and PCV20, the sample size calculation is not adjusted for multiple comparisons. This means that if the homologous PCV15 and PCV20 schedules are truly non-inferior to PCV13, it is expected that non-inferiority will not be met for all comparisons (by chance 1 in 5 will not meet non-inferiority in the worst scenario as we used a conservative SD for all serotypes). On the other hand, if the homologous PCV15 and PCV20 schedules are truly inferior to PCV13, it is expected that one non-inferiority will be falsely claimed for every 40 comparisons. The conclusion of non-inferiority between PCV15/PCV20 and PCV13 will not be based on the comparison of one single serotype and all the 13 serotypes will be considered when interpreting the results.

## 12.2 Description of statistical methods

The primary outcome is the PCV13 serotype-specific IgG one month after the booster dose. The geometric mean concentrations (GMC) of IgG will be compared between homologous PCV15 & PCV20 arms and homologous PCV13 arm (as the reference) under the hypothesis:

H0: GMC_PCV15/PCV20_ / GMC_PCV13_ ≤ 0.67 or 𝑙𝑜𝑔10(GMC_PCV15/PCV20_) - 𝑙𝑜𝑔10(GMC_PCV13_) ≤ -0.174;

H1: GMC_PCV15/PCV20_ / GMC_PCV13_ > 0.67 or 𝑙𝑜𝑔10(GMC_PCV15/PCV20_) - 𝑙𝑜𝑔10(GMC_PCV13_)> -0.174.

The PCV13 serotype-specific IgG concentration will be transformed using logarithmic transformations (base 10) to render a normal distribution. We will test the above hypothesis using the multiple regression on 𝑙𝑜𝑔10GMC adjusting for randomisation design variables, and the pre-specified prognostic factors, if any. The adjusted mean difference of 𝑙𝑜𝑔10GMC will be presented with the two-sided 95% confidence interval (CI). We will claim homologous PCV15/PCV20 arms are non-inferior to homologous PCV13 arm if the lower CI lies above -0. 174.

The primary analysis will be conducted on a per-protocol basis among participants whose primary endpoint at D28 post boost is available, as the intent to treat analysis is no longer producing a conservative estimation in non-inferiority trials. The modified intent to treat analysis will also be conducted as a sensitivity analysis. The primary analysis will be carried out when the primary endpoint of D28 post boost anti-spike IgG data become available.

A fully detailed statistical analysis plan will be prepared and will be signed off by the Chief Investigator prior to conducting any data analyses.

## 12.3 Missing data

The level and pattern of the missing data in the baseline variables will be reported. The potential causes of any missing data will be investigated and documented as far as possible. Any missing data will be dealt with, if needed, using methods appropriate to the conjectured missing mechanism and level of missing.

## 12.4 Interim analyses

There will be no interim analysis.

# 13 DATA MANAGEMENT

## 13.1 Source data

Source documents are where data are first recorded, and from which participants’ CRF data are obtained. These include, but are not limited to, hospital records (from which medical history and previous and concurrent medication may be summarised into the CRF), clinical and office charts, laboratory and pharmacy records, vaccination history, diaries, microfiches, radiographs, and correspondence.

CRF entries will be considered source data if the CRF is the site of the original recording (e.g., if there is no other written or electronic record of data). All documents will be stored safely in confidential locations. On all trial-specific documents, other than the signed consent, the participant will be referred to by the trial participant number/code, not by name.

## 13.2 Access to data

Direct access will be granted to authorised representatives from the Sponsor, host institution and the regulatory authorities to permit trial-related monitoring, audits and inspections. Access to medical records by those outside the direct healthcare team will only occur if consent has been received. This would be for the purpose of clarifying medical history in relation to the inclusion/exclusion criteria and safety reporting.

## 13.3 Data Recording and Record Keeping

CRF data will be recorded directly into an electronic data capture (EDC) system (REDCap) or onto a paper source document for later entry into the EDC, if direct entry is not available. Any additional information that needs recording but is not relevant for the CRF (such as sites for venepuncture, parental availability, etc.) will be recorded on a separate paper source document. All documents will be stored safely in confidential conditions.

Parents/legal guardians will consent for email addresses to be entered into an electronic diary system. This enables the diary to be sent to them for completion online. The EDC systems (electronic diary and CRF data) use a MySQL database via a secure web interface with data checks used during data entry to ensure data quality. The EDC database includes a complete suite of features which are compliant with EU and UK regulations and NHS security policies, including a full audit trail, user-based privileges, and integration with the institutional LDAP (Lightweight Directory Access Protocol) server. The MySQL database and the web server will both be housed on secure servers operated by the University of Oxford IT Services. The servers are in a physically secure location in Europe and are backed up in Europe, with the back-ups stored in accordance with the IT department schedule of daily, weekly, and monthly tapes retained for 1 month, 3 months, and 6 months, respectively. Weekly back-up tapes are stored offsite. The IT servers provide a stable, secure, well-maintained, and high-capacity data storage environment. REDCap and MySQL are widely used, powerful, reliable, well-supported systems. Access to the study's database and the diary will be restricted to the members of the study team by username and password.

All entries made to the source documents should be printed legibly. If entry errors have been made, they should be corrected according to GCP. Information recorded in the source document must be subsequently transferred onto the database by the site collecting the data. The participants will be identified by a unique study specific number in any of the used databases. Participant's personal information will be stored on a separate database not linked to the clinical database, except the electronic diaries, for which consent will be obtained to store the parent/ legal guardian’s email address required for the system to function. Only site research staff and sponsor data managers will have access to view the email address.

The principal investigator at each study site must make appropriate arrangements to store the essential study documents (as defined in Essential Documents for the Conduct of a Clinical Trial, International Conference on Harmonisation (ICH) E6, Guideline for Good Clinical Practice), including the Investigator Site File. Copies of all study documents will be retained after the completion or discontinuation of the study for 3 years after the youngest participant turns 18 years. In addition, the investigator is responsible for archiving all relevant source documents, so that the study data can be compared against source data after completion of the study (e.g., in case of inspection from authorities). The investigator is required to ensure the continued storage of the documents, even if the investigator, for example, leaves the clinic/practice or retires before the end of required storage period. Delegation of this transfer of responsibility to their successor must be documented in writing.

# 14 QUALITY ASSURANCE PROCEDURES

The trial will be conducted in accordance with the current approved protocol, GCP, relevant regulations and standard operating procedures

## 14.1 Monitoring

Monitoring of study conduct and protocol compliance will be delegated by the sponsor to the Oxford Vaccine Group. Regular monitoring will be performed by the OVG Quality Assurance team, with details outlined in a study monitoring plan. Data will be evaluated for compliance with the protocol and accuracy in relation to the source documents. Following the monitoring plan, the monitors will verify that the clinical trial is conducted, and that data are generated, documented and reported, in compliance with the protocol, GCP and the applicable regulatory requirements.

# 15 PROTOCOL DEVIATION

A trial related deviation is a departure from the ethically approved trial protocol or other trial document or process *(e.g.*, consent process or IMP administration) or from Good Clinical Practice (GCP) or any applicable regulatory requirements. Any deviations from the protocol will be documented in a protocol deviation form and filed in the trial master file. Each deviation will be assessed as to its impact on participant’s safety, study conduct and data integrity. Significant deviations will be listed in the end of study report.

# 16 SERIOUS BREACHES

The Medicines for Human Use (Clinical Trials) Regulations contain a requirement for the notification of "serious breaches" to the MHRA within 7 days of the Sponsor becoming aware of the breach.

A serious breach is defined as “A breach of GCP or the trial protocol which is likely to affect to a significant degree:

(a) the safety, or physical or mental integrity of the subjects of the trial; or

(b) the scientific value of the trial”.

If a serious breach is suspected, the Sponsor must be contacted within one working day. In collaboration with the CI, the serious breach will be reviewed by the Sponsor and, if appropriate, the Sponsor will report it to the REC committee, Regulatory Authority and the NHS host organisation within seven calendar days.

# 17 ETHICAL AND REGULATORY CONSIDERATIONS

## 17.1 Declaration of Helsinki

The Investigator will ensure that this trial is conducted in accordance with the principles of the Declaration of Helsinki.

## 17.2 Guidelines of Good Clinical Practice

The Investigator will ensure that this trial is conducted in accordance with relevant regulations and with Good Clinical Practice.

## 17.3 Approvals

The protocol, informed consent form, study information booklet, advertising material and any other supporting trial documents will be submitted to an appropriate Research Ethics Committee (REC), HRA (if relevant), regulatory authorities (MHRA in the UK), and host institution(s) for written approval.

The Investigator will submit and, where necessary, obtain approval from the above parties for all substantial amendments to the original approved documents.

## 17.4 Other Ethical Consideration

Venepuncture may be uncomfortable, but every effort will be made to reduce any discomfort associated with blood sampling. All procedures will be performed by fully trained nurses or doctors who are experienced in vaccinating and obtaining blood samples from young infants.

Any participant found to have a low antibody response against all PCV serotypes in the post booster blood (as judged by all PCV13 serotype antibodies being <0.35 microgram/mL), taken at 13 months of age, will be contacted to discuss the results and will be referred for further investigation with their permission.

## 17.5 Reporting

The CI will submit once a year throughout the clinical trial, or on request, an Annual Progress Report to the REC, HRA (where required), host organisation, Sponsor and the funder.

In addition, an End of Trial notification and final report will be submitted to the MHRA, the REC, host organisation and Sponsor.

## 17.6 Participant confidentiality

The study staff will ensure that the participants’ confidentiality is maintained. Each participant will be identified only by a participant ID number on all study documents and any electronic database, except for the CRF (where participant initials may be added) and the signed consent form. All documents will be stored securely and will be only accessible by study staff and authorised personnel. Parent/ legal guardian’s email address is required for the electronic diaries, for the system to function. Only relevant site research staff and sponsor data managers have access to view the email address. The study will comply with the General Data Protection Regulation (GDPR), including the requirement for data to be anonymised as soon as it is practical to do so.

## 17.7 Expense and benefits

If study visits occur in the participant’s homes, reimbursement will not be provided. If the parents/ legal guardians need to travel with the participant to attend appointments at a convenient hospital or study site location, they will be reimbursed for their travel expenses at a flat rate of £25 per visit attended via bank transfer.

If the parent/legal guardian withdraws consent for their child's continued participation in the trial or is withdrawn for any other reason, they will still be compensated for any trial visits they attended in a clinical setting.

# 18 FINANCE AND INSURANCE

## 18.1 Funding

This study will be funded by the NIHR under the NISECII consortium.

## 18.2 Insurance

The University has a specialist insurance policy in place which would operate in the event of any participant suffering harm consequent to their involvement in the research (Newline Underwriting Management Ltd, at Lloyd’s of London). NHS indemnity operates in respect of the clinical treatment that is provided.

# 19 PUBLICATION POLICY

The Investigators will co-ordinate the dissemination of the data from this study, including the study participants. All publications (e.g., manuscripts, abstracts, oral/slide presentations, book chapters) based on this study will be reviewed by all investigators prior to submission. Authors will acknowledge that the study was funded by NISEC. Authorship will be determined in accordance with the ICMJE (International Committee of Medical Journal Editors) guidelines and other contributors will be acknowledged.

# 20 ARCHIVING

Study data may be stored electronically on a secure server, and paper notes will be kept in a secure location at the site. All essential documents will be retained for up to 25 years, or as per national regulatory requirements. Pseudo-anonymised research data may be stored indefinitely, but with 5 yearly reviews. General archiving procedures will be conducted in compliance to SOP OVC020 Archiving.

# 21 SCHEDULE OF EVENTS

| Visit Number | Pre-V1^2^  *Remote* | V1 | V2 | V3 | V3 (A) | V4 | V5 | Additional Visit† |
| --- | --- | --- | --- | --- | --- | --- | --- | --- |
| Approx. age in months |  | 2 months^1^ | 3 months^1^ | 4 months^1^ | 5 months^1^ | 12 months | 13 months^1^ | 3 – 4 months^1^ |
| Visit Window |  | Aged 8 – 10 weeks | 28 – 42 days post V1 | 28 – 42 days post V2 | 28 – 42 days post V3 | 12 months of age + 14 days | 28 – 42 days post V4 |  |
| Online Screening Questionnaire | X |  |  |  |  |  |  |  |
| Informed consent |  | X |  |  |  |  |  | X |
| Review of inclusion and exclusion criteria | X | X | X | X | X | X | X |  |
| Medical history |  | X |  |  |  |  |  | X |
| Temperature |  | X | X | X |  | X |  |  |
| Physical examination ^3^ |  | X | X | X | X | X | X |  |
| Randomisation | X |  |  |  |  |  |  |  |
| AE Collection/Review ^4^ |  | X | X | X | X | X | X |  |
| SAEs Collection/Review |  | X | X | X | X | X | X |  |
| Arms 1, 2, 4 | | | | | | | |  |
| Routine vaccination |  | 6 in 1  Rotavirus  MenB | 6 in 1  Rotavirus  MenB | 6 in 1  MenB* |  | MMR or MMRV  MenB |  | MenB* |
| PCV vaccination^5^ |  |  | X |  |  | X |  |  |
| Diary Issue |  |  | X |  |  | X |  |  |
| Diary Review^6^ |  |  |  | X |  |  | X |  |
| Blood |  |  |  | X |  |  | X |  |
| MLF |  |  | X | X |  |  | X |  |
| Arms 1r4,2r4, 4r4 | | | | | | | |  |
| Routine vaccination |  | 6 in 1  Rotavirus  MenB | 6 in 1  Rotavirus  MenB | 6 in 1 |  | MMR or MMRV  MenB |  |  |
| PCV vaccination^5^ |  |  |  | X |  | X |  |  |
| Diary Issue |  |  |  | X |  | X |  |  |
| Diary Review^6^ |  |  |  |  | X |  | X |  |
| Blood |  |  |  |  | X |  | X |  |
| MLF |  |  |  | X | X |  | X |  |
| Arm 3 | | | | | | | |  |
| Routine vaccination |  | 6 in 1  Rotavirus  MenB | 6 in 1  Rotavirus  MenB | 6 in 1  MenB* |  | MMR or MMRV  MenB |  | MenB* |
| PCV vaccination^5^ |  | X |  | X |  | X |  |  |
| Diary Issue |  | X |  | X |  | X |  |  |
| Diary Review^6^ |  |  | X |  | X |  | X |  |
| Blood |  |  |  |  | X |  | X |  |
| MLF |  | X | X |  | X |  | X |  |

*2nd dose of MenB to be given at visit 3 or Additional visit for participant who already had their visit 2 prior to the implementation of SA04)

^1^Month will be defined as 28 days ^2^Visit number pre-V1 will be remote (telephone/email) ^3^If required ^4^AEs are only collected for 28 days following PCV vaccination ^5^According to randomisation ^6^Include review and collection of paper diary if completed

† Optional, as required

# 22 REFERENCES

1.	emc. Prevenar 13 - Summary of product characteristics. 12 Oct 2021.

<https://www.medicines.org.uk/emc/product/453>

2.	EMA. Prevenar 20 (previously Apexxnar) - Summary of product characteristics. 24 Sept 2024

<https://www.medicines.org.uk/emc/product/13461>

3.	emc. Vaxneuvance - Summary of product characteristics. 04 July 2023.

<https://www.medicines.org.uk/emc/product/13754>

4.	emc. Infanrix hexa - Summary of product characteristics. 04 Sep 2023.

<https://www.medicines.org.uk/emc/product/2586>

5.	emc. Bexsero Meningococcal- Summary of product characteristics. 01 Aug 2023.

<https://www.medicines.org.uk/emc/product/5168>

6.	emc. Rotarix - Summary of product characteristics. 31 Oct 2022.

<https://www.medicines.org.uk/emc/product/2739>

7.	emc. Priorix - Summary of product characteristics. 20 May 2022.

<https://www.medicines.org.uk/emc/product/1159>

8.	Méroc, E., Fletcher, M. A., Hanquet, G., Slack, M. P. E., Baay, M., Hayford, K., Gessner, B. D., & Grant, L. R. (2023). Systematic Literature Review of the Epidemiological Characteristics of Pneumococcal Disease Caused by the Additional Serotypes Covered by the 20-Valent Pneumococcal Conjugate Vaccine. Microorganisms, 11(7), 1816. <https://doi.org/10.3390/microorganisms11071816>

(Méroc et al., 2023)

9.	Janssens, E., Flamaing, J., Vandermeulen, C., Peetermans, W. E., Desmet, S., & De Munter, P. (2023). The 20-valent pneumococcal conjugate vaccine (PCV20): expected added value. *Acta clinica Belgica*, *78*(1), 78–86. <https://doi.org/10.1080/17843286.2022.2039865>

(Janssens et al., 2022)

10.	Joint Committee on Vaccination and Immunisation (JCVI). Minute of the meeting held on 01^st^ February 2023. <https://app.box.com/s/iddfb4ppwkmtjusir2tc/file/1165597788909>

11.	Centers for Disease Control and Prevention. Recommendations for Use of 20-Valent Pneumococcal Conjugate Vaccine in Children ― United States, 2023. 23 Sep 2023. <https://www.cdc.gov/mmwr/volumes/72/wr/mm7239a5.htm>

12.	Ladhani, S. N., Collins, S., Djennad, A., Sheppard, C. L., Borrow, R., Fry, N. K., … Ramsay, M. E. (2018). Rapid increase in non-vaccine serotypes causing invasive pneumococcal disease in England and Wales, 2000–17: a prospective national observational cohort study. The Lancet Infectious Diseases, 18(4), 441–451. <https://doi.org/10.1016/S1473-3099(18)30052-5>(Ladhani et al., 2018)

13.	Goldblatt, D., Southern, J., Andrews, N. J., Burbidge, P., Partington, J., Roalfe, L., Valente Pinto, M., Thalasselis, V., Plested, E., Richardson, H., Snape, M. D., &amp; Miller, E. (2018). Pneumococcal conjugate vaccine 13 delivered as one primary and one booster dose (1 + 1) compared with two primary doses and a booster (2 + 1) in UK infants: A Multicentre, parallel group randomised controlled trial. The Lancet Infectious Diseases, 18(2), 171–179. <https://doi.org/10.1016/s1473-3099(17)30654-0> (Goldblatt et al., 2018)

14. Tin Tin Htar, M., Christopoulou, D., &amp; Schmitt, H.-J. (2015). Pneumococcal serotype evolution in Western Europe. BMC Infectious Diseases, 15(1). <https://doi.org/10.1186/s12879-015-1147-x>

(Tin Tin Htar et al., 2015)

15. emc. Vaxelis - Summary of product characteristics. 25 January 2023. <https://www.medicines.org.uk/emc/product/12264/smpc#gref>.

16. emc. M-M-RvaxPro - Summary of product characteristics. 26 July 2022. <https://www.medicines.org.uk/emc/product/6307/smpc#gref>.

17. Senders S, Klein NP, Lamberth E, Thompson A, Drozd J, Trammel J, PengY,Giardina PC, Jansen KU, Gruber WC, Scott DA, Watson W. Safety and Immunogenicity of a 20-valent Pneumococcal Conjugate Vaccine in Healthy Infants in the United States. Pediatr Infect Dis J. 2021 Oct 1;40(10):944-951. doi: 10.1097/INF.0000000000003277. PMID: 34525007; PMCID: PMC8443440.

18. <https://vaccine.ac.uk/research/lion-menb/>

19. emc. ProQuad – Summary of product characteristics. 01 October 2025. https://www.medicines.org.uk/emc/product/101444/smpc

20. emc. Priorix Tetra – Summary of product characteristics. 07 April 2025. https://www.medicines.org.uk/emc/product/2586/smpc

# APPENDIX A: AMENDMENT HISTORY

| **Amendment**  **No.** | **Protocol**  **Version No.** | **Date issued** | **Author(s) of changes** | **Details of Changes made** |
| --- | --- | --- | --- | --- |
| Amendment 01 – SA01 | 1.1 | 02 Sep 2024 | Denis Murphy | Changes to the protocol:   - Table 1 moved to section 6 - At section 8.2 the text is changed to reflect the revised screening questionnaire and a sentence added to state that if the screening questionnaire is not completed there will be a discussion by phone or email - Random block sizes to be used changed from 2 or 4 to 4 or 8, section 8.4 - The sentence “The randomisation lists will be stratified by study site” is added at section 8.4 - At section 9.1 the sentence at the third bullet point "Obtain written informed consent from the parent/guardian for storage of any leftover clinical samples into Biobank" is replaced by the sentence "Give a brief explanation of biobank and leave a study booklet for biobank for parent/guardian" At section 9.1 the bullet point “Physical examination (if required)” is amended to read “Physical examination Must be performed if the 6-8 week health development review has not been performed.At investigator discretion if indicated by medical history - At section 9.1 antipyretic use is removed from recording in e-diary/diary card - The sentence “Review medical history and record any new concomitant medications for all arms” is included at sections 9.2, 9.3, 9.4 and 9.5 - At section 9.2 the sentence "Obtain written informed consent from the parent/guardian for storage of any leftover clinical samples into Biobank" is added, the bullet point "Record and report any SAEs that have occurred since the last visit for arm 3" is deleted, AEs are included in the fifth bullet point and the bullet points "Review e-Diary/diary card for arm 3" and "Review medical history and record any new concomitant medications for arm 3" are moved up the list of procedures to appear before the vaccination step - The sentence "Obtain written informed consent from the parent/guardian for storage of any leftover clinical samples into Biobank. (if not consented at previous visit)" is included at sections 9.3, 9.4, 9.5, 9.6 - At section 9.3, AEs are included in the fourth bullet point and the sentence "Record and report any SAE that have occurred since last visit for arm 3" is added - At section 9.4 AEs are included in the fourth bullet point and final bullet point a maximum volume of 5ml blood is to be collected (not a maximum volume of 6ml) - At section 9.5, arm 3 is included within the third bullet point - At section 9.5 AEs are included in the fourth bullet point - At sections 9.2, 9.3, 9.4, 9.5 and 9.6, text is added to that any new concomitant medications are only recorded if administered for AEs/SAEs - At section 9.9, period for recording all solicited reactions form the day of vaccination is reduced from seven to six days, this change is a correction as first day of vaccination is day zero so collecting data for up to day six gives seven days of data collection - At section 9.11, the definition of point of enrolment is amended to read "The point of enrolment is defined as when the infant is randomised into the study" - Sections 9.12, 9.13, text is moved from section 9.13 to section 9.12 - At section 10.5 the text is revised so that medications given following an AE will be recorded in the e-diary (previously recording period was seven days) and recording of concomitant medication removed - At section 18.1 the funder is clarified - At section 19 the sentence "Further details of funder's review prior to publication will be outlined in the Clinical Trials Agreement" is deleted - At section 21, Schedule of Events, temperature recording is deleted from V5, biobank consent is deleted from V1 and added for subsequent visits, the collection of adverse events and serious adverse event are separated as different activities and the administration of the PCV13, PCV15 and PCV20 vaccines are recorded as separate activities. |
| Amendment 2 - SA02 | 2.0 | 18 Sep 24 | Alix de Calignon | - References to Apexxnar are replaced by Apexxnar/Prevenar 20 throughout the protocol - The following text is added at Table 2 Synopsis: “Prevenar 20 (formerly Apexxnar) and Apexxnar are the same vaccines with different names for different regions” - In Section 4 the licensing information for PCV20 has been updated. - In section 7.3, the formatting of one exclusion criteria is modified. - In section 9.5, the sequence of words is changed in two sentences regarding the areas of vaccine injection. - In section 10.1, an update reflecting the name and licensing changes for PCV20 was added - In section 22, the new version of the SmPC for Prevenar 20 is replacing the previous version. |
| Amendment 3- SA03 | 3.0 | 16 May 25 | Denis Murphy, Riffat Aslam, Xinxue Liu, Reuben Bennett, Alix de Calignon | - The study title was amended to recognise that in homologous Arms 1, 2, and 4 the PCV may be given at 3/4 months and 12 months. - The protocol was revised throughout so that references to Arms 1/1r4, 2/2r4 and 4/4r4 are included (as opposed to solely Arms 1, 2 and 4). - The protocol was revised throughout so that references to the Hib/MenC vaccine (Menitorix) were removed - In Synopsis Table: Objective was clarified with measurement of PCV20 specific IgG in all study arms. Exploratory Objective was added outlining assessment of PCV immune titres in groups A (PCV vaccine at 3 and 12 months of age) to groups B (4 and 12 months of age) post prime and post boost. - In section 4, some text is added setting out the current UK immunization schedule of two doses of the PCV13 vaccine at ages 3 and 12 months and the findings of the Lion MenB study - In section 4 text was added explaining that Hib/MenC vaccine (Menitorix) was removed from the routine childhood vaccination program - Addition of exploratory objective to section 5.0 - comparing PCV antibody titres of infants with PCV vaccination at 3 and 12 months to infants receiving PCV vaccination at 4 and 12 months. - In section 6, some text and a table were added to explain the schedule of vaccines and sampling for the new arms 1r4, 2r4 and 4r4. - Sections 8.4 and 9 were revised to give information regarding second randomisation 1:1 of groups 1, 2 and 4 into 1/1r4, 2/2r4 and 4/4r4. - Study visits in section 9 were revised to allow for Arms 1r4, 2r4 and 4r4 and associated revised study procedures and sampling. - The Schedule of Events was amended to include Arms 1r4, 2r4 and 4r4 |
| Non-Substantial amendment 05 | 3.1 | 26 June 2025 | Alix de Calignon | - In the Synopsis section, the planned trial period was extended from 18 months to 30 months. |
| Non-Substantial amendment 06 | 3.2 | 27 June 2025 | Alix de Calignon | - A typo was corrected for the window for V2, V3, V3A and V5. The window is now 28-42 days instead of 28-41 days. This change is reflected in the Table of Content, the Study Visits section and the Schedule of events. |
| Non-substantial amendment 07 | 3.3 | 15 July 2025 | Denis Murphy | - Changes made to reflect updated UK immunization schedule as of 01 July 25 |
| Amendment 4- SA04 | 4.0 | 25 July 2025 | Riffat Aslam | - All arms will receive MenB vaccine at 2 month and 3 month visit to reflect the changes made to infant immunization schedule as of 01 July 2025. - Additional visit added to allow MenB vaccine to be given between 2 month and 3 month visits, to avoid unnecessary delay in vaccination. - Vaccination administration sites for 2 and 3 months visit has been changed to ensure MenB vaccine is given in a separate limb (reflecting the best practice). |
|  | 4.1 | 06 Aug 2025 | Hannah Robinson | - Clarification that the Additional visit is optional, to be used for preventing delay in MenB vaccination if required - Clarification that the 2nd MenB vaccination may be given at 3-months, 4-months or at the Additional visit. |
|  | 5.0 | 02 Oct 2025 | Alix de Calignon | - Changes made to reflect upcoming updated UK immunisation schedule with the possible inclusion of the MMRV vaccine to be administered once the national immunisation schedule is updated. - Formatting errors were corrected throughout the document, including in the table of content. - V4 in tables 1, 1.1 and schedule of events clarified so that 12 months refers to age of infants (and not 12x28 days). |

Protocol amendments must be submitted to the Sponsor for approval prior to submission to the REC committee or MHRA.
