# Supplementary material for: Open-label, randomised controlled, multicentre study evaluating higher valency pneumococcal vaccines (PCV15/PCV20) compared with PCV13 given in homologous schedules at 3 months and 12 months (‘1+1’ schedule) and 2 months, 4 months and 12 months (‘2+1’ schedule) in infants: protocol
Source: BMJ Open. 2026 Sep 22;16(9):e116062. doi: 10.1136/bmjopen-2025-116062 (PMC13599847; doi:10.1136/bmjopen-2025-116062)
Supplement: Supplementary file 7 [file bmjopen-16-9-s006.docx]

1.1

Study Document

Dominic Kelly

N/A

unknown

|  | Oxford Vaccine Group University of Oxford Centre for Clinical Vaccinology and Tropical Medicine, Churchill Hospital, Headington, Oxford OX3 7LE Telephone: 01865 611400 [info@ovg.ox.ac.uk](mailto:info@ovg.ox.ac.uk) [www.ovg.ox.ac.uk](http://www.ovg.ox.ac.uk) |  |
| --- | --- | --- |

Document Category: Study Plan

Code:	OVG 2102

Title:	PCV15-20 Monitoring plan

Version:

Document Type:

Authorised By:

Number Of Copies: Location Of Copy:

PCV15-20 Monitoring plan - Version: 1.1. Index: OVG 2102. Printed: 26-Jun-2025 09:24

Monitoring Plan

Study: Evaluation of higher valency pneumococcal vaccines (PCV15/PCV20) compared to PCV13

given in homologous schedules at 3 months and 12 months (“1+1” schedule) and 2 months, 4

months and 12 months (“2+1” schedule) in infants.

OVG reference: OVG2023/10

IRAS Project ID: 1009039

Chief Investigator: Dr. Dominic Kelly

**1.	INTRODUCTION**

The purpose of this document is to describe the provision of monitoring by Oxford Vaccine Group monitors

for the following study:

Evaluation of higher valency pneumococcal vaccines (PCV15/PCV20) compared to PCV13 given in homologous schedules at 3/4 months and 12 months (“1+1” schedule) and 2 months, 4 months and 12 months (“2+1” schedule) in infants

The purpose of monitoring is to ensure that:

- The rights and wellbeing of human subjects are being protected,
- The conduct of the study is in compliance with the protocol, regulatory requirements and the principles pf GCP.

PCV15-20 Monitoring plan - Version: 1.1. Index: OVG 2102. Printed: 26-Jun-2025 09:24

**2.	STUDY DETAILS**

This is a Phase IV Open label, randomised, non-inferiority, comparative study

The planned trial period is approximately 18 months.
The trial will be conducted at the following sites:

**1.Oxford**

Professor Dominic Kelly

**2.Bristol**

Dr Marion Roderick

**3.Nottingham**

Dr David Turner

Monitoring Plan

Study: Evaluation of higher valency pneumococcal vaccines (PCV15/PCV20) compared to PCV13

given in homologous schedules at 3 months and 12 months (“1+1” schedule) and 2 months, 4

months and 12 months (“2+1” schedule) in infants.

OVG reference: OVG2023/10

IRAS Project ID: 1009039

Chief Investigator: Dr. Dominic Kelly

**4.St Georges**

Prof. Paul Heath

**5.Southampton**

Dr Daniel Owens

PCV15-20 Monitoring plan - Version: 1.1. Index: OVG 2102. Printed: 26-Jun-2025 09:24

**6.Liverpool**

Dr Dan Hawcutt

Monitoring Plan

Study: Evaluation of higher valency pneumococcal vaccines (PCV15/PCV20) compared to PCV13

given in homologous schedules at 3 months and 12 months (“1+1” schedule) and 2 months, 4

months and 12 months (“2+1” schedule) in infants.

OVG reference: OVG2023/10

IRAS Project ID: 1009039

Chief Investigator: Dr. Dominic Kelly

**Table 1 Group allocation**

Study schedule for vaccines and sampling for Arm 1, 2, 3 and 4

| **Visit number**  PCV15-20 Monitoring plan - Version: 1.1. Index: OVG 2102. Printed: 26-Jun-2025 09:24 | **V1** | **V2** | **V3** | **V3 (A)** | **V4** | **V5** |
| --- | --- | --- | --- | --- | --- | --- |
| **Participant age*** | 2 months | 3 months | 4 months | 5 months | 12 months | 13 months + |
| **Blood sample for Arms 1, 2, and 4** | - | - | Yes |  | - | Yes |
| **Blood sample for Arm 3** |  |  |  | Yes |  | Yes |
| **Nasal sample (mucosal lining fluid) for Arms 1, 2 and 4** |  | Yes | Yes |  | - | Yes |
| **Nasal sample (mucosal lining fluid) for Arm3** | Yes | Yes |  | Yes |  | Yes |
| **Arms 1, 2,3,4** | 6:1 MenB Rotavirus | 6:1  Rotavirus | 6:1 MenB |  |  | - |
|  |  |  |  |  | MenB |  |
|  |  |  |  |  |  |  |
|  |  |  |  |  | MMR |  |
| **Arm 1** | - | PCV15 | - |  | PCV15 | - |
| **Arm 2** | - | PCV20 | - |  | PCV20 | - |
| **Arm 3** | PCV20 |  | PCV20 |  | PCV20 | - |
| **Arm 4** | - | PCV13 | - |  | PCV13 | - |

Study schedule for vaccines and sampling for Arm 1r4, 2r4 and 4r4.

| **Visit number** | **V1** | **V2** | **V3** | **V3 (A)** | **V4** | **V5** |
| --- | --- | --- | --- | --- | --- | --- |
| **Participant age*** | 2 months | 3 months | 4 months | 5 months | 12 months | 13 months  + |
| **Blood sample** |  |  |  | Yes |  | Yes |
| **Nasal sample (mucosal lining fluid)** |  |  | Yes | Yes |  | Yes |
| **Arms 1r4, 2r4 and**  **4r4** | 6:1 MenB Rotavirus | 6:1 MenB Rotavirus |  |  | MenB |  |
|  |  |  | 6:1 |  |  |  |
|  |  |  |  |  |  |  |
|  |  |  |  |  |  |  |
|  |  |  |  |  | MMR |  |
| **Arm 1r4** |  |  | PCV15 |  | PCV15 |  |
| **Arm 2r4** |  |  | PCV20 |  | PCV20 |  |
| **Arm 4r4** |  |  | PCV13 |  | PCV13 |  |

Monitoring Plan

Study: Evaluation of higher valency pneumococcal vaccines (PCV15/PCV20) compared to PCV13

given in homologous schedules at 3 months and 12 months (“1+1” schedule) and 2 months, 4

months and 12 months (“2+1” schedule) in infants.

OVG reference: OVG2023/10

IRAS Project ID: 1009039

Chief Investigator: Dr. Dominic Kelly

**Table 2 Objectives and Outcomes Measures:**

| **Objectives**  PCV15-20 Monitoring plan - Version: 1.1. Index: OVG 2102. Printed: 26-Jun-2025 09:24 | **Outcome Measures** | **Timepoint(s) of evaluation of this outcome measure (if applicable)** |
| --- | --- | --- |
| **Primary Objective:**  To determine if the PCV13 serotype-specific serum IgG concentrations is non-inferior in homologous arms 1/1r4 & 2/2r4 at 13 months in comparison to the control arm 4/4r4. | PCV13 serotype-specific serum IgG concentration. | At 13 months of age (at least 4 weeks after administration of the last dose of PCV vaccine). |
| **Secondary Objectives:** To determine if the PCV13 serotype-specific serum IgG concentrations is non-inferior in arm 3 in comparison to the control arm 4/4r4. | PCV13 serotype-specific serum IgG concentration. | At 13 months of age (at least 4 weeks after administration of the last dose of PCV vaccine). |
| To characterise the seven additional PCV20 serotype- specific serum IgG concentrations following homologous PCV schedules in arm 1/1r4, 2/2r4 and 3 in comparison to the control arm 4/4r4 | PCV20 serotype-specific serum IgG concentrations (Serotypes 8, 10A, 11A, 12F, 15B, 22F and 33F) | At 13 months of age (at least 4 weeks after administration of the last dose of PCV vaccine) |
| To determine the safety and reactogenicity of different schedules with PCV15 (1+1) and PCV20 (1+1) and (2+1) | Solicited AEs Unsolicited AEs SAEs | within 7 days  within 28 days  During the whole study period |
| **Exploratory Objectives:**  To determine if vaccine induce  IgG responses at the mucosa (nasal) and if antibody levels in the nose correlate with levels in blood. | PCV13 serotype specific nasal IgG GMC against some of the PCV serotypes (sample volume depending) | At 13 months of age (at least 4 weeks after administration of the last dose of PCV vaccine). |

Monitoring Plan

Study: Evaluation of higher valency pneumococcal vaccines (PCV15/PCV20) compared to PCV13

given in homologous schedules at 3 months and 12 months (“1+1” schedule) and 2 months, 4

months and 12 months (“2+1” schedule) in infants.

OVG reference: OVG2023/10

IRAS Project ID: 1009039

Chief Investigator: Dr. Dominic Kelly

| To determine the PCV20 serotype-specific IgG concentration following one dose of PCV13, PCV15 or PCV20.  PCV15-20 Monitoring plan - Version: 1.1. Index: OVG 2102. Printed: 26-Jun-2025 09:24 | PCV20 serotype-specific serum IgG concentrations | At 4/5 months. |
| --- | --- | --- |
| To determine the PCV20 serotype-specific IgG concentration following two doses of PCV20 (2+1) schedule. | PCV20 serotype-specific serum IgG concentrations | At 5 months. |
| To detect pneumococcal colonisation and serotypes present. | Molecular microbiology (fluidic qPCR for detection of lytA, paiB and serotyping) | At 13 months of age (at least 4 weeks after administration of the last dose of PCV vaccine). |
| To determine immunogenicity against concomitant vaccines given as part of the infant routine immunisation schedule | IgG Antibody GMCs against pertussis antigen (pertussis toxin, filamentous hemagglutinin and pertactin), diphtheria toxoid, tetanus toxin and Hib capsular polysaccharide;  SBA GMT against MenB | At 13 months of age (at least 4 weeks after administration of the last dose of PCV vaccine). SBA GMTs against MenB at 4/5 months (at least 4 weeks after second dose of MenB vaccine) and 13 months. |
| To compare immunological responses to PCV post priming and post boosting dose for infants given PCV vaccines at 4 and 12 months of age to infants given PCV vaccines at 3 and 12 months of age. | PCV serotype-specific serum IgG concentrations | 4 or 5 months of age (at least 4 weeks post first dose of PCV vaccine) and at 13 months of age (at least 4 weeks post second dose of PCV vaccine) |

**3.	STUDY RISKS/MITIGATION**

A risk assessment has been produced by the clinical trials unit (OVG 2057) which is specific for this trial and has identified key areas of risk. These include IMP management, participant safety, external trial site oversight, data management, recruitment, randomization, sampling procedures and staffing.

Risks identified in the risk assessment are addressed in this monitoring plan.

Monitoring Plan

PCV15-20 Monitoring plan - Version: 1.1. Index: OVG 2102. Printed: 26-Jun-2025 09:24

Study: Evaluation of higher valency pneumococcal vaccines (PCV15/PCV20) compared to PCV13 given

in homologous schedules at 3 months and 12 months (“1+1” schedule) and 2 months, 4 months and 12

months (“2+1” schedule) in infants.

OVG reference: OVG2023/10

IRAS Project ID: 1009039

Chief Investigator: Dr. Dominic Kelly

**4.	SOURCE DOCUMENTS AND DATA**

Source data are defined as the location where data is first recorded (e.g. hospital records, recorded data from the immunization child booklet.

Case Report Forms may also be considered as source documents where specified in the protocol. For this study, REDCap entries and electronic diary cards will be considered source data where the information is not recorded elsewhere as detailed above.

The source documents for this trial are listed in Appendix 1

Monitoring Plan

Study: Evaluation of higher valency pneumococcal vaccines (PCV15/PCV20) compared to PCV13 given

in homologous schedules at 3 months and 12 months (“1+1” schedule) and 2 months, 4 months and 12

months (“2+1” schedule) in infants.

OVG reference: OVG2023/10

IRAS Project ID: 1009039

Chief Investigator: Dr. Dominic Kelly

**5. COMMUNICATION**

Day-to-day communication on study progress will take place primarily between:

a. (Monitor) and the following site contacts:
a. Rachel White **(Oxford Lead nurse)**

Jill Muller **(Oxford Lead nurse)**

External sites team **(Bristol, Nottimgham, St.Georges, Southamptom)**

PCV15-20 Monitoring plan - Version: 1.1. Index: OVG 2102. Printed: 26-Jun-2025 09:24

The following should be copied into all communication:

OVG QA team

Denis Murphy **(OVG Project Manager)**

Communication by email/telephone/video call will be planned approximately monthly throughout the study.

This frequency will necessarily vary according to the level of study activity.

*NB. It is the Site’s responsibility to inform the Monitor when each monitoring trigger point has been reached. However, the monitor should ensure that the site is contacted on a regular basis after this in order to keep track of study progress and arrange monitoring visits accordingly.*

In the event of monitoring/data issues remaining unresolved between a. and b above, escalation of the issue should then be to the CI and site PI and a senior member of the OVG QA team.

The Principal Investigator will ensure that the Monitor is informed of any relevant site staff changes as soon as possible.

Significant changes to this Monitoring Plan will be made by agreement between the Chief Investigator and the monitor.

Monitoring Plan

Study: Evaluation of higher valency pneumococcal vaccines (PCV15/PCV20) compared to PCV13 given

in homologous schedules at 3 months and 12 months (“1+1” schedule) and 2 months, 4 months and 12

months (“2+1” schedule) in infants.

OVG reference: OVG2023/10

IRAS Project ID: 1009039

Chief Investigator: Dr. Dominic Kelly

**6. PROCEDURE**

Monitoring will be carried out by trained personnel from the OVG QA team according to Good Clinical Practice, using OVG Standard Operating Procedures (SOPs) and according to the schedule below.

Monitoring visits will ideally be on site, however, may also be conducted remotely. Remote visits will include the sharing of paper source documents (redacted where necessary if required by local policy), review of REDCap data and video meeting with the trial team, which may include video review of facilities such as IMP and sample storage, as required.

At least either the site initiation visit or the first routine monitoring visit will be on site.

PCV15-20 Monitoring plan - Version: 1.1. Index: OVG 2102. Printed: 26-Jun-2025 09:24

A monitoring report will be completed by the monitor following each scheduled monitoring visit. Reports generated by OVG monitors will be reviewed by a senior OVG QA Manager.

Reports should be completed and forwarded to the senior OVG QA Manager within 2 weeks of the monitoring visit. The monitoring finding log should be forwarded to the site within 5 working days of the visit, with any urgent findings discussed with the trial team on the day of the visit.

As part of the report review process, the monitor and senior OVG QA Manager will discuss any trends or significant findings that may require additional action and feed these back to the site and coordinating team in Oxford.

The monitor will also meet periodically with the QA and Regulatory lead to discuss progress with the trial monitoring.

Monitoring Plan

Study: Evaluation of higher valency pneumococcal vaccines (PCV15/PCV20) compared to PCV13 given

in homologous schedules at 3 months and 12 months (“1+1” schedule) and 2 months, 4 months and 12

months (“2+1” schedule) in infants.

OVG reference: OVG2023/10

IRAS Project ID: 1009039

Chief Investigator: Dr. Dominic Kelly

**Table 3: Study monitoring visit schedule**

PCV15-20 Monitoring plan - Version: 1.1. Index: OVG 2102. Printed: 26-Jun-2025 09:24

| **Visit Code** | **Trigger** |
| --- | --- |
| Site Initiation Visit 1 (MV1 Site) | Prior to enrollment of the first participant at each site |
| Site Monitoring 2 (MV2 Site) | Following 6^th^ participant enrolled at site. |
| Critical data review visit (MV3 Remotely) | Following 100 participants enrolled across sites. |
| Site Monitoring 3 (MV4 Site) | Following 450 participants enrolled across sites |
| Site monitoring 4 (MV5 site) | Following last visit of last participant |
| Close Out Visit 5 (MV6 site) | Following study end at site |

The schedule listed above outlines the expected monitoring activity, however both the number of visits and the timepoint may be amended to allow for monitoring visits to be conducted based on recruitment rates and site/sponsor requirements.

Monitoring Plan

Study: Evaluation of higher valency pneumococcal vaccines (PCV15/PCV20) compared to PCV13 given

in homologous schedules at 3 months and 12 months (“1+1” schedule) and 2 months, 4 months and

12 months (“2+1” schedule) in infants.

OVG reference: OVG2023/10

IRAS Project ID: 1009039

Chief Investigator: Dr. Dominic Kelly

**6.1 Site Initiation Visit**

An initiation event will be arranged once all relevant approvals have been obtained and prior to screening of

the first study participant. The SIV will be reported using OVG002-F1 Site Initiation Visit Checklist/Report and a list of findings forwarded to the site for action, using OVG002-F11 OVG Monitoring Findings Log.

The monitor will notify the site and coordinating team in Oxford urgently if any findings prevent the site from opening to recruitment.

**6.2 Site Monitoring Visits**

PCV15-20 Monitoring plan - Version: 1.1. Index: OVG 2102. Printed: 26-Jun-2025 09:24

Monitoring visits will be conducted according to the schedule above and reported using OVG002-F6 Monitoring Visit Report. A list of findings will be forwarded to the site for action, using OVG002-F11 OVG Monitoring Findings Log.

**Participant enrolment status** and overall progress of the study will be reviewed at each site monitoring visit and discussed with study staff.

**Investigator Site File/Trial Master File:** this will be checked at appropriate visits for updates to ensure that current essential documents are filed or signposted. The list of documents to be checked is included in the OVG002-F6 Monitoring Visit Report. The TMF in Oxford will also be reviewed at least once during the trial and at the end of the trial.

The TMF and ISF will be reviewed in full at the start and end of the trial and for updated documents during the active phase of the trial.

**Informed consent** Informed consent and re-consent will be reviewed by the monitor for the 100% of participants enrolled in the study.

**Investigational Medicinal Product** accountability, supply and storage will also be monitored at each visit (critical data review and on site) during the vaccination phase of the trial. Accountability records for those participants subject to SDV will be reviewed. Non-IMP vaccine accountability will also be reviewed.

**Source data verification (SDV)** will be reviewed for any paper or electronic source data that is transcribed to the trial database for 20% of participants (i.e. is not directly entered into the REDCap trial database). These participants will be selected at random, but consideration will be given to reviewing participants enrolled early in the study or following significant amendments to the study protocol. The purpose of the SDV will be to check for accurate transcription of source data onto the study database and to check this data also meets the requirements of the protocol. This will include the transfer of data from the eDiary to the main database if required by the protocol. SDV for accuracy of safety blood data entry (between source document and REDCap eCRF entry) will not be required if double data entry is used. Discrepancies between blood entry 1 and 2 will be reviewed for all participants as part of the critical data review.

Source data verification will also include review of safety blood results to ensure these are reviewed and actioned in a timely fashion, informed consent and vaccine accountability.

SDV will be increased if significant or recurring errors are identified.

Monitoring Plan

Study: Evaluation of higher valency pneumococcal vaccines (PCV15/PCV20) compared to PCV13 given

in homologous schedules at 3 months and 12 months (“1+1” schedule) and 2 months, 4 months and

12 months (“2+1” schedule) in infants.

OVG reference: OVG2023/10

IRAS Project ID: 1009039

Chief Investigator: Dr. Dominic Kelly

**Sample collection and storage review** will include a review of data to confirm that samples are being collected according to the protocol, that they are being stored in such a way as to maintain their integrity and that they can be traced from collection, through storage to shipping and analysis. Five immunology samples will be selected at random to review full traceability. This will be recorded on OVG002-F6 Monitoring Visit Report.

**Critical data review** will also be undertaken at two timepoints as per visit schedule to review 100% of critical data in REDCap for all participants. This will include, but not be limited to, eligibility data, AE and SAE data and a review of randomisation vs administration. This review will be undertaken using a REDCap data extract and QA application. Queries will be raised in REDCap and a summary email circulated to the coordinating team in Oxford for review. The review will be reported as part of the central monitoring reports.

PCV15-20 Monitoring plan - Version: 1.1. Index: OVG 2102. Printed: 26-Jun-2025 09:24

Time will be allowed during or after the monitoring visit for discussion between the monitor and the site staff in order to resolve any issues arising from the visit and correct any errors. Issues not addressed and resolved during the visit will be recorded in the visit report and query log for resolution prior to the next monitoring visit.

The monitor should meet with the PI, if possible, either in person or via telephone/video call. All correspondence regarding monitoring findings should include the PI copied in, ensuring that no unblinded information is included in emails or TEAMS chats.

Following receipt of the monitoring query log, the site contact will inform the monitor once all queries have been completed and the monitor will respond to all proposed resolutions. Communication between the site contact and monitor will continue until the query can be closed.

**6.3 Close-out Visit**

Once all study procedures have been completed, a close-out visit will be performed and reported using OVG002-F7 Close Out Visit Checklist. The close-out visit may be conducted in person at the site or may be conducted using remote working procedures agreed with the trial site(s).

Monitoring Plan

Study: Evaluation of higher valency pneumococcal vaccines (PCV15/PCV20) compared to PCV13 given

in homologous schedules at 3 months and 12 months (“1+1” schedule) and 2 months, 4 months and

12 months (“2+1” schedule) in infants.

OVG reference: OVG2023/10

IRAS Project ID: 1009039

Chief Investigator: Dr. Dominic Kelly

**7. MONITORING RESOURCE**

The monitoring of this study is being undertaken by staff employed by Oxford Vaccine Group. Table 4, below, provides an estimate of the number of hours required to complete all monitoring activity. This is to be used as a guide for the trial team to support management of the study budget.

Monitors should also keep a record of hours used when performing monitoring activity to also support the above budget management and guide assessment of future monitoring resources.

**Table 3 Monitoring Resource**

PCV15-20 Monitoring plan - Version: 1.1. Index: OVG 2102. Printed: 26-Jun-2025 09:24

| **Monitoring activity** | **Estimated hours per unit** | **Total estimated hours** |
| --- | --- | --- |
| Development, review and approval of monitoring plan | 8 hours | 8 hours |
| Monitor training | 4 hours | 8 hours |
| Monitoring visits (including SIV, site, critical data  review and COV) and including preparation, conducting activity and report writing/review | 16 hours per visit for 7 sites | 672 hours |
| Monitoring correspondence | 0.5 per month | 42 |
| Oxford TMF review | 8 hours per visit | 16 hours |
| Travel time | 6 hours per site visit (up to 6 visits) | 216 |
| **Total (approximate)** |  | **962** |

8. **REVISION HISTORY**

| **Review date** | **Reviewer** | **Summary of changes** | **Associated amendment or protocol update** | **Revision type (new/major/minor )** |
| --- | --- | --- | --- | --- |
| Sept 2024 | QA team | New document | Protocol V1.0 initial approval | New |
| June 2025 | QA team | Changes in group allocation | SA03 | Minor |
|  |  |  |  |  |

Monitoring Plan

Study: Evaluation of higher valency pneumococcal vaccines (PCV15/PCV20) compared to PCV13 given

in homologous schedules at 3 months and 12 months (“1+1” schedule) and 2 months, 4 months and

12 months (“2+1” schedule) in infants.

OVG reference: OVG2023/10

IRAS Project ID: 1009039

Chief Investigator: Dr. Dominic Kelly

Appendix 1

| **Main Clinical Database**  PCV15-20 Monitoring plan - Version: 1.1. Index: OVG 2102. Printed: 26-Jun-2025 09:24 | | | | | | |
| --- | --- | --- | --- | --- | --- | --- |
| **REDCap Structure** | | | | **Source data location** | |  |
| **Event Name** | **CRF** | **CRF Name** | **Double Data Entry?** | **In REDCap?** | **Source documents if**  **"No"** | **Critical Data**  **(Y/N)** |
| Participant ID | 1 | Participant ID | No | Yes |  | N |
| Randomisation | 1 | Randomisation | No | Yes |  | Y |
| Vaccination | 1 | Visit 1 Pre-Vaccination | No | Yes |  | Y |
|  | 2 | Visit 1 Vaccination | No | Yes |  | Y |
|  | 3 | Visit 1 Post-vaccination | No | Yes |  | Y |
|  | 4 | Visit 2 Prevaccination | No | Yes |  | Y |
|  | 5 | Visit 2 Vaccination | No | Yes |  | Y |
|  | 6 | Visit 2 Post-vaccination | No | Yes |  | Y |
|  | 7 | Visit 3 Prevaccination | No | Yes |  | Y |
|  | 8 | Visit 3 Vaccination | No | Yes |  | Y |
|  | 9 | Visit 3 Post-vaccination | No | Yes |  | Y |
|  | 10 | Visit 4 Prevaccination | No | Yes |  | Y |
|  | 11 | Visit 4 Vaccination | No | Yes |  | Y |
|  | 12 | Visit 4 Post-vaccination | No | Yes |  | Y |
| Follow-up Visit | 1 | Visit 3 A | No | Yes |  | N |
|  | 2 | Visit 5 | No | Yes |  | N |
| Concomitant Medications | 1 | Concomitant Medications and Vaccination | No | Yes |  | Y |
| Adverse Event | 1 | Adverse Events | No | Yes |  | Y |
| Serious Adverse Event | 1 | Serious Adverse Event | No | Yes/No | Paper back up SAE CRF | Y |
| Diary | 1 | Diary | No | Yes/paper back up incase Redcap fails | Paper Diary | Y |
| Unscheduled or Extra Visits | 1 | Unscheduled or Extra Visit | No | Yes |  | Y |
| Biobank | 1 | Biobank Consent | No | Yes |  | N |
| Medical History | 1 | Medical History | No | Yes |  | Y |
| Blood samples | 1 | Blood results | Yes | No | Blood results printout | Y |
| Discontinuation | 1 | Discontinuation | No | Yes |  | N |
| Investigator Comments | 1 | Investigator Comments | No | Yes |  | Y |

Monitoring Plan

PCV15-20 Monitoring plan - Version: 1.1. Index: OVG 2102. Printed: 26-Jun-2025 09:24

Study: Evaluation of higher valency pneumococcal vaccines (PCV15/PCV20) compared to PCV13 given

in homologous schedules at 3 months and 12 months (“1+1” schedule) and 2 months, 4 months and

12 months (“2+1” schedule) in infants.

OVG reference: OVG2023/10

IRAS Project ID: 1009039

Chief Investigator: Dr. Dominic Kelly

End of Study	1	End of Study	No	Yes	N
